# A Synthesis of 4-Quinolone *N*-Oxides and NMR Evidence of Their Protonation-Assisted Enolisation

**DOI:** 10.3390/molecules31101680

**Published:** 2026-05-15

**Authors:** Plamen Angelov, Yordanka Sapundzhieva, Francisco Alonso, Paraskev Nedialkov

**Affiliations:** 1 University Centre on Tautomeric Research and Education in Science and Technology (ERA Chair UCTREST), University of Plovdiv Paisii Hilendarski, 24 Tsar Asen Str., 4000 Plovdiv, Bulgaria; danimollova1976@uni-plovdiv.bg; 2 Department of Organic Chemistry, University of Plovdiv Paisii Hilendarski, 24 Tsar Asen Str., 4000 Plovdiv, Bulgaria; 3 Instituto de Síntesis Orgánica and Departamento de Química Orgánica, Facultad de Ciencias, Universidad de Alicante, Apdo. 99, 03080 Alicante, Spain; falonso@ua.es; 4 Department of Pharmacognosy, Faculty of Pharmacy, Medical University of Sofia, 2 Dunav Str., 1000 Sofia, Bulgaria; pnedialkov@pharmfac.mu-sofia.bg

**Keywords:** *N*-hydroxy-4-quinolones, 4-hydroxyquinoline-*N*-oxides, HQNO, β-enaminones, heterogeneous catalytic hydrogenation, tautomerism

## Abstract

An operationally simple procedure for the synthesis of 2-alkyl-4-quinolone *N*-oxides, relying on controlled platinum-catalyzed partial hydrogenation of 2-nitrobenzoyl enamines, has been developed. The *Pseudomonas aeruginosa* metabolite 2-heptyl-4-quinolone-*N*-oxide (HQNO) and four analogous products have been prepared in good yield and high purity by this method. All products showed ampholytic properties, with a tendency to form isolable organic-soluble hydrochlorides by switching from the *N*-hydroxy-4-quinolone to 4-hydroxyquinoline-*N*-oxide tautomeric form upon partitioning between dichloromethane and 1M aqueous HCl. In basic medium, on the other hand, water-soluble salts of the *N*-hydroxy-4-quinolone tautomers were formed. NMR measurements indicate pH-dependent equilibrium with fast exchange between the 4-quinolone and the protonated 4-quinolinol tautomer.

## 1. Introduction

**Figure 1:**
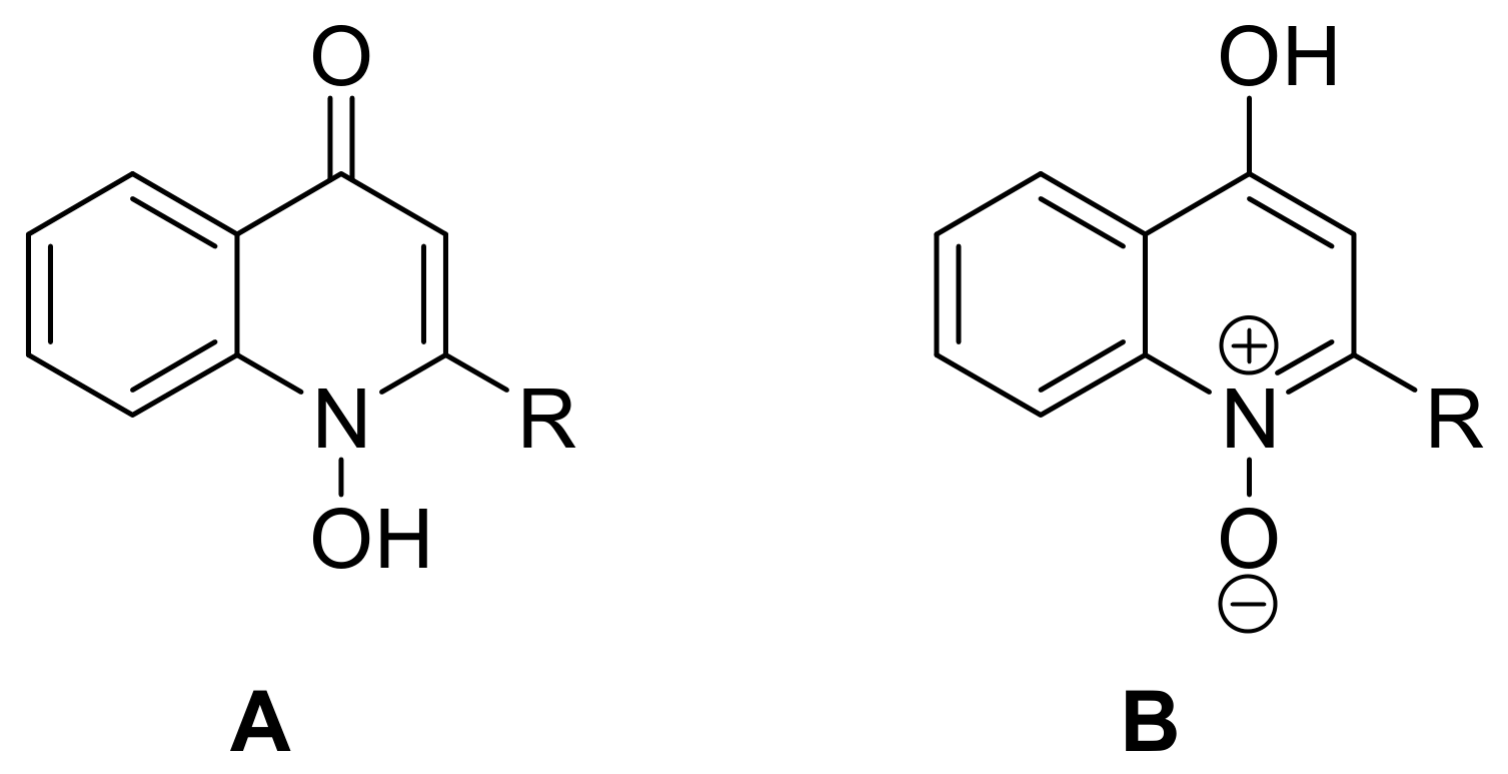
Possible tautomeric forms of AQNOs: *N*-hydroxy-4-quinolone (**A**) and 4-hydroxyquinoline *N*-oxide (**B**).

The 2-Alkyl-4(1*H*)-quinolones (AQs) are a class of quorum-sensing signal molecules that coordinate the production of virulence factors in *Pseudomonas aeruginosa* and related species [1]. Their *N*-oxides (AQNOs), on the other hand, function as toxins and weapons in bacterial interspecies competition [2]. There are two possible tautomeric forms of AQNOs (Figure 1), and for this reason they are also referred to as *N*-hydroxy-4-quinolones and 4-hydroxyquinoline-*N*-oxides (or 4-quinolinol-*N*-oxides), respectively. Although the oxidized 4-quinolone tautomer is actually a *N*-hydroxy derivative (Figure 1A), the term “4-quinolone *N*-oxide” has gained widespread acceptance as a universal reference to this type of compounds, disregarding the precise tautomeric form.

**Scheme 1:**
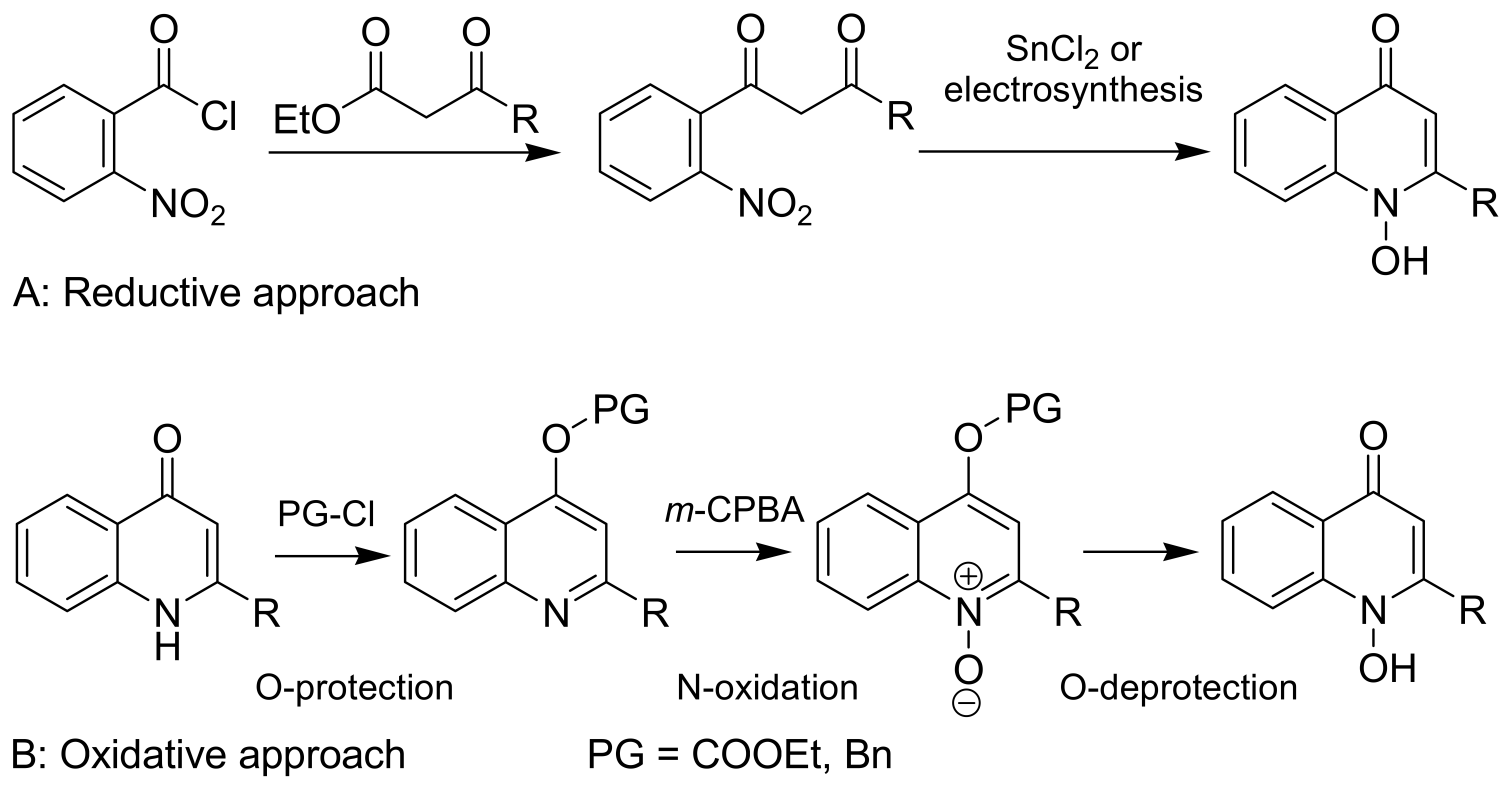
General synthetic approaches to 2-alkyl-*N*-hydroxy-4-quinolones – reductive [9,16] and oxidative [9,10,11,12,13,14,15].

Relatively few bacterial species of genera such as *Pseudomonas*, *Burkholderia*, *Arthrobacter*, *Stigmatella*, and *Rhodococcus* produce 2-alkyl-4-quinolone *N*-oxides with antibiotic activities [3,4,5]. In a recent discovery, *Chitinivorax tropicus* has also been found to produce compounds of this type [6]. The widely accepted model of their antibiotic effect is based on the disruption of the respiratory chain of the competing microorganisms [7,8]. The interesting biological profile of these compounds has motivated the development of synthetic methods for the preparation of natural AQNOs and structural analogs as potential antibacterials. Until now, two general approaches to the synthesis of AQNOs have been reported in the literature, both pioneered by Cornforth and James [9] (Scheme 1). The first one includes the preparation of nitrobenzoylketones and their partial reduction with SnCl_2_, followed by spontaneous cyclization to AQNOs. The second approach extends the Conrad–Limpach synthesis of AQs with three additional steps (*O*-protection/*N*-oxidation/*O*-deprotection) to finally give AQNOs. Due to its inefficiency, the former approach has fallen out of favor, while the latter has been preferred in virtually all studies of bacterial and related AQNOs ever since [10,11,12,13,14,15]. Only recently has an efficient electrochemical procedure for the partial reduction in nitrobenzoylketones been published [16], which holds promise for the practical application of the reductive approach.

In the course of our ongoing project on the chemistry of 4-quinolones, we have demonstrated the synthesis of some 3-carboxamide analogs of the AQNOs by partial reduction of 2-nitrobenzoylated β-enamino amides under *Pd*-catalyzed transfer hydrogenation conditions [17]. These conditions, however, did not provide complete control over the degree of hydrogenation and were not compatible with chlorinated substrates. Further, the structurally simpler natural AQNOs (with no substituent at C3) were completely out of the scope of this method because of the poor control on the degree of hydrogenation.

## 2. Results and Discussion

**Scheme 2:**
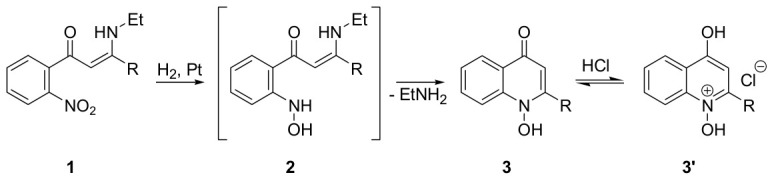
Synthesis of AQNOs. Reagents and conditions: H_2_ (balloon), 5 wt% Pt/Al_2_O_3_, *n*-BuNH_2_, DMSO, isopropanol, 24 h, r.t.

Considering the drawbacks of our previously published procedure, and taking into account the general interest in convenient preparative methods for AQNOs, we continued our efforts in this direction by screening a variety of conditions for chemoselective partial reduction in the nitro group in aromatic substrates. We were particularly interested in developing a procedure for the partial reduction of 2-nitrobenzoyl enamines **1** (Scheme 2), which are available through the convenient acylation/decarbamoylation of β-enamino amides [17]. The required procedure was expected to stop the reduction in the target substrates at the hydroxylamine level (**2**) and was also expected not to reduce the subsequently formed products **3** (Scheme 2).

**Scheme 3:**
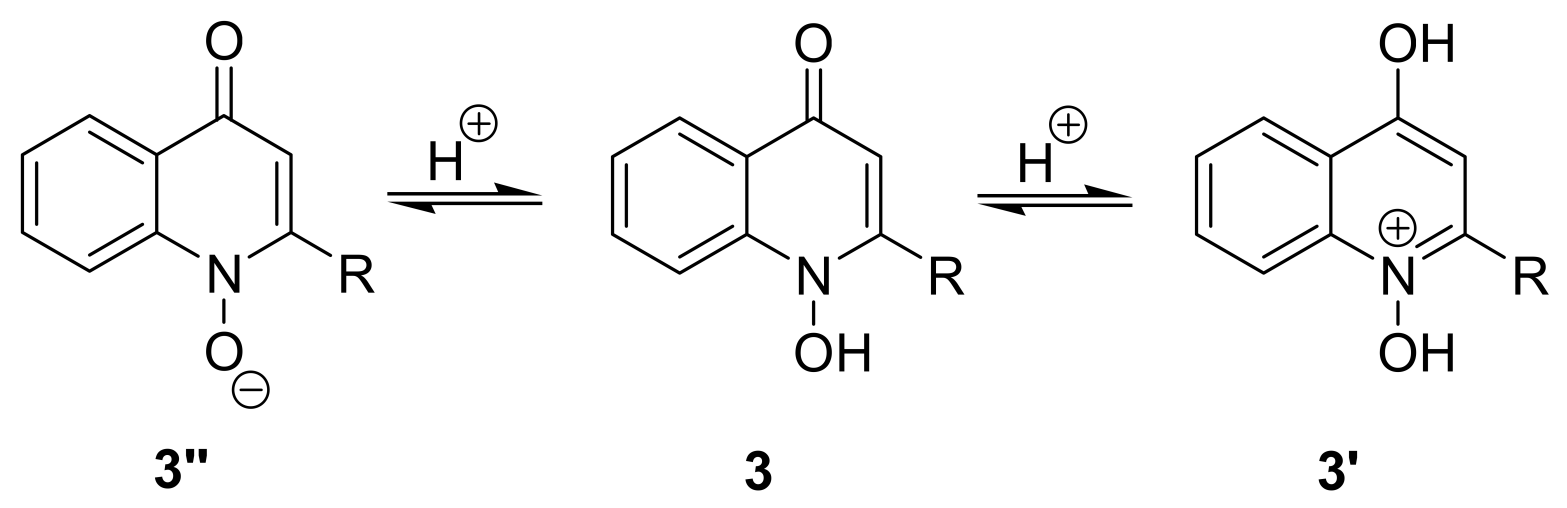
Ampholytic properties of AQNOs.

In the event, we found that the conditions reported by Takenaka and colleagues for the preparation of aromatic hydroxylamines [18] gave excellent results in the context of AQNO preparation from 2-nitrobenzoyl enamines **1**. This partial hydrogenation procedure is carried out with H_2_ at atmospheric pressure and uses a combination of DMSO and *n*-butylamine to fine-tune the activity of the alumina-supported *Pt* catalyst. When the 2-nitrobenzoyl enamines **1** were subjected to these conditions in isopropanol as the solvent, the reaction led directly to the desired *N*-hydroxy-4-quinolones **3** in very good yields and purity (Scheme 2, Table 1). The intermediately formed hydroxylamines **2** cyclized spontaneously and could not be isolated. The products **3** were easily isolated after filtration of the catalyst, evaporation of the solvent under reduced pressure, and a simple extractive workup with aqueous HCl to remove any residual DMSO and *n*-butylamine. This procedure provided the desired products as the 4-quinolinol tautomers **3′**, except for the isobutyl derivative, which had to be further purified by chromatography on a short silica gel column with diethyl ether/methanol (10:1) as the eluent and appeared as the 4-quinolone tautomer **3b**. Later, we used the developed synthetic procedure to synthesize the 2-nonyl analog **3e** in the context of another work [19] and realized that the tautomeric form of the isolated product is affected by silica gel chromatography, regardless of the substituent at C2. At this stage, we did not realize that the 4-quinolinol tautomers **3′** were actually isolated as HCl salts because we only measured their NMR spectra in DMSO-d_6_, and strong proton exchange involving the residual water in this solvent did not allow the measurement and integration of the OH signals. Also, ESI-MS could not distinguish between the *N*-hydroxyquinolones **3** and their hydrochlorides **3′**. Further experiments proved that all products **3** are initially obtained as *N*-hydroxy-4-quinolone tautomers and that the tautomeric switch to 4-quinolinol-*N*-oxides **3′** occurs at the expense of protonation by HCl during the extractive workup, when the products are partitioned between dichloromethane and 1M aqueous HCl. Alternatively, upon non-aqueous (and non-acidic) workup of the reaction mixture (filtration, evaporation and trituration with Et_2_O), only the *N*-hydroxy-4-quinolone tautomers **3** were isolated. Interestingly, all 4-quinolinol-*N*-oxide hydrochlorides **3′** had good solubility in CHCl_3_ or CH_2_Cl_2_ and were effectively extracted into the organic phase during acidic aqueous workup. In contrast, their *N*-hydroxy-4-quinolone counterparts **3**, especially those with shorter chains at C2 (**3a–c**), were poorly soluble in either dichloromethane or water. During silica gel chromatography, all 4-quinolinol-*N*-oxide hydrochlorides **3′** underwent deprotonation and were switched back to *N*-hydroxy-4-quinolones **3**. Similarly, deprotonation of **3′** to **3** was accomplished with saturated aqueous NaHCO_3_. However, when the deprotonation of **3′** was attempted with a stronger base, such as K_2_CO_3_ (2% aqueous solution), the quinolone was retained mostly in the aqueous phase, suggesting possible further deprotonation of **3** and formation of anions **3″**, which could be regarded as vinylogous hydroxamates (Scheme 3). This ampholytic behavior of **3** was further proved by measuring their NMR spectra in methanol-d_4_, with and without added NaOH. While all products **3** were poorly soluble in methanol and some of the weaker ^13^C signals could not be registered in this solvent, the addition of NaOH dramatically improved the solubility by formation of the **3″** salts and facilitated the NMR measurements, revealing a preference for the 4-quinolone form of the **3″** anions in basic medium. The sodium salts, formed by the reaction of **3** with an equimolar amount of NaOH in methanol, were isolated after evaporation of the solvent and further characterized by melting points (Table 1) and IR spectroscopy (Supplementary Data).

The problem of AQNO tautomerism has generally been overlooked in the literature, with either arbitrary tautomer assignment in older publications lacking sufficient spectral evidence or proper characterization provided only for the carbonyl tautomer (*N*-hydroxy-4-quinolone) in more recent works. For this reason, we paid special attention to the NMR spectra of the obtained compounds **3** and their salts **3′** and **3″** by varying the solvent and the acidity of the solution.

According to our measurements, the ^1^H-NMR signals most indicative of the tautomeric form are those of the C3-H protons, appearing at 6.00–6.50 ppm for the carbonyl tautomers **3** or at 7.00–7.15 ppm for the aromatized 4-quinolinol-*N*-oxide hydrochlorides **3′** (Table 2). The chemical shift in the OH protons is also strongly dependent on the tautomeric form, but this could only be observed in cases where both forms are CDCl_3_-soluble, such as the *n*-heptyl and *n*-nonyl analogs **3d** and **3e**. Compounds **3a–c** are soluble in CDCl_3_ as 4-quinolinol-*N*-oxide hydrochlorides **3′** but are completely insoluble in their *N*-hydroxy-4-quinolone form **3**. The OH signals could not be registered in either DMSO-d_6_ or methanol-d_4_, because of proton exchange with the solvent or residual water. The chemical shifts in the alkyl chain ^1^H-signals are affected to a smaller extent by the tautomeric state of the heterocycle, most visibly those of the directly adjacent methylenes (2.80 ppm for **3** vs. 3.00 ppm for **3′**). In the ^13^C-NMR spectra, the most significant difference between the tautomers was observed in the chemical shift in the C4 signals (170–173 ppm for a carbonyl C4 and 166–167 ppm for an aromatic C4-OH). The C2 signal was also noticeably affected by the tautomeric transition, moving from 153 to 155 ppm (**3**) to 158–159 ppm (**3′**), while the change in the C3 δ was small (less than 2 ppm), appearing at ca. 105 ppm for both tautomers (Table 3). Interestingly, all previously reported NMR spectra of the known products **3c** [12] and **3d** [11,12] have been measured in methanol-d_4_ and described as the *N*-hydroxy-4-quinolone form, with the C4-carbonyl ^13^C signal visible only in HMBC. Indeed, this signal proved to be problematic, especially in solvents capable of proton exchange, and required a high sample concentration (0.2–0.4 mol/L) in order to be registered in the ^13^C spectra. For some of the products, such concentrations of the *N*-hydroxy-4-quinolone form were attainable only at elevated temperature (70 °C in DMSO-d_6_ or 55 °C in MeOD). Measuring the ^13^C NMR spectra at high concentration helped to reveal the C4-carbonyl signal in the obtained products **3** in their neutral form, except for **3b** and **3c**, where it was registered only as an HMBC correlation to the C3-H signal. In contrast, the C4 signal was easily registered in both ionic forms of **3** (**3′** and **3″**), appearing at 166–167 ppm for the protonated quinolinol form **3′** or at 172 ppm for the deprotonated quinolone form **3″**. In one case, TFA-d_1_ was used as the solvent, completely converting quinolone **3d** into the protonated 4-quinolinol **3d’** trifluoroacetate. A comparison of the most characteristic NMR signals of compounds **3** in their neutral and ionic forms is presented in Table 2 (^1^H) and Table 3 (^13^C).

**Figure 2:**
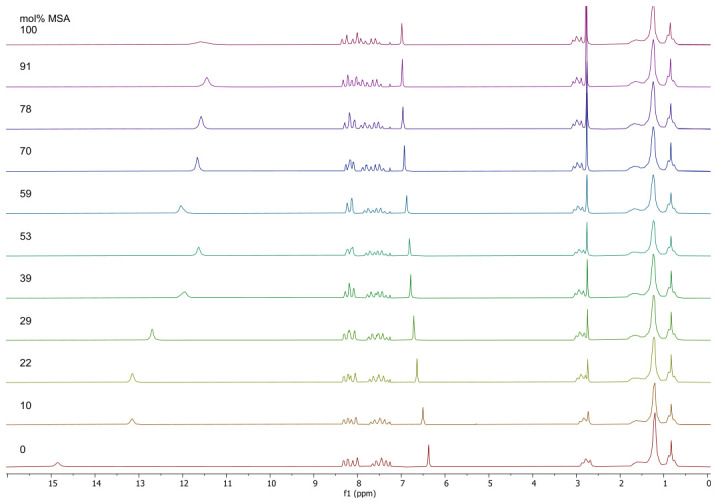
^1^H NMR spectra (80 MHz, CDCl_3_) of **3d** (HQNO) with increasing amounts of added CH_3_SO_3_H (0–100 mol%).

**Figure 3:**
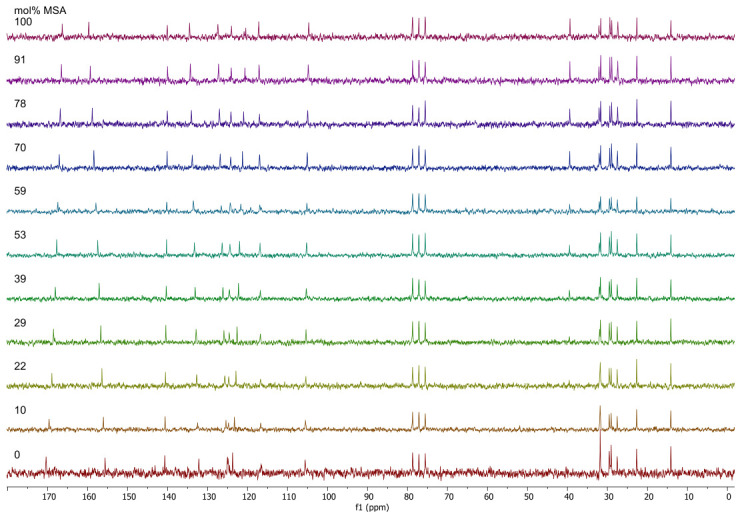
^13^C NMR spectra (20 MHz, CDCl_3_) of **3d** (HQNO) with increasing amounts of added CH_3_SO_3_H (0–100 mol%).

To better understand the transition from **3** to **3′**, a series of NMR spectra of compound **3d** (HQNO) were measured in CDCl_3_ at a concentration of 0.2 mol/L, with increasing amount of added methanesulfonic acid (MSA, 0–220 mol% relative to **3d**). With the increasing proportion of MSA, a gradual downfield shift in the C3-H signal and an upfield shift in the OH signal were observed in the ^1^H NMR spectra (Figure 2). The ^13^C NMR signals were affected to a smaller extent by the addition of MSA, most significantly those corresponding to the C-4 and C-2 positions (Figure 3). These observations suggest a dynamic proton exchange equilibrium with quick transition between the tautomeric forms, registered as a weighted average spectrum of the *N*-hydroxy-4quinolone **3** and the protonated 4-quinolinol-*N*-oxide **3′**.

**Figure 4:**
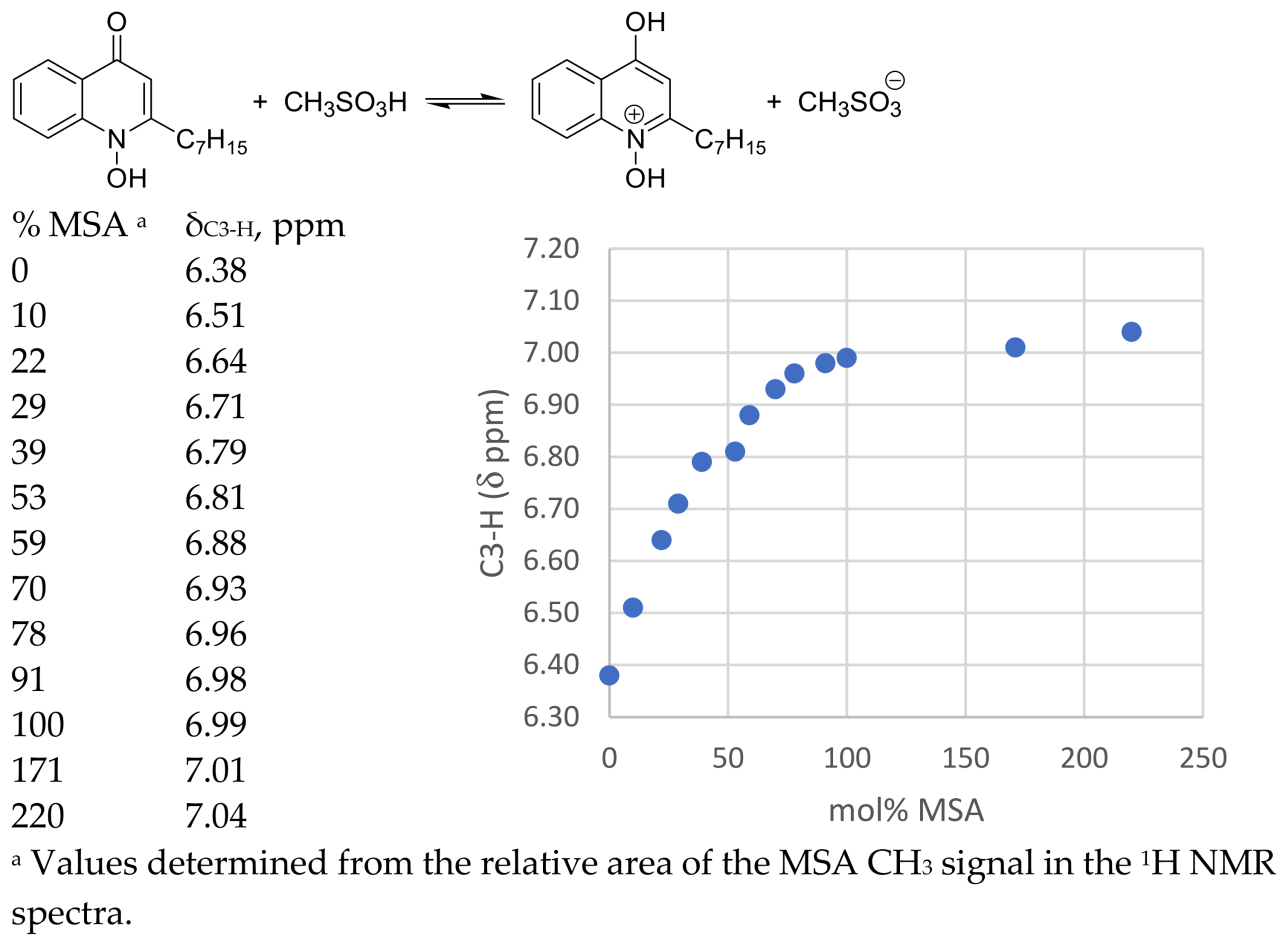
δ C3-H dependence on the mol% MSA relative to **3d**.

The correlation of δ_C3-H_ with the proportion of MSA follows a good linear pattern up to 40% MSA and deviates from linearity at higher values (Figure 4). This deviation from linearity at higher acid proportion indicates that the amount of the protonated quinolone does not directly correspond to the amount of the added acid, which can be explained in terms of nearly full MSA deprotonation when an excess of quinolone is present and only partial MSA deprotonation at proportions approaching 100%. Adding an excess of MSA further shifts the equilibrium to the right, as indicated by the last two measurements in the series.

**Figure 5:**
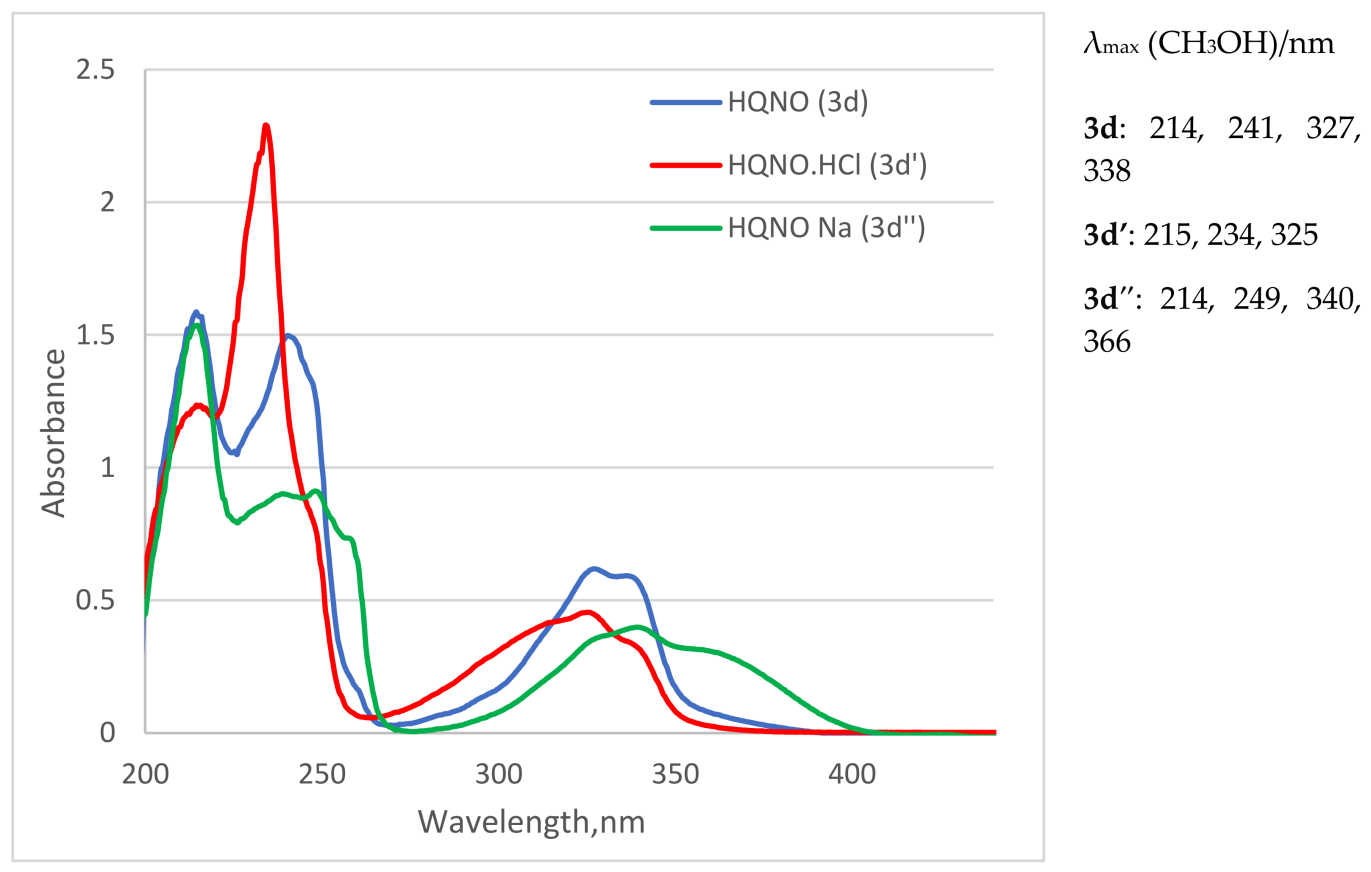
UV spectra of compound **3d** (HQNO) and its salts at 5 × 10^−5^ mol/L in methanol.

The variation in the solubility of **3** with pH change imposes significant difficulty on the comparison of all three protonation states in the same solvent by NMR. This, however, can be done by UV spectroscopy, as it operates on much lower concentrations. The UV spectra of compound **3d** and its salts **3d’**/**3d″** were measured at a concentration of 5 × 10^−5^ mol/L in methanol (Figure 5). The relative intensities and shapes of the absorption bands of the sodium salt **3d″** are similar to those of the neutral form **3d**, with λ_max_ = 214 nm and bathochromic shifts in the 340–370 nm region. The protonated form **3d’**.HCl, on the other hand, showed λ_max_ = 234 nm with significantly higher absorbance than **3d**/**3d″** and a blue shift in the long-wavelength absorption, which is in agreement with the proposed change in the electronic structure of the heterocyclic ring upon protonation. The neutral form is likely present in the solutions of both salts as a result of protolytic equilibrium with the solvent, and this is well evidenced by the residual absorption around 340 nm.

## 3. Materials and Methods

The preparation of the starting compounds **1** is described in a previous publication [17]. Unless otherwise noted, all other reagents and solvents were purchased from Sigma-Aldrich, Darmstadt, Germany, and were used as supplied. NMR spectra were recorded on a Bruker Avance NEO 400 spectrometer, operating at 400/100 MHz ^1^H/^13^C (Bruker, Billerica, MA, USA) or Magritek Spinsolve 80 spectrometer, operating at 80/20 MHz ^1^H/^13^C (Magritek, Aachen, Germany). Chemical shifts (δ, ppm) are reported downfield from TMS, using residual solvent signals as the internal standard. TLC was done on aluminum-backed Silica gel 60 sheets (Merck, Darmstadt, Germany) with KMnO_4_ staining. Melting point measurements were done in capillary tubes on KRÜSS M5000 (A.Krüss Optronic GmbH, Hamburg, Germany) automatic mp meter and are not corrected. Mass spectral measurements were performed on a Thermo Fisher Scientific Q Exactive Plus high-resolution mass spectrometer with a heated electrospray ionization source HESI-II (Thermo Fisher Scientific, Waltham, MA, USA). IR spectra were measured on an Alpha II FTIR spectrometer (Bruker Optics GmbH, Ettlingen, Germany) and UV spectra were measured on a UV VIS spectrophotometer Agilent Cary 60 (Agilent Technologies, Santa Clara, CA, USA).

Synthesis of compounds **3**, general procedure: The corresponding starting compound **1** (1 mmol) was dissolved in isopropanol (IPA) (5 mL). Then DMSO (0.026 g, 0.024 mL), *n*-butylamine (0.074 g, 0.1 mL) and 5 wt% Pt/Al_2_O_3_ (0.020 g) were added to the solution. The air in the reaction vessel was evacuated and replaced with *H_2_* with the help of a three-way stopper. The H_2_ atmosphere was kept with a balloon for the next 24 h while the reaction mixture was magnetically stirred at 25 °C. Then, the catalyst was filtered off through a pad of celite on a sintered glass funnel and rinsed thoroughly with IPA. The IPA was removed from the filtrate on a rotary evaporator under reduced pressure at 60 °C. The residue was kept under vacuum (0.1–1 mm Hg) for 1 h at r.t. to remove any residual butylamine. Then, trituration with a small amount of diethyl ether (**3c–e**) or acetone (**3a–b**) was done to facilitate crystallization and to rinse the formed solid *N*-hydroxy-4-quinolone. Once isolated, the *N*-hydroxy-4-quinolones **3** can be quantitatively converted to their corresponding 4-quinolinol-*N*-oxide hydrochlorides **3′** by partitioning between dichloromethane and dilute aqueous HCl. In a typical procedure, 1 mmol of **3** was intensely stirred in a mixture of dichloromethane (40 mL) and aqueous HCl (20 mL, approximately 1 mol/L). Then, the aqueous phase was extracted with additional 30 mL of dichloromethane, and the combined organic layers were dried with anhydrous sodium sulfate. The solvent was removed under reduced pressure, and the residue was triturated with diethyl ether to afford **3′** as an amorphous solid, except for **3b’**, which was isolated as a clear oil.

1-Hydroxy-2-propylquinolin-4(1H)-one (**3a**): yield: 152 mg (75%); m.p. 192–193 °C; ^1^H-NMR (400 MHz, CD_3_OD, δ ppm, *J* Hz): 1.07 (t, 3H, *J* = 7.5), 1.82 (sext, 2H, *J* = 7.5), 2.91 (t, 2H, *J* = 7.5), 6.32 (s, 1H), 7.49 (app t, 1H, *J* = 7.6), 7.79 (app t, 1H, *J* = 7.8), 8.07 (app d, 1H, *J* = 8.7), 8.26 (dd, 1H, *JJ* = 8.2, 0.5); ^13^C-NMR (100 MHz, CD_3_OD, δ ppm): 12.6, 20.7, 33.0, 106.2, 115.3, 124.1, 124.4, 124.5, 132.1, 140.7, 154.7, 172.8; HRMS m/z (ES+): calcd. for C_12_H_14_NO_2_^+^ [M + H]^+^ 204.1019, found 204.1019.

2-Propyl-4-hydroxyquinoline N-oxide hydrochloride (**3a’**): white solid, m.p. 154–155 °C; ^1^H-NMR (400 MHz, DMSO-d_6_, δ ppm, *J* Hz): 1.01 (t, 3H, *J* = 7.4), 1.79 (sext, 2H, *J* = 7.4), 3.07 (t, 2H, *J* = 7.4), 7.02 (s, 1H), 7.75 (m, 1H), 8.05 (m, 1H), 8.24 (app d, 1H, *J* = 8.5), 8.29 (dd, 1H, *JJ* = 8.3, 0.8); ^13^C-NMR (100 MHz, DMSO-d_6_, δ ppm): 14.1, 21.0, 33.5, 105.6, 117.2, 121.5, 124.3, 127.5, 134.7, 140.1, 158.2, 167.6; ^1^H-NMR (400 MHz, CDCl_3_, δ ppm, J Hz): 1.00 (t, 3H, *J* = 7.4), 1.74 (sext, 2H, *J* = 7.5), 2.99 (app t, 2H, *J* = 7.8), 7.09 (s, 1H), 7.57 (m, 1H), 7.83 (m, 1H), 8.06 (dd, 1H, *JJ* = 8.3, 1.0), 8.25 (app d, 1H, *J* = 8.6), 11.54 (br s, 2H); ^13^C-NMR (100 MHz, CDCl_3_, δ ppm): 13.8, 20.9, 33.9, 104.9, 117.1, 120.8, 123.9, 127.2, 134.2, 139.9, 158.4, 166.9.

1-Hydroxy-2-isobutylquinolin-4(1H)-one (**3b**): yield: 153 mg (70%), white solid, m.p. 230–231 °C (dec.); ^1^H-NMR (70 °C, 400 MHz, DMSO-d_6_, δ ppm, *J* Hz): 0.97 (d, 6H, *J* = 6.7), 2.16 (m, 1H), 2.66 (d, 2H, *J* = 7.2), 5.97 (s, 1H), 7.38 (m, 1H), 7.73 (m, 1H), 7.88 (app d, 1H, *J* = 8.4), 8.12 (ddd, 1H, *JJJ* = 8.0, 1.5, 0.4); ^13^C-NMR (70 °C, 100 MHz, DMSO-d_6_, δ ppm): 22.6, 27.2, 40.3, 108.3, 115.5, 123.9, 125.3, 125.4, 132.2, 141.0, 152.7, 171.0 (HMBC); HRMS m/z (ES+): calcd. for C_13_H_16_NO_2_^+^ [M + H]^+^ 218.1176, found 218.1171.

2-Isobutyl-4-hydroxyquinoline N-oxide hydrochloride (**3b’**): oil; ^1^H-NMR (400 MHz, CDCl_3_, δ ppm, *J* Hz): 0.91 (d, 6H, *J* = 6.6), 2.20 (m, 1H), 2.86 (d, 2H, *J* = 7.1), 7.01 (s, 1H), 7.55 (app t, 1H, *J* = 7.6), 7.82 (app t, 1H, *J* = 7.8), 8.01 (app d, 1H, *J* = 8.2), 8.25 (app d, 1H, *J* = 8.8), 10.13 (br s, 2H); ^13^C-NMR (100 MHz, CDCl_3_, δ ppm): 22.4, 27.8, 41.0, 105.8, 117.2, 120.9, 123.9, 127.3, 134.2, 140.0, 157.7, 166.5.

1-Hydroxy-2-pentylquinolin-4(1H)-one (**3c**): yield: 166 mg (72%), white solid, m.p. 184–185 °C; ^1^H-NMR (55 °C, 400 MHz, CD_3_OD, δ ppm, *J* Hz): 0.96 (t, 3H, *J* = 7.2), 1.45 (m, 4H), 1.81 (m, 2H), 2.94 (app t, 2H, *J* = 7.8), 6.34 (s, 1H), 7.50 (m, 1H), 7.81 (m, 1H), 8.09 (ddd, 1H, *JJJ* = 8.7, 1.0, 0.6), 8.27 (ddd, 1H, *JJJ* = 8.2, 1.5, 0.6); ^13^C-NMR (55 °C, 100 MHz, CD_3_OD, δ ppm): 12.7, 21.9, 27.1, 31.0, 31.2, 106.2, 115.3, 124.1, 124.4, 124.5, 132.1, 140.7, 155.0, 172.8 (HMBC); HRMS m/z (ES+): calcd. for C_14_H_18_NO_2_^+^ [M + H]^+^ 232.1332, found 232.1331.

2-Pentyl-4-hydroxyquinoline N-oxide hydrochloride (**3c’**): white solid, m.p. 139–140 °C; ^1^H-NMR (400 MHz, DMSO-d_6_, δ ppm, *J* Hz): 0.89 (t, 3H, *J* = 7.0), 1.36 (m, 4H), 1.77 (m, 2H), 3.12 (t, 2H, *J* = 7.8), 7.16 (s, 1H), 7.80 (m, 1H), 8.09 (m, 1H), 8.30 (m, 2H), 11.70 (br s, 1H); ^13^C-NMR (100 MHz, DMSO-d_6_, δ ppm): 14.2, 22.2, 27.2, 31.2, 31.7, 105.4, 117.4, 121.1, 124.2, 128.0, 135.0, 140.0, 158.9, 166.8; ^1^H-NMR (400 MHz, CDCl_3_, δ ppm, *J* Hz): 0.84 (t, 3H, *J* = 6.7), 1.31 (m, 4H), 1.69 (m, 2H), 3.00 (app t, 2H, *J* = 7.9), 7.12 (s, 1H), 7.55 (m, 1H), 7.81 (m, 1H), 8.04 (dd, *JJ* = 8.3, 2.4), 8.23 (d, 1H, *J* = 8.8), 11.70 (br s, 2H); ^13^C-NMR (100 MHz, CDCl_3_, δ ppm): 13.9, 22.2, 27.2, 31.4, 32.2, 104.9, 117.1, 120.7, 123.9, 127.3, 134.3, 139.9, 158.9, 166.7.

1-Hydroxy-2-heptylquinolin-4(1H)-one (**3d**): yield: 202 mg (78%), white solid, m.p. 159–160 °C (Lit. [9] m.p. 158–160 °C, Lit. [11] m.p. 158–159 °C); ^1^H-NMR (400 MHz, DMSO-d_6_, 70 °C, δ ppm, *J* Hz): 0.98 (t, 3H, *J* = 6.9), 1.29 (m, 4H), 1.36 (m, 4H), 1.70 (m, 2H), 2.77 (t, 2H, *J* = 7.6), 5.98 (s, 1H), 7.37 (t, 1H, *J* = 7.3), 7.72 (t, 1H, *J* = 7.7), 7.87 (d, 1H, *J* = 8.5), 8.12 (d, 1H, *J* = 7.9); ^13^C-NMR (100 MHz, DMSO-d_6_, 70 °C, δ ppm): 14.2, 22.4, 27.8, 28.8, 29.1, 31.2, 31.6, 107.1, 115.5, 123.8, 125.3, 125.4, 132.2, 141.1, 153.9; ^1^H-NMR (400 MHz, CDCl_3_, δ ppm, *J* Hz): 0.86 (t, 3H, *J* = 6.9), 1.23–1.31 (m, 8H), 1.64 (m, 2H), 2.84 (m, 2H), 6.46 (s, 1H), 7.40 (t, 1H, *J* = 7.6), 7.58 (m, 1H), 8.07 (d, 1H, *J* = 8.7), 8.30 (d, 1H, *J* = 8.2), 14.31 (br s, 1H); ^13^C-NMR (100 MHz, CDCl_3_, δ ppm): 14.1, 22.6, 27.5, 29.0, 29.5, 31.7, 31.8, 105.5, 116.5, 123.6, 124.8, 125.0, 132.1, 140.6, 155.6, 170.2; HRMS m/z (ES+): calcd. for C_16_H_22_NO_2_^+^ [M + H]^+^ 260.1645, found 260.1636.

2-Heptyl-4-hydroxyquinoline N-oxide hydrochloride (**3d’**): white solid, m.p. 122–123 °C; ^1^H-NMR (400 MHz, DMSO-d_6_, δ ppm, *J* Hz): 0.85 (t, 3H, *J* = 6.8), 1.21–1.42 (m, 8H), 1.75 (m, 2H), 3.11 (t, 2H, *J* = 7.8), 7.15 (s, 1H), 7.79 (m, 1H), 8.09 (m, 1H), 8.30 (m, 2H); ^13^C-NMR (100 MHz, DMSO-d_6_, δ ppm): 14.4, 22.5, 27.5, 28.8, 29.1, 31.6, 31.8, 105.4, 117.4, 121.1, 124.2, 127.9, 135.0, 140.0, 158.9, 166.9; ^1^H-NMR (400 MHz, CDCl_3_, δ ppm, *J* Hz): 0.85 (t, 3H, *J* = 6.9), 1.22–1.37 (m, 8H), 1.64 (m, 2H), 2.96 (app t, 2H, *J* = 8.0), 7.01 (s, 1H), 7.54 (m, 1H), 7.85 (m, 1H), 7.94 (d, 1H, *J* = 8.3), 8.26 (d, 1H, *J* = 8.7), 11.00 (br s, 2H); ^13^C-NMR (100 MHz, CDCl_3_, δ ppm): 14.0, 22.6, 27.5, 28.9, 29.4, 31.6, 32.3, 104.6, 117.2, 120.5, 123.8, 127.3, 134.4, 139.9, 158.9, 166.5.

2-Heptyl-4-hydroxyquinoline N-oxide trifluoroacetate (**3d’**): ^1^H-NMR (400 MHz, CF_3_COOD, δ ppm, *J* Hz): 0.97 (t, 3H, *J* = 6.9), 1.42 (m, 4H), 1.52 (m, 2H), 1.62 (m, 2H), 1.99 (m, 2H), 3.34 (t, 2H, *J* = 7.9), 7.22 (s, 1H), 7.93 (t, 1H, *J* = 7.7), 8.19 (m, 1H), 8.42 (d, 1H, *J* = 8.8), 8.57 (d, 1H, *J* = 8.3); ^13^C-NMR (100 MHz, CF_3_COOD, δ ppm):12.2, 21.9, 27.2, 28.3, 28.7, 31.1, 31.6, 104.4, 115.5, 119.9, 123.8, 128.5, 135.7, 139.6, 160.6, 166.2.

1-Hydroxy-2-nonylquinolin-4(1H)-one (**3e**): yield: 230 mg (80%), white solid, m.p. 146–147 °C (Lit. [9] m.p 148–149 °C, Lit. [4] m.p. 132 °C); ^1^H-NMR (400 MHz, DMSO-d_6_, 70 °C, δ ppm, *J* Hz): 0.87 (t, *J* = 6.9, 3H), 1.25–1.43 (m, 12H), 1.70 (m, 2H), 2.77 (m, 2H), 5.97 (s, 1H), 7.36 (m, 1H), 7.72 (m, 1H), 7.86 (d, *J* = 8.5, 1H), 8.12 (dd, *JJ* = 8.0, 1.5, 1H); ^13^C-NMR (100 MHz, DMSO-d_6_, 70 °C, δ ppm): 14.2, 22.4, 27.8, 29.0, 29.1, 29.1, 29.3, 31.2, 31.7, 107.1, 115.5, 123.7, 125.3, 125.4, 132.1, 141.1, 153.8; ^1^H-NMR (400 MHz, CDCl_3_, 70 °C, δ ppm, *J* Hz): 0.87 (t, 3H, *J* = 6.9), 1.20–1.34 (m, 12H), 1.62 (br s, 2H), 2.80 (br s, 2H), 6.43 (s, 1H), 7.39 (t, 1H, *J* = 7.5), 7.59 (t, 1H, *J* = 7.6), 8.08 (d, 1H, *J* = 8.4), 8.29 (d, 1H, *J* = 8.0), 13.85 (br s, 1H); ^13^C-NMR (100 MHz, CDCl_3_, 70 °C, δ ppm): 14.1, 22.7, 27.5, 29.3, 29.4, 29.5, 31.7, 31.9, 105.5, 116.5, 123.6, 124.8, 125.0, 132.1, 140.6, 155.6, 170.2; HRMS *m*/*z* (ES+): calcd. for C_18_H_26_NO_2_^+^ [M + H]^+^ 288.1958, found 288.1954.

2-Nonyl-4-hydroxyquinoline N-oxide hydrochloride (**3e’**): white solid, m.p. 103–104 °C; ^1^H-NMR (400 MHz, DMSO-d_6_, δ ppm, *J* Hz): 0.84 (t, *J* = 6.8, 3H), 1.17–1.43 (m, 12H), 1.75 (m, 2H), 3.09 (app t, *J* = 7.8, 2H), 7.08 (s, 1H), 7.77 (m, 1H), 8.07 (m, 1H), 8.25 (app d, *J* = 8.5, 1H), 8.30 (dd, *JJ* = 8.3, 1.0, 1H); ^13^C-NMR (100 MHz, DMSO-d_6_, δ ppm): 14.4, 22.6, 27.6, 29.1, 29.2, 29.3, 31.7, 31.8, 105.5, 117.3, 121.4, 124.3, 127.7, 134.8, 140.0, 158.6, 167.3; ^1^H-NMR (400 MHz, CDCl_3_, 70 °C, δ ppm, *J* Hz): 0.85 (t, 3H, *J* = 6.9), 1.16–1.29 (m, 10H), 1.34 (m, 2H), 1.63 (m, 2H), 2.97 (app t, 2H, *J* = 8.0), 7.05 (s, 1H), 7.58 (m, 1H), 7.87 (m, 2H), 8.29 (dd, 1H, *JJ* = 8.7, 0.6), 11.27 (br s, 2H); ^13^C-NMR (100 MHz, CDCl_3_, 70 °C, δ ppm): 14.09, 22.64, 27.54, 29.20, 29.38, 29.40, 31.81, 32.40, 104.51, 117.32, 120.08, 123.71, 127.65, 134.69, 139.83, 159.31, 165.90.

## 4. Conclusions

In conclusion, we have described a chemoselective and operationally simple synthesis of *N*-hydroxy-4-quinolones from 2-nitrobenzoyl enamines. Two of the obtained products (**3d**/HQNO and **3e**/NQNO) are known bacterial toxins produced by *Pseudomonas aeruginosa*. All products showed a tendency to switch from the *N*-hydroxy-4-quinolone to the 4-quinolinol-*N*-oxide tautomeric form by protonation at the carbonyl oxygen and formed isolable organic-soluble hydrochlorides upon partitioning between dichloromethane and 1M aqueous HCl. The synthetic procedure was repeatedly tested at scales ranging from 0.5 to 10 mmol and proved to be an effective and scalable method for the preparation of *N*-hydroxy-4-quinolones on a gram scale, showing consistently higher yields at larger scales. Depending on the workup conditions, either *N*-hydroxy-4-quinolones **3** or 4-quinolinol-*N*-oxide hydrochlorides **3′** could be isolated in good yield and with no need of chromatographic purification.

## Figures and Tables

**Table 1:** Isolated yields of products **3**, obtained according to Scheme 2, with melting points of the neutral products **3** compared to those of their corresponding hydrochlorides **3′** and sodium salts **3″**.

Entry	R	Yield 3, %	mp (3/3′/3″), °C
**3a**	*n*-C_3_H_7_	75	193/155/312 dec.
**3b**	*i*-C_4_H_9_	70	230/oil/365 dec.
**3c**	*n*-C_5_H_11_	72	185/140/328 dec.
**3d**	*n*-C_7_H_15_	78	160/123/264 dec.
**3e**	*n*-C_9_H_19_	80	147/104/261 dec.

**Table 2:** Characteristic ^1^H-NMR signals (δ ppm) of compounds **3** in their neutral and ionic forms.

	3		3′ (Hydrochloride)		3″ (Sodium Salt)	
	C3-H	α-CH_2_	C3-H	α-CH_2_	C3-H	α-CH_2_
**a**			7.09 ^a^	2.99 ^a^		
	5.98 ^b^	2.76 ^b^	7.02 ^b^	3.06 ^b^		
	6.32 ^c^	2.91 ^c^			6.34 ^c^	2.98 ^c^
**b**			7.01 ^a^	2.86 ^a^		
	5.97 ^b^	2.66 ^b^				
	6.31 ^c^	2.80 ^c^			6.31 ^c^	2.86 ^c^
**c**			7.13 ^a^	3.00 ^a^		
			7.16 ^b^	3.12 ^b^		
	6.34 ^c^	2.94 ^c^			6.36 ^c^	3.01 ^c^
**d**	6.46 ^a^	2.84 ^a^	7.01 ^a^	2.96 ^a^		
	5.98 ^b^	2.77 ^b^	7.15 ^b^	3.11 ^b^		
	6.34 ^c^	2.93 ^c^			6.36 ^c^	3.01 ^c^
**e**	6.42 ^a^	2.80 ^a^	7.05 ^a^	2.97 ^a^		
	5.97 ^b^	2.77 ^b^	7.08 ^b^	3.09 ^b^		
	6.34 ^c^	2.93 ^c^			6.34 ^c^	3.00 ^c^

Solvents: ^a^ CDCl_3_, ^b^ DMSO-d_6_ and ^c^ CD_3_OD.

**Table 3:** Characteristic ^13^C-NMR signals (δ ppm) of compounds **3** in their neutral and ionic forms.

	3			3′ (Hydrochloride)			3″ (Sodium Salt)		
	C2	C3	C4	C2	C3	C4	C2	C3	C4
**a**				158.4 ^a^	104.9 ^a^	166.9 ^a^			
				158.2 ^b^	105.6 ^b^	167.6 ^b^			
	154.7 ^c^	106.2 ^c^	172.8 ^c^				154.5 ^c^	107.5 ^c^	172.1 ^c^
**b**				157.7 ^a^	105.8 ^a^	166.5 ^a^			
	152.7 ^b^	108.3 ^b^	171.0 ^b^*						
							153.7 ^c^	108.7 ^c^	171.7 ^c^
**c**				158.9 ^a^	104.9 ^a^	166.7 ^a^			
				158.9 ^b^	105.4 ^b^	166.8 ^b^			
	155.0 ^c^	106.2 ^c^	172.8 ^c^*				154.7 ^c^	107.4 ^c^	172.0 ^c^
**d**	155.6 ^a^	105.5 ^a^	170.2 ^a^	158.9 ^a^	104.6 ^a^	166.5 ^a^			
	153.9 ^b^	107.1 ^b^		158.9 ^b^	105.4 ^b^	166.9 ^b^			
							154.8 ^c^	107.4 ^c^	172.0 ^c^
**e**	155.6 ^a^	105.5 ^a^	170.2 ^a^	159.3 ^a^	104.5 ^a^	165.9 ^a^			
	153.8 ^b^	107.1 ^b^		158.6 ^b^	105.5 ^b^	167.3 ^b^			
							154.8 ^c^	107.4 ^c^	172.0 ^c^

Solvents: ^a^ CDCl_3_, ^b^ DMSO-d_6_ and ^c^ CD_3_OD; * only visible in HMBC.

## Data Availability

The original contributions presented in the study are included in the article and Supplementary Materials. The raw NMR data are available for download at https://doi.org/10.5281/zenodo.19204220. Further inquiries can be directed to the corresponding author.

## Supplementary Materials

The following supporting information can be downloaded at: https://www.mdpi.com/article/10.3390/molecules31101680/s1, NMR, HRMS and IR spectra of the synthesized compounds.

## Abbreviations

AQs	2-Alkyl-4(1*H*)-quinolones
AQNOs	2-Alkyl-4-quinolone *N*-Oxides
MSA	Methanesulfonic acid
NMR	Nuclear magnetic resonance
HRMS	High-resolution mass-spectrometry
IPA	Isopropyl alcohol
DMSO	Dimethyl sulfoxide
HQNO	2-Heptyl-4-quinolone-*N*-oxide
NQNO	2-Nonyl-4-quinolone-*N*-oxide
