# Supplementary material for: A Synthesis of 4-Quinolone N-Oxides and NMR Evidence of Their Protonation-Assisted Enolisation
Source: Molecules. 2026 May 15;31(10):1680. doi: 10.3390/molecules31101680 (PMC13209533; doi:10.3390/molecules31101680)
Supplement: Supplementary file 1 [file molecules-31-01680-s001.zip › SI.pdf]

A Synthesis of 4-Quinolone *N*-Oxides and NMR Evidence of Their Protonation-assisted Enolisation – Supporting Information

Plamen Angelov, Yordanka Sapundzhieva, Francisco Alonso and Paraskev Nedialkov

Table of contents Page

NMR spectra of 3/3’ (400/100 MHz Bruker Avance NEO 400) S2

NMR spectra of 3/3’’ (80/20 MHz Magritek Spinsolve 80) S21

HPLC traces and HRMS S36

IR Spectra S38

Primary NMR data available at: https://doi.org/10.5281/zenodo.19204221

^{1}H and ^{13}C NMR spectra of 3/3' (400/100 MHz Bruker Avance NEO 400)

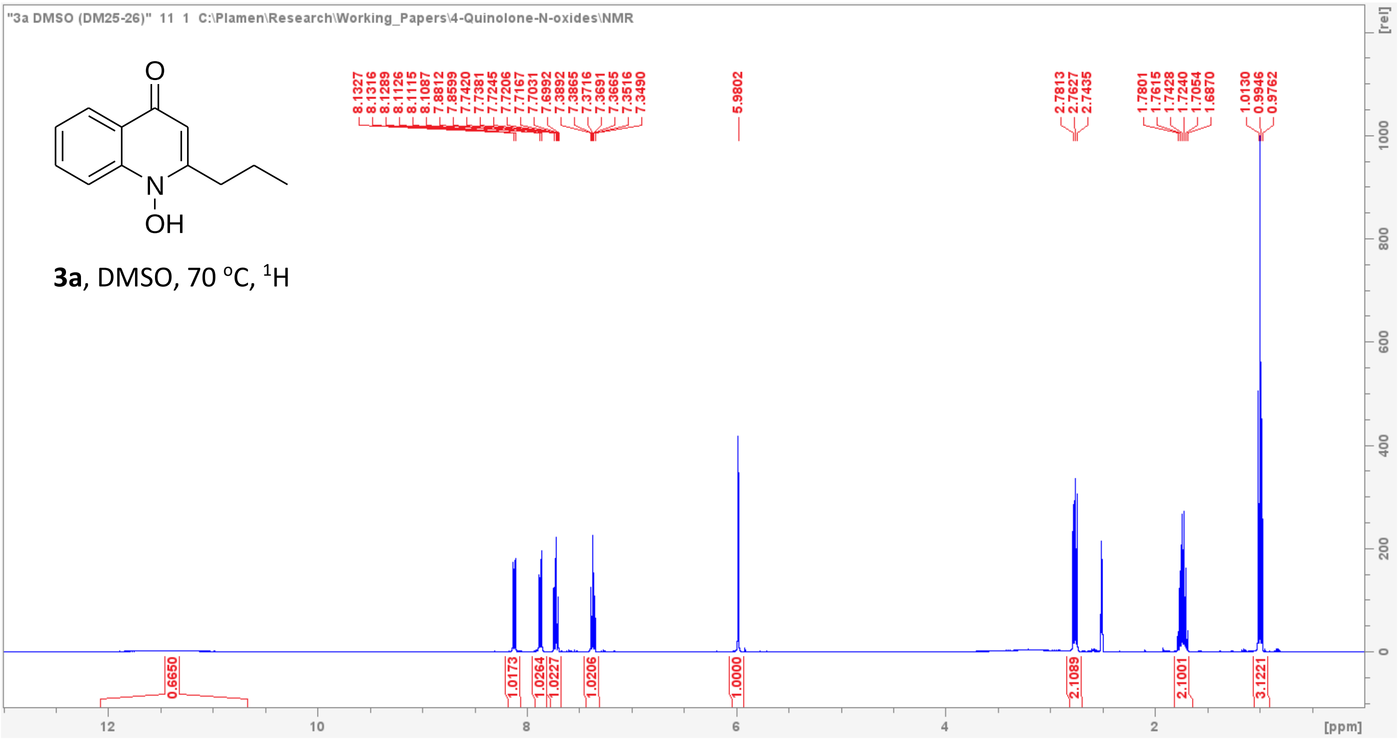

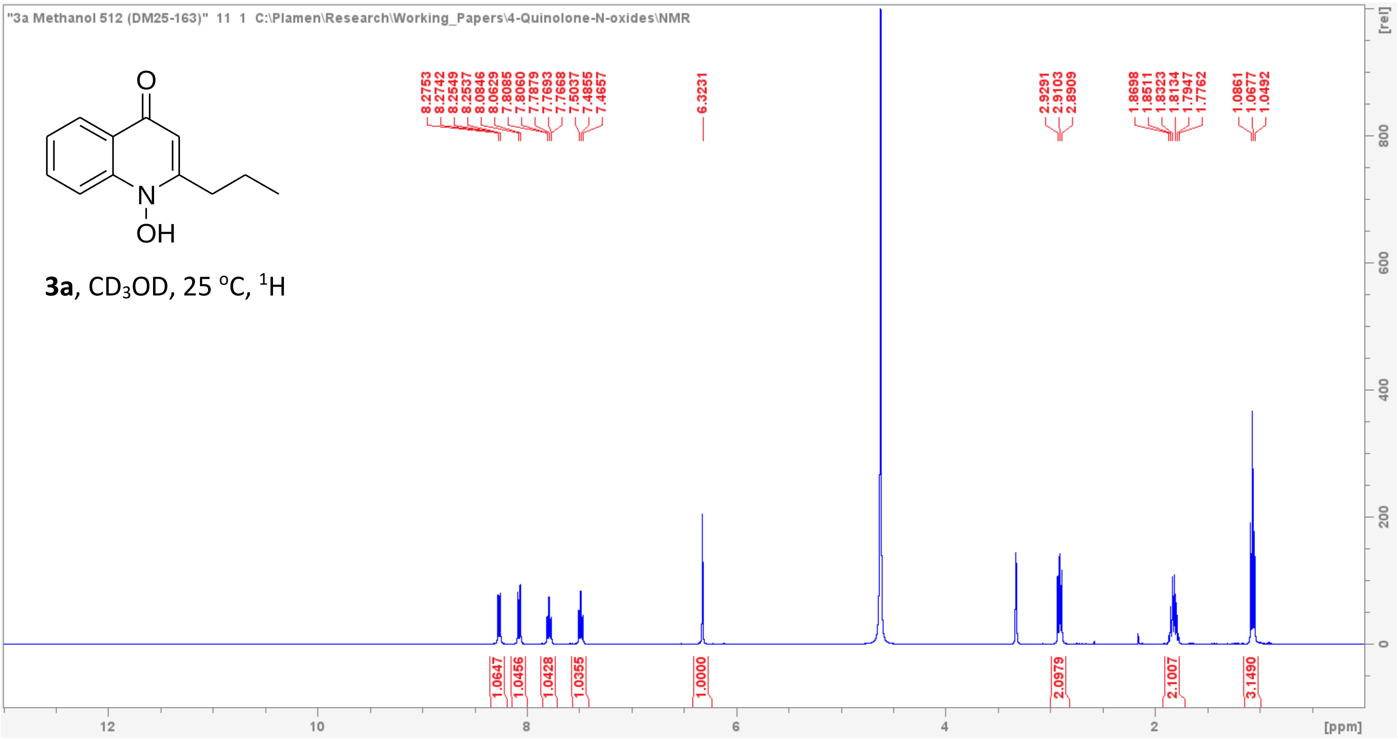

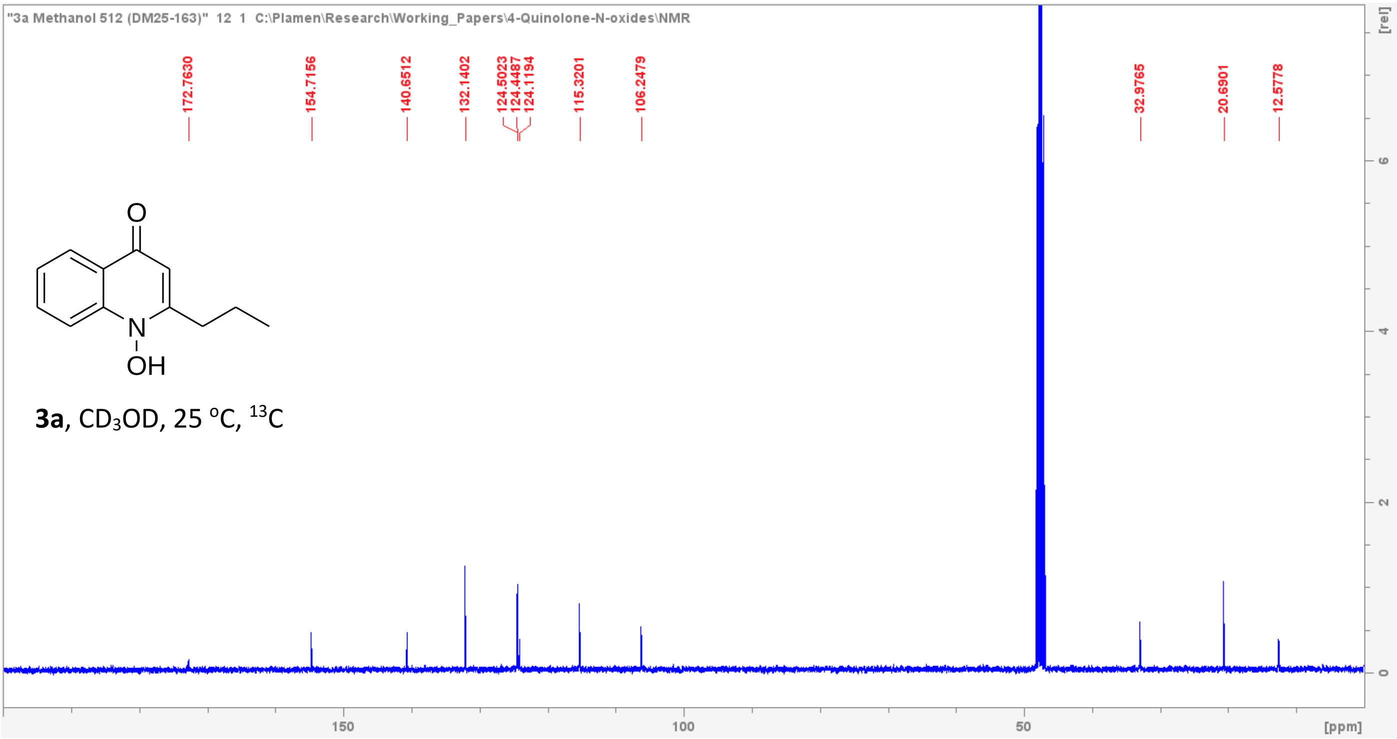
"3a Methanol 512 (DM25-163)" 12 1 C:\Plamen\Research\Working_Papers\4-Quinolone-N-oxides\NMR
172.7630
154.7156
140.6512
132.1402
124.5023
124.4487
124.1194
115.3201
106.2479
32.9765
20.6901
12.5778
O
N
OH
3a, CD_3OD, 25 °C, ^{13}C
[rel]
[ppm]
150
100
50

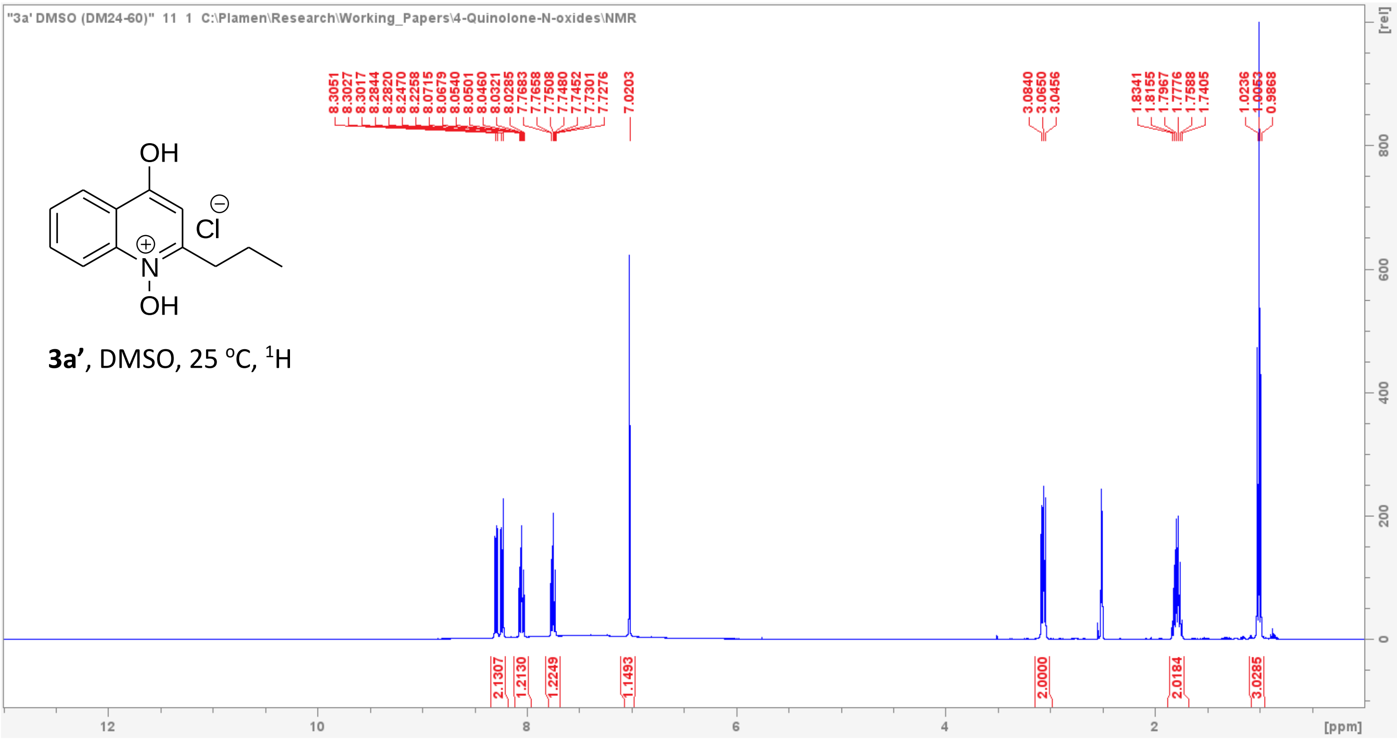
"3a' DMSO (DM24-60)" 11 1 C:\Plamen\Research\Working_Papers\4-Quinolone-N-oxides\NMR
8.3051
8.3027
8.3017
8.2844
8.2820
8.2470
8.2258
8.0715
8.0679
8.0540
8.0501
8.0460
8.0321
8.0285
7.7683
7.7508
7.7480
7.7452
7.7301
7.7276
7.0203
3.0840
3.0650
3.0456
1.8341
1.8155
1.7967
1.7776
1.7588
1.7405
1.0236
1.0053
0.9868
OH
Cl
N
OH
3a', DMSO, 25 °C, ^{1}H
2.1307
1.2130
1.2249
1.1493
2.0000
2.0184
3.0285
[rel]
[ppm]
12
10
8
6
4
2

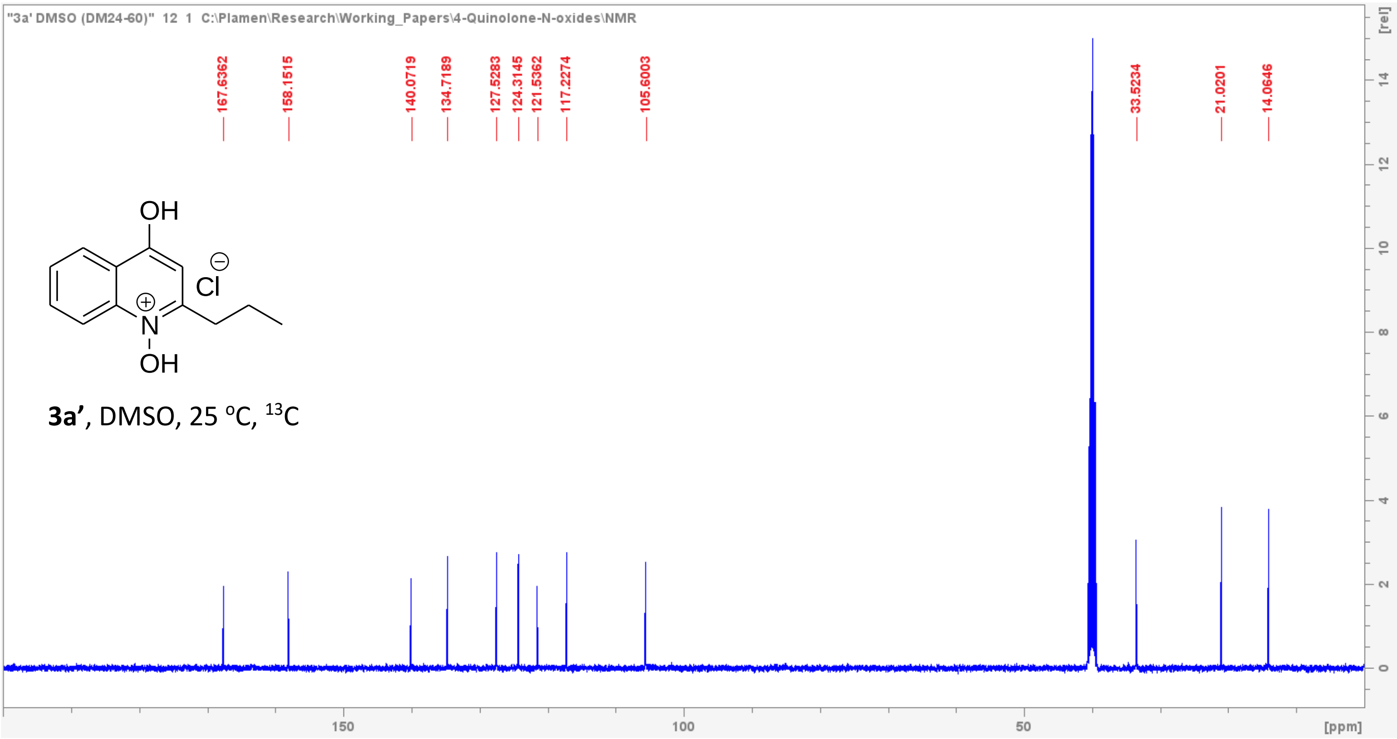
"3a' DMSO (DM24-60)" 12 1 C:\Plamen\Research\Working_Papers\4-Quinolone-N-oxides\NMR
167.6362
158.1515
140.0719
134.7189
127.5283
124.3145
121.5362
117.2274
105.6003
33.5234
21.0201
14.0646
OH
Cl
N
OH
3a', DMSO, 25 °C, 13C
150
100
50
[ppm]
[rel]

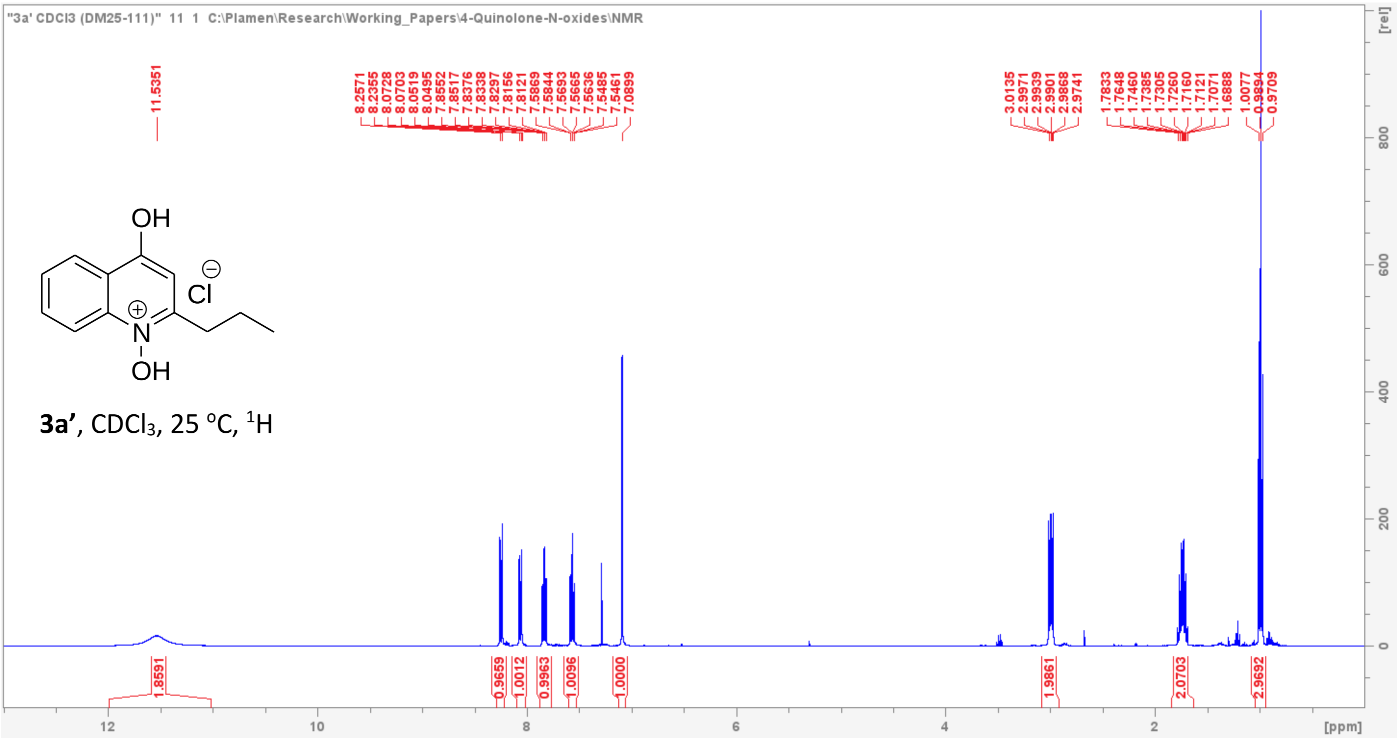
"3a' CDCl3 (DM25-111)" 11 1 C:\Plamen\Research\Working_Papers\4-Quinolone-N-oxides\NMR
11.5351
8.2571
8.2355
8.0728
8.0703
8.0519
8.0495
7.8552
7.8517
7.8376
7.8338
7.8297
7.8156
7.8121
7.5869
7.5844
7.5693
7.5665
7.5636
7.5485
7.5461
7.0899
3.0135
2.9971
2.9939
2.9901
2.9868
2.9741
1.7833
1.7648
1.7460
1.7385
1.7305
1.7260
1.7160
1.7121
1.7071
1.6888
1.0077
0.9894
0.9709
OH
Cl
N
OH
3a', CDCl3, 25 °C, 1H
1.8591
0.9659
1.0012
0.9963
1.0096
1.0000
1.9861
2.0703
2.9692
12
10
8
6
4
2
[ppm]
[rel]

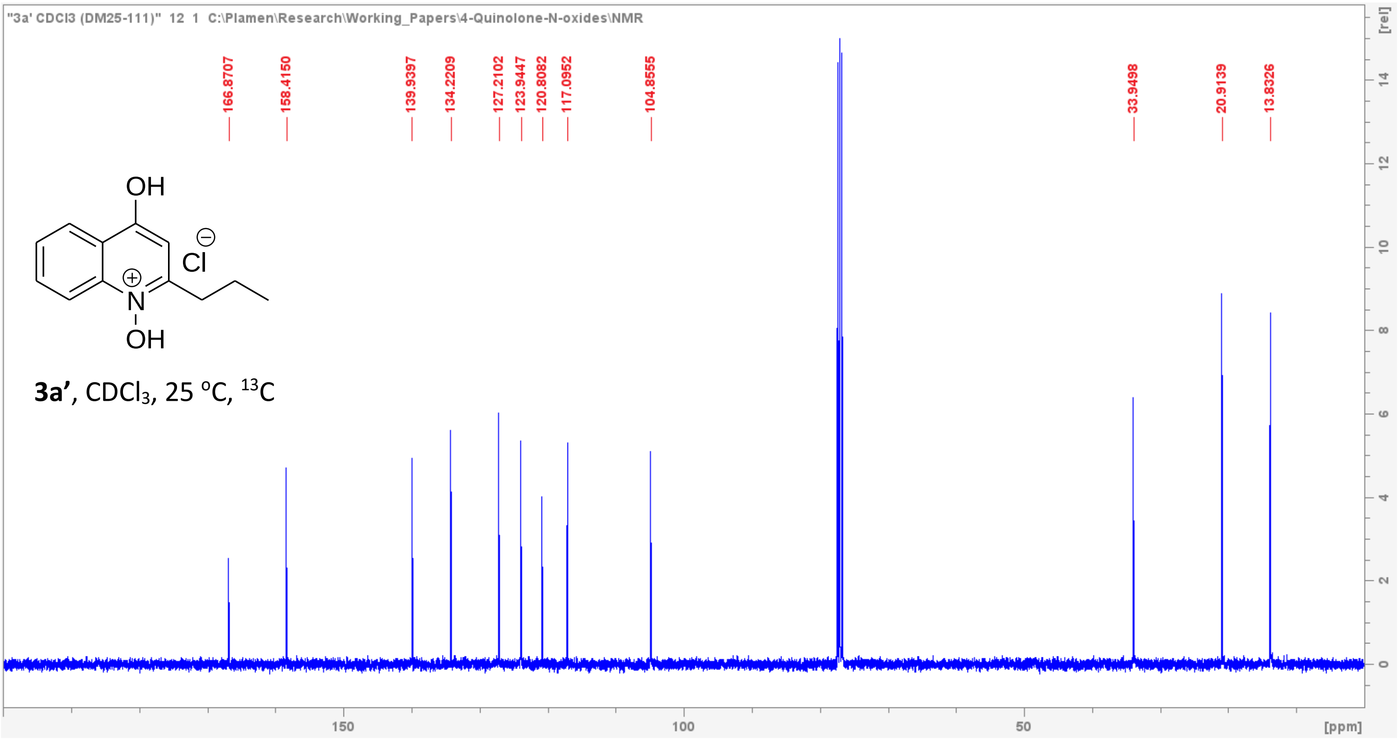
"3a' CDCl3 (DM25-111)" 12 1 C:\Plamen\Research\Working_Papers\4-Quinolone-N-oxides\NMR
166.8707
158.4150
139.9397
134.2209
127.2102
123.9447
120.8082
117.0952
104.8555
33.9498
20.9139
13.8326
OH
Cl
N
OH
3a', CDCl3, 25 °C, 13C
[rel]
[ppm]

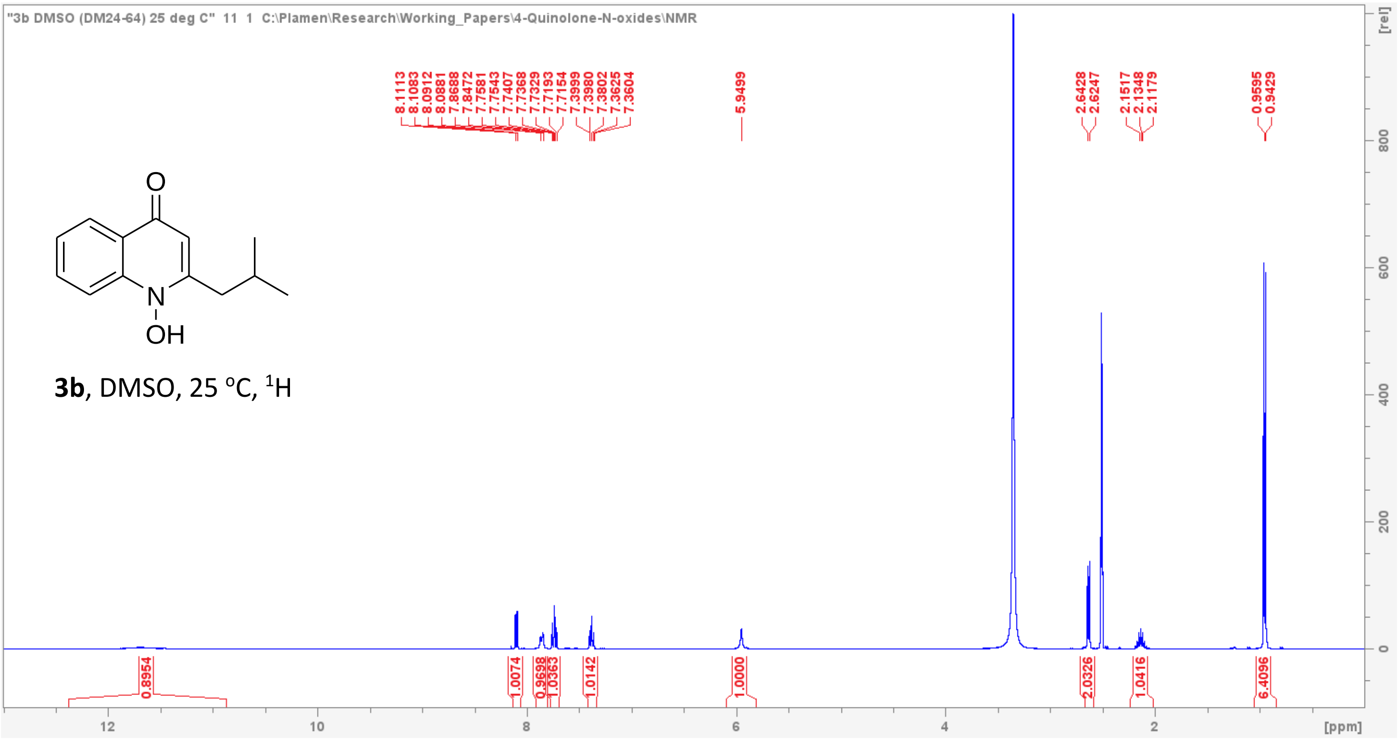
"3b DMSO (DM24-64) 25 deg C" 11 1 C:\Plamen\Research\Working_Papers\4-Quinolone-N-oxides\NMR
8.1113
8.1083
8.0912
8.0881
7.8688
7.8472
7.7581
7.7543
7.7407
7.7368
7.7329
7.7193
7.7154
7.3999
7.3980
7.3802
7.3625
7.3604
5.9499
2.6428
2.6247
2.1517
2.1348
2.1179
0.9595
0.9429
O
N
OH
3b, DMSO, 25 °C, 1H
0.8954
1.0074
0.9698
1.0363
1.0142
1.0000
2.0326
1.0416
6.4096
[rel]
[ppm]

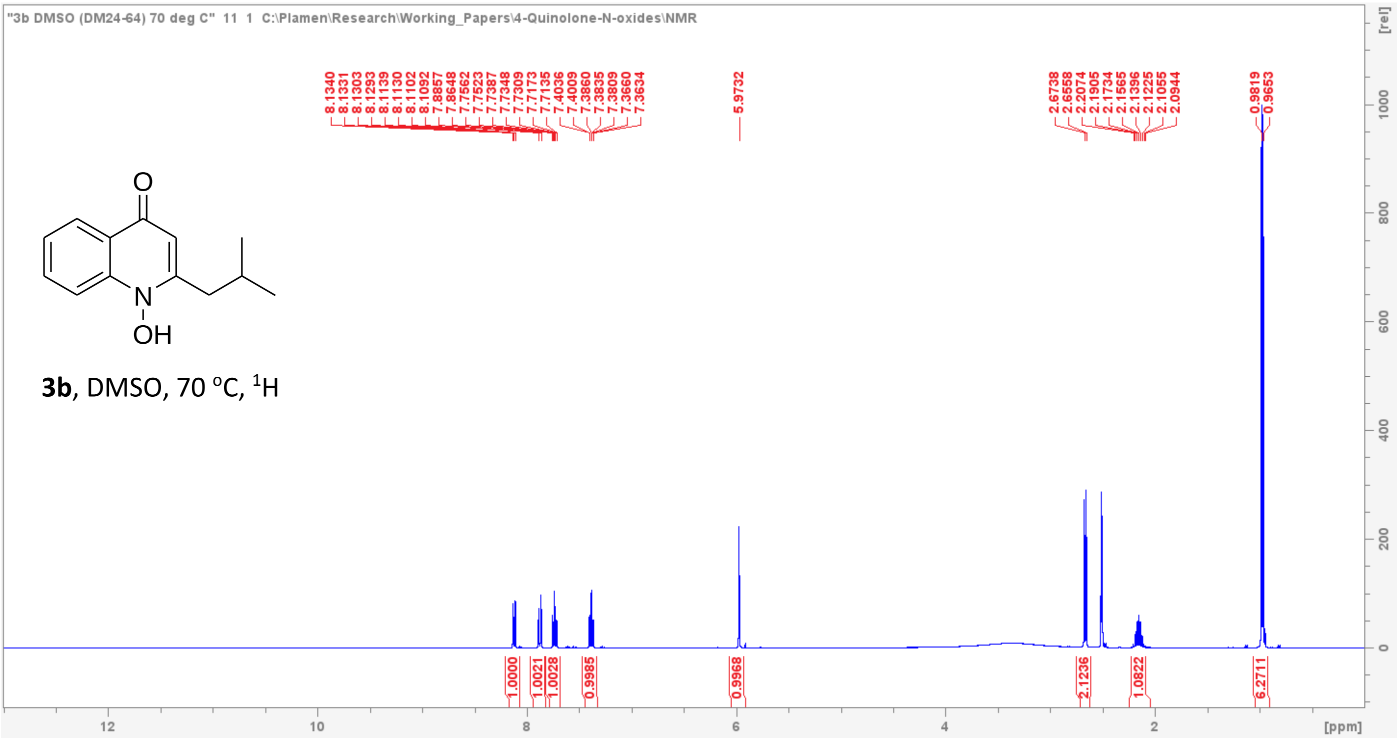
"3b DMSO (DM24-64) 70 deg C" 11 1 C:\Plamen\Research\Working_Papers\4-Quinolone-N-oxides\NMR
8.1340 8.1331 8.1303 8.1293 8.1139 8.1130 8.1102 8.1092 7.8857 7.8648 7.7562 7.7523 7.7387 7.7348 7.7309 7.7173 7.7135 7.4036 7.4009 7.3860 7.3835 7.3809 7.3660 7.3634
5.9732
2.6738 2.6558 2.2074 2.1905 2.1734 2.1565 2.1396 2.1225 2.1055 2.0944
0.9819 0.9653
1.0000 1.0021 1.0028 0.9985 0.9968 2.1236 1.0822 6.2711
O
N
OH
3b, DMSO, 70 °C, 1H
[ppm]
[rel]

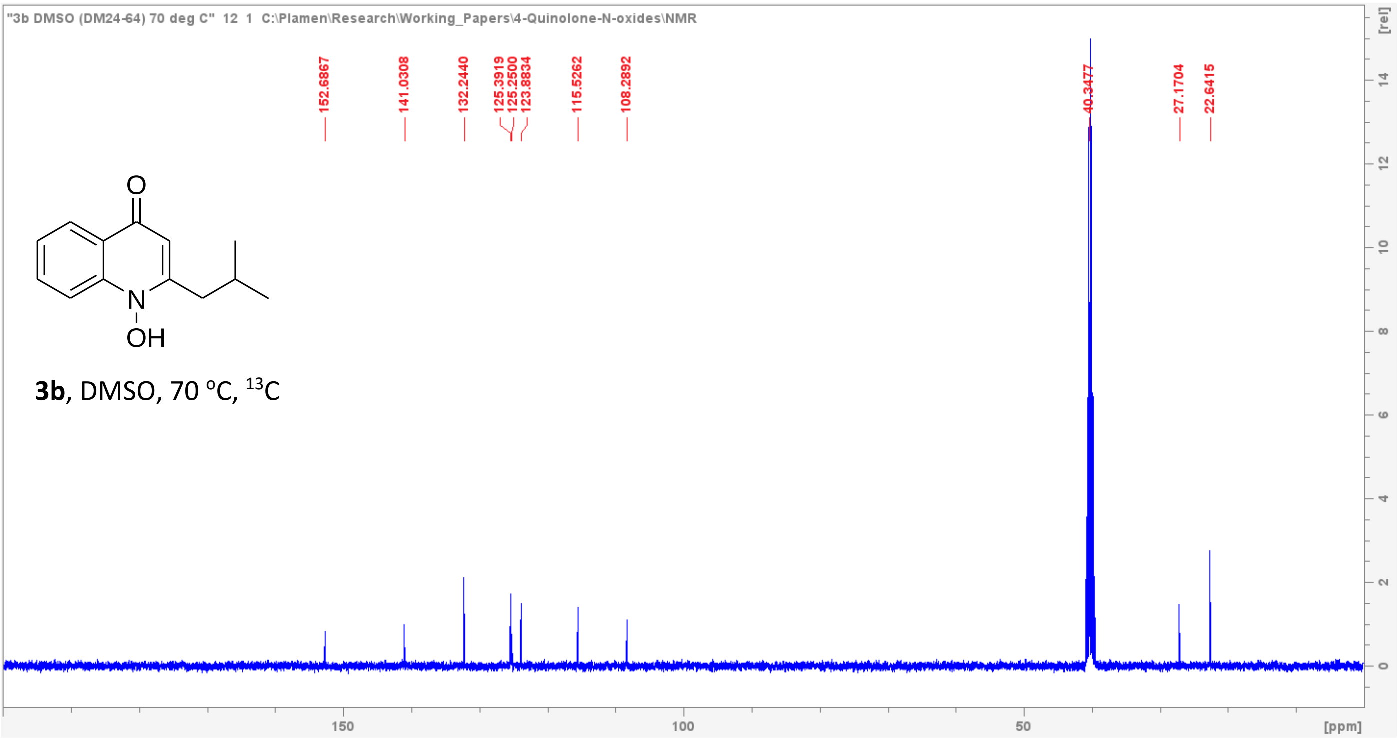
"3b DMSO (DM24-64) 70 deg C" 12 1 C:\Plamen\Research\Working_Papers\4-Quinolone-N-oxides\NMR
152.6867 141.0308 132.2440 125.3919 125.2500 123.8834 115.5262 108.2892
40.3477 27.1704 22.6415
O
N
OH
3b, DMSO, 70 °C, 13C
[ppm]
[rel]

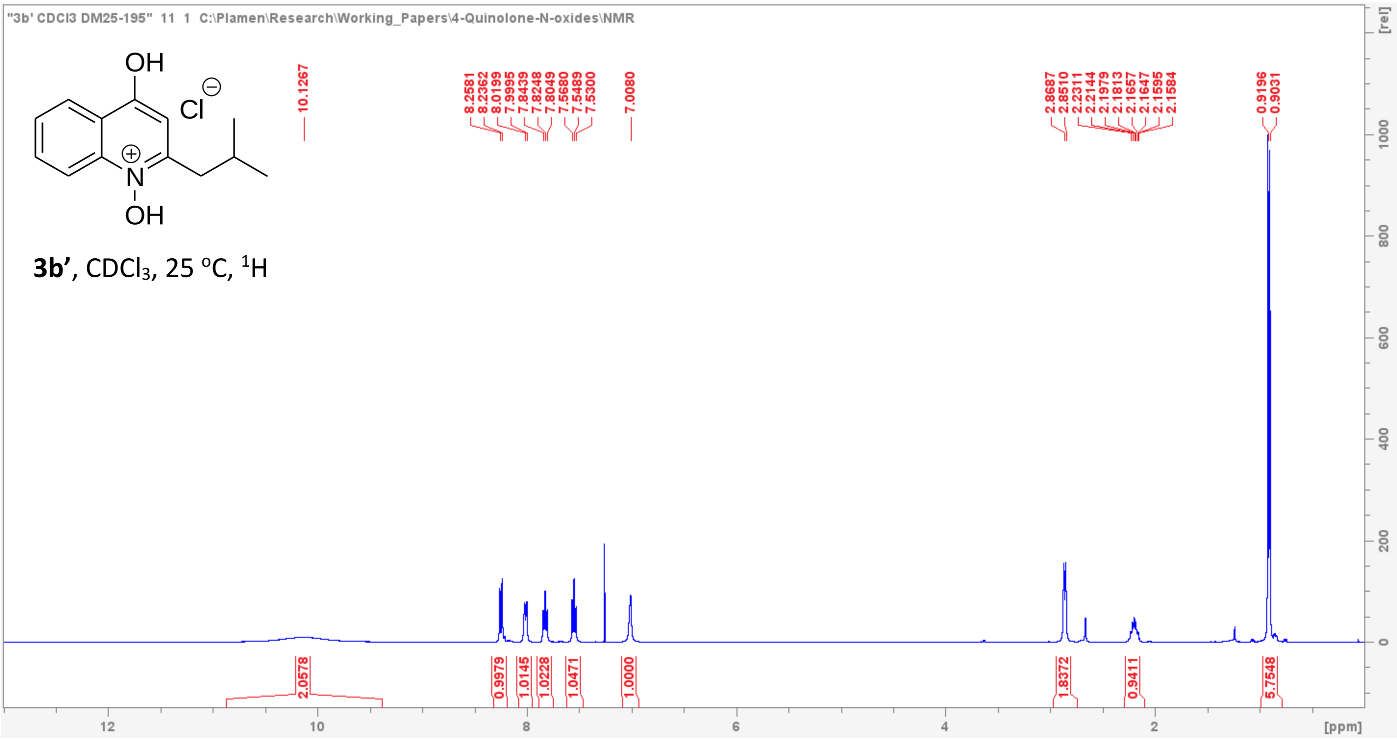
"3b' CDCl3 DM25-195" 11 1 C:\Plamen\Research\Working_Papers\4-Quinolone-N-oxides\NMR
OH
Cl
N
OH
3b', CDCl3, 25 °C, 1H
10.1267
8.2581
8.2362
8.0199
7.9995
7.8439
7.8248
7.8049
7.5680
7.5489
7.5300
7.0080
2.8687
2.8510
2.2311
2.2144
2.1979
2.1813
2.1657
2.1647
2.1595
2.1584
0.9196
0.9031
2.0578
0.9979
1.0145
1.0228
1.0471
1.0000
1.8372
0.9411
5.7548
[ppm]
[rel]

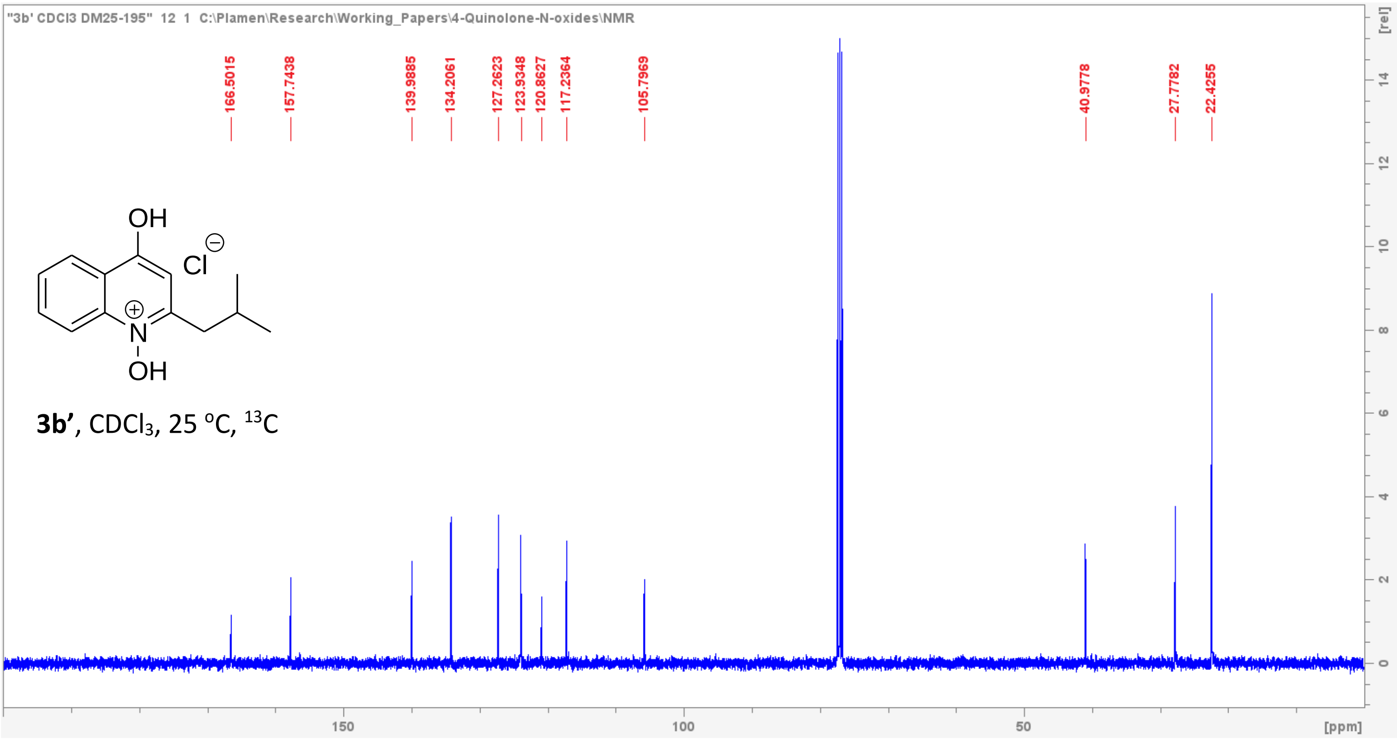
"3b' CDCl3 DM25-195" 12 1 C:\Plamen\Research\Working_Papers\4-Quinolone-N-oxides\NMR
166.5015
157.7438
139.9885
134.2061
127.2623
123.9348
120.8627
117.2364
105.7969
40.9778
27.7782
22.4255
OH
Cl
N
OH
3b', CDCl3, 25 °C, 13C
[ppm]
[rel]

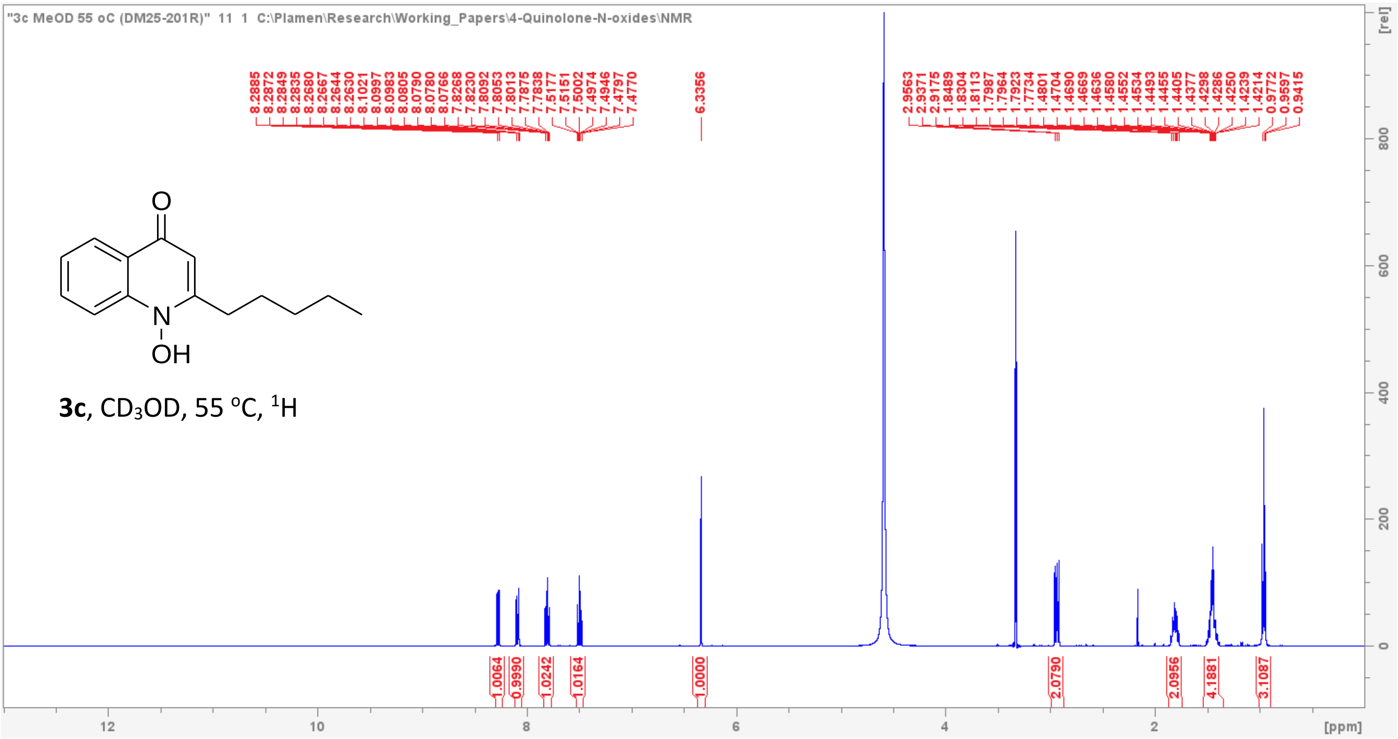
"3c MeOD 55 oC (DM25-201R)" 11 1 C:\Plamen\Research\Working_Papers\4-Quinolone-N-oxides\NMR
8.2885 8.2872 8.2849 8.2835 8.2680 8.2667 8.2644 8.2630 8.1021 8.0997 8.0983 8.0805 8.0790 8.0780 8.0766 7.8268 7.8230 7.8092 7.8053 7.8013 7.7875 7.7838 7.5177 7.5151 7.5002 7.4974 7.4946 7.4797 7.4770
6.3356
2.9563 2.9371 2.9175 1.8489 1.8304 1.8113 1.7987 1.7964 1.7923 1.7734 1.4801 1.4704 1.4690 1.4669 1.4636 1.4580 1.4552 1.4534 1.4493 1.4455 1.4405 1.4377 1.4298 1.4286 1.4250 1.4239 1.4214 0.9772 0.9597 0.9415
O
N
OH
3c, CD3OD, 55 °C, 1H
1.0064 0.9990 1.0242 1.0164 1.0000 2.0790 2.0956 4.1881 3.1087
12 10 8 6 4 2 [ppm]
[rel] 0 200 400 600 800

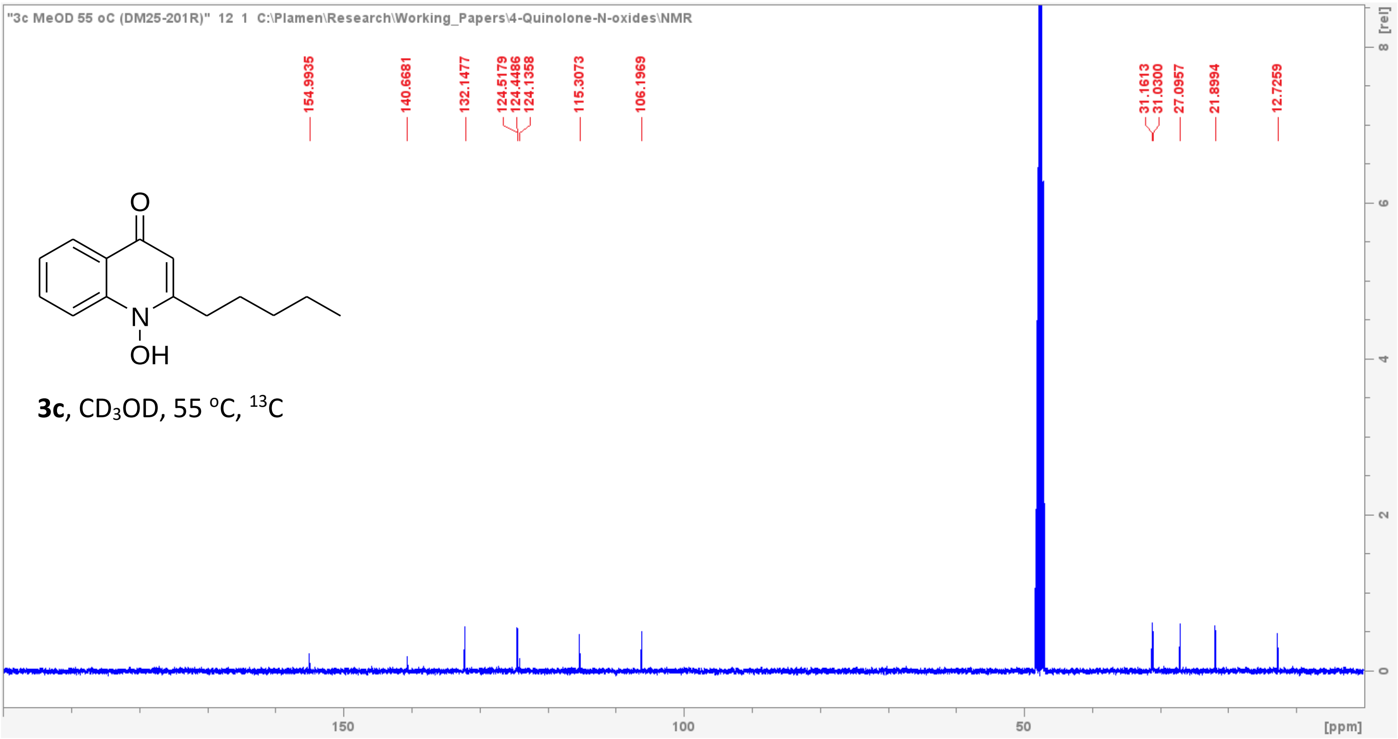
"3c MeOD 55 oC (DM25-201R)" 12 1 C:\Plamen\Research\Working_Papers\4-Quinolone-N-oxides\NMR
154.9935 140.6681 132.1477 124.5179 124.4486 124.1358 115.3073 106.1969
31.1613 31.0300 27.0957 21.8994 12.7259
O
N
OH
3c, CD3OD, 55 °C, 13C
150 100 50 [ppm]
[rel] 0 2 4 6 8

3c, CD_3OD, HMBC

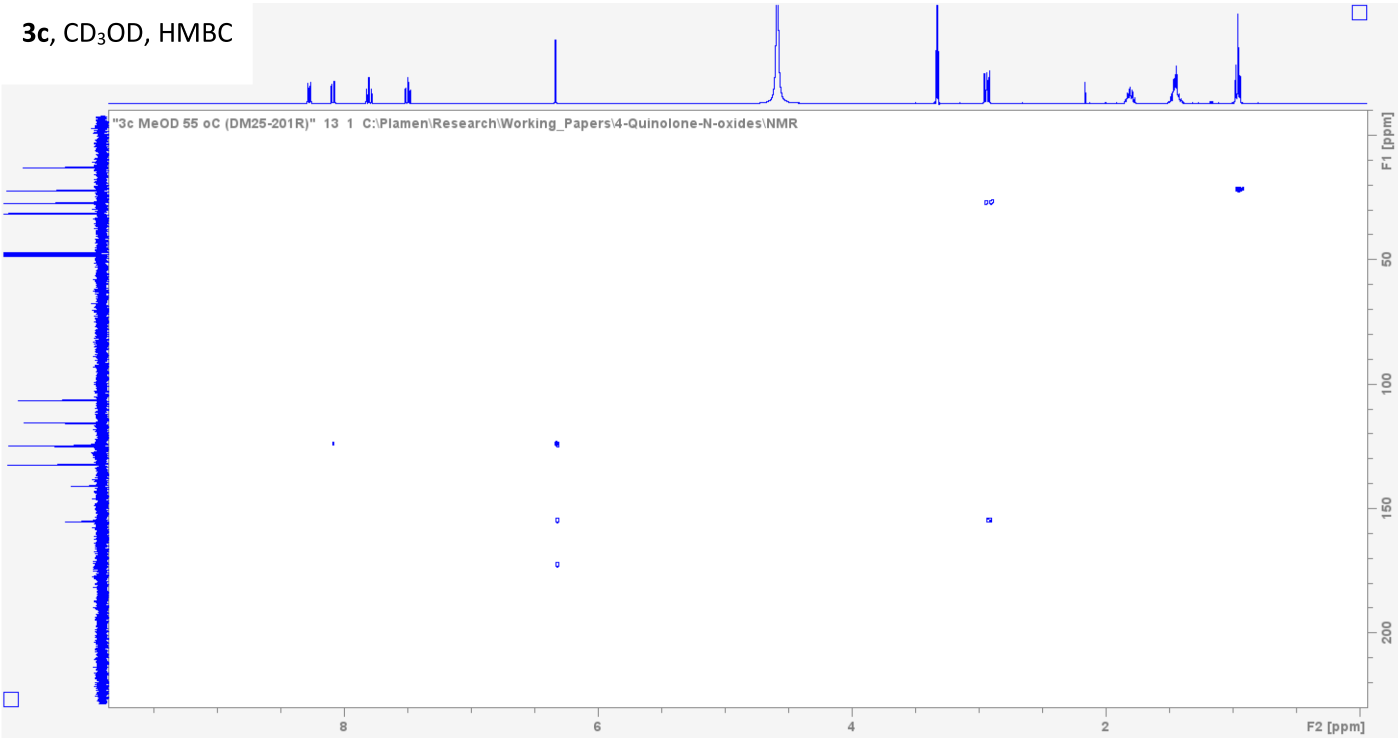

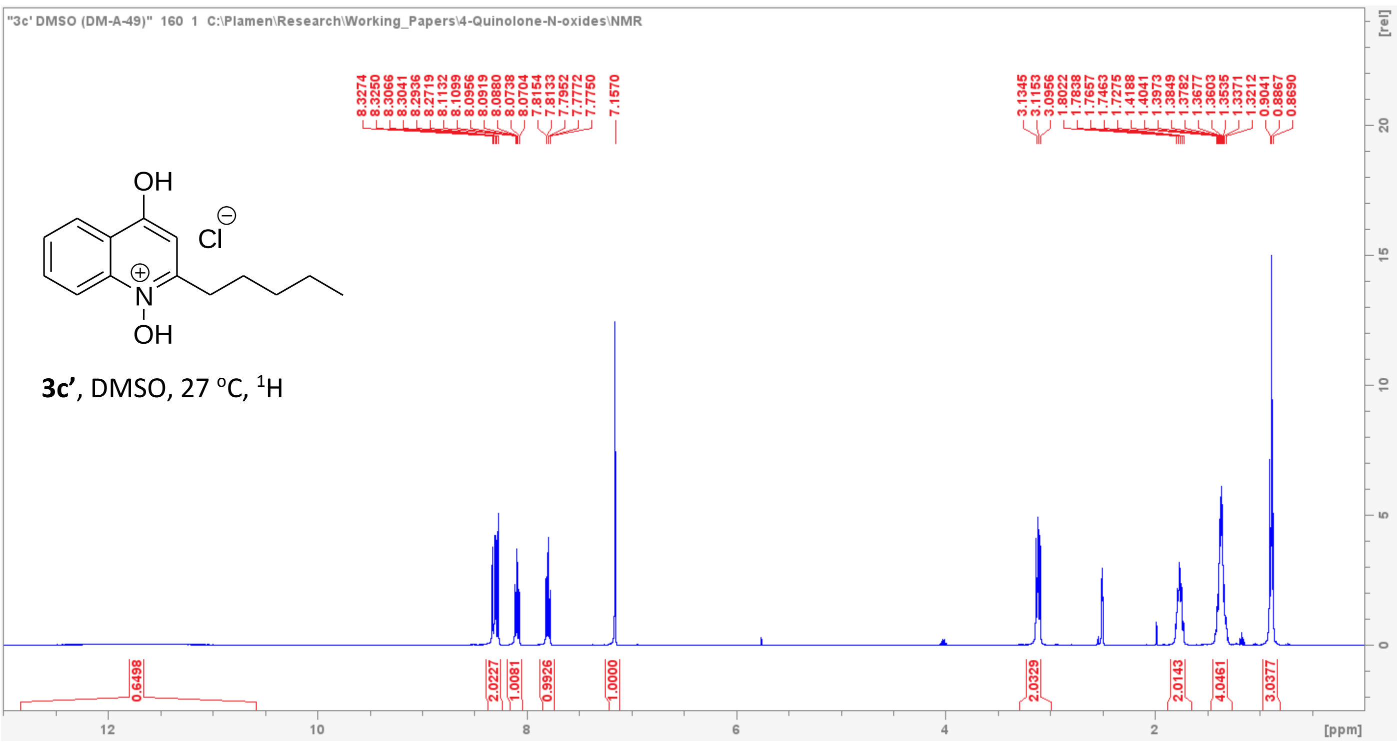

3c', DMSO, 27 °C, [1]H

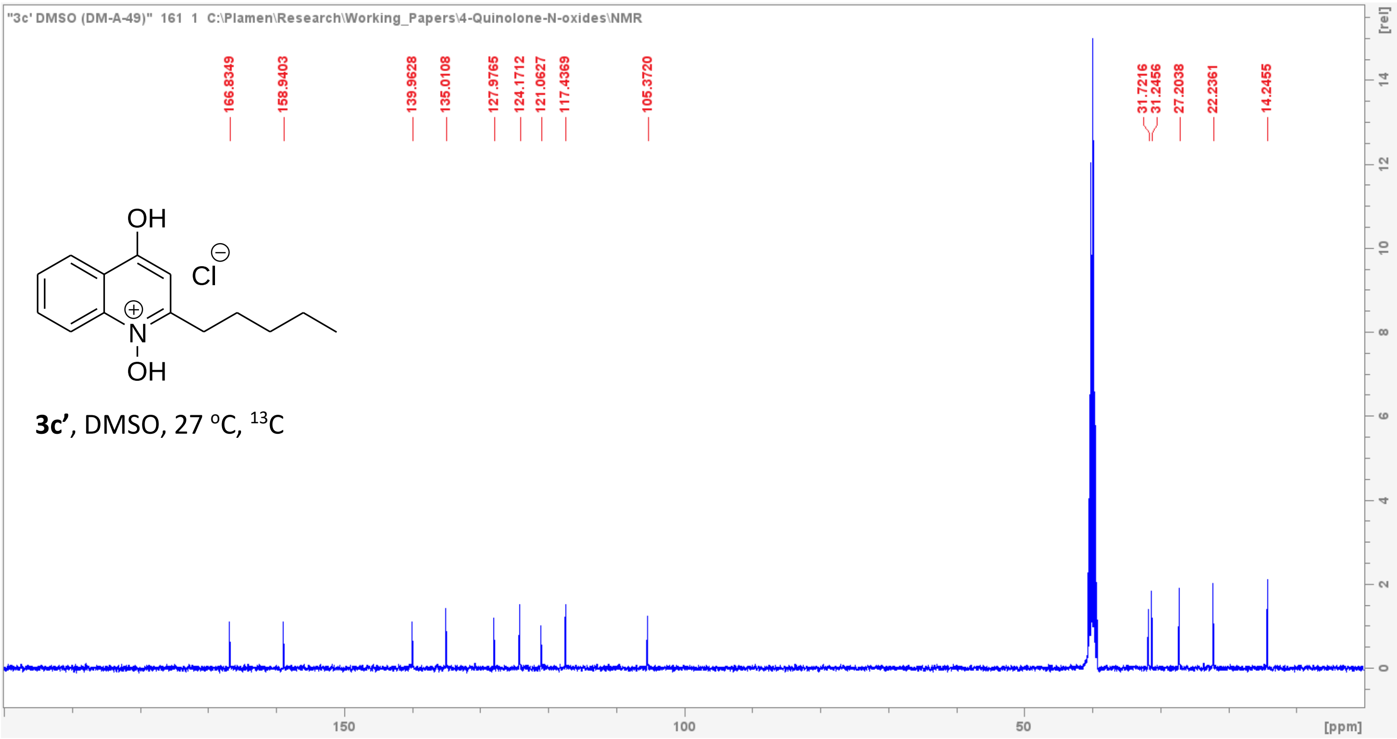
"3c' DMSO (DM-A-49)" 161 1 C:\Plamen\Research\Working_Papers\4-Quinolone-N-oxides\NMR
166.8349
158.9403
139.9628
135.0108
127.9765
124.1712
121.0627
117.4369
105.3720
31.7216
31.2456
27.2038
22.2361
14.2455
OH
Cl
N
OH
3c', DMSO, 27 °C, 13C
[rel]
150
100
50
[ppm]

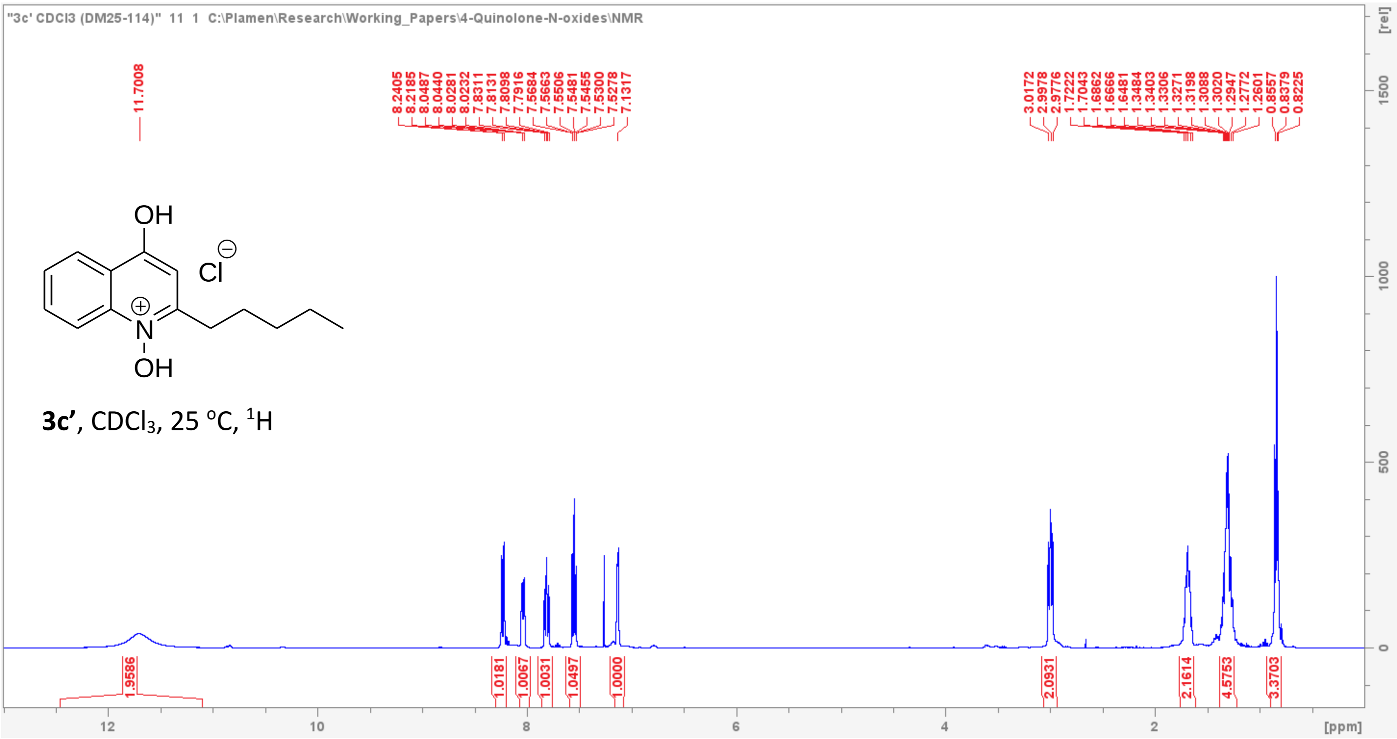
"3c' CDCl3 (DM25-114)" 11 1 C:\Plamen\Research\Working_Papers\4-Quinolone-N-oxides\NMR
11.7008
8.2405
8.2185
8.0487
8.0440
8.0281
8.0232
7.8311
7.8131
7.8098
7.7916
7.5684
7.5663
7.5506
7.5481
7.5455
7.5300
7.5278
7.1317
3.0172
2.9978
2.9776
1.7222
1.7043
1.6862
1.6666
1.6481
1.3484
1.3403
1.3306
1.3271
1.3198
1.3088
1.3020
1.2947
1.2772
1.2601
0.8557
0.8379
0.8225
OH
Cl
N
OH
3c', CDCl3, 25 °C, 1H
1.9586
1.0181
1.0067
1.0031
1.0497
1.0000
2.0931
2.1614
4.5753
3.3703
[rel]
1500
1000
500
12
10
8
6
4
2
[ppm]

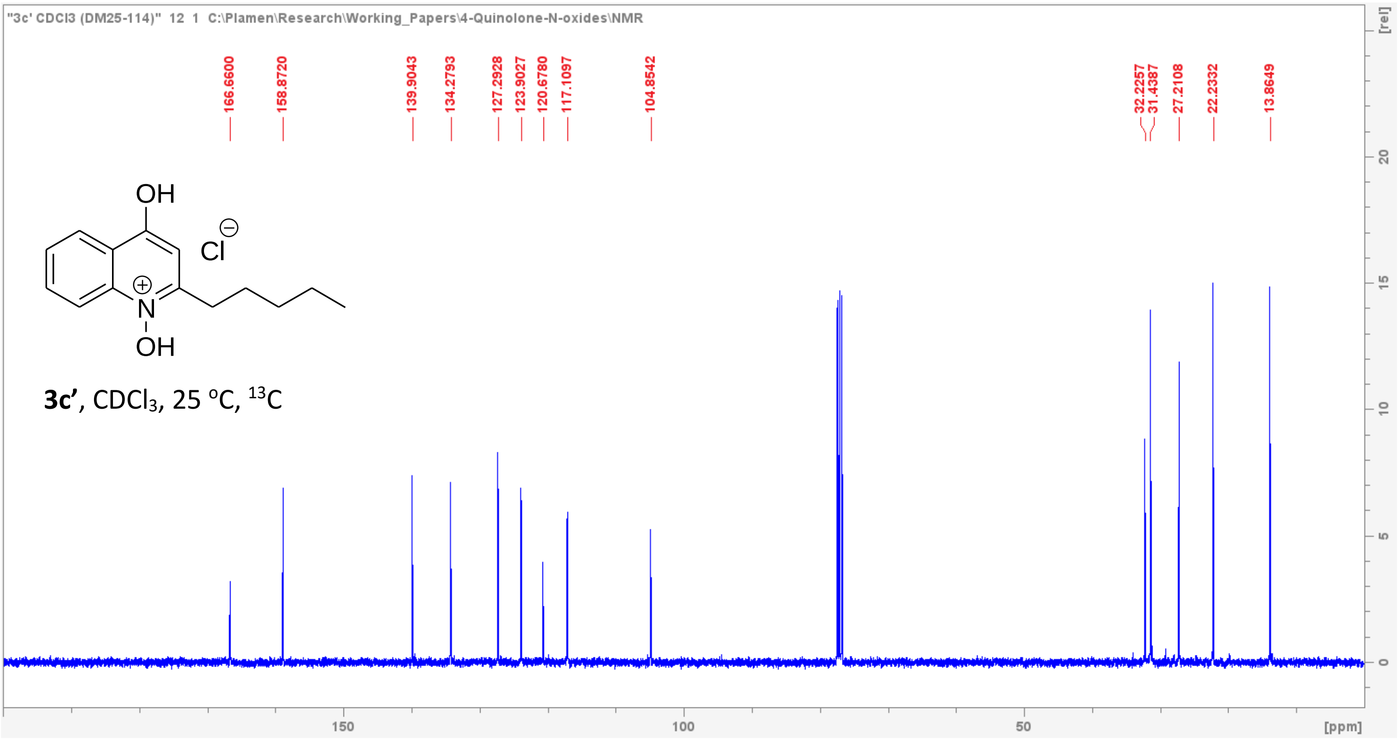
"3c' CDCl3 (DM25-114)" 12 1 C:\Plamen\Research\Working_Papers\4-Quinolone-N-oxides\NMR
166.6600
158.8720
139.9043
134.2793
127.2928
123.9027
120.6780
117.1097
104.8542
32.2257
31.4387
27.2108
22.2332
13.8649
OH
Cl
N
OH
3c', CDCl3, 25 °C, 13C
150
100
50
[ppm]
[rel]

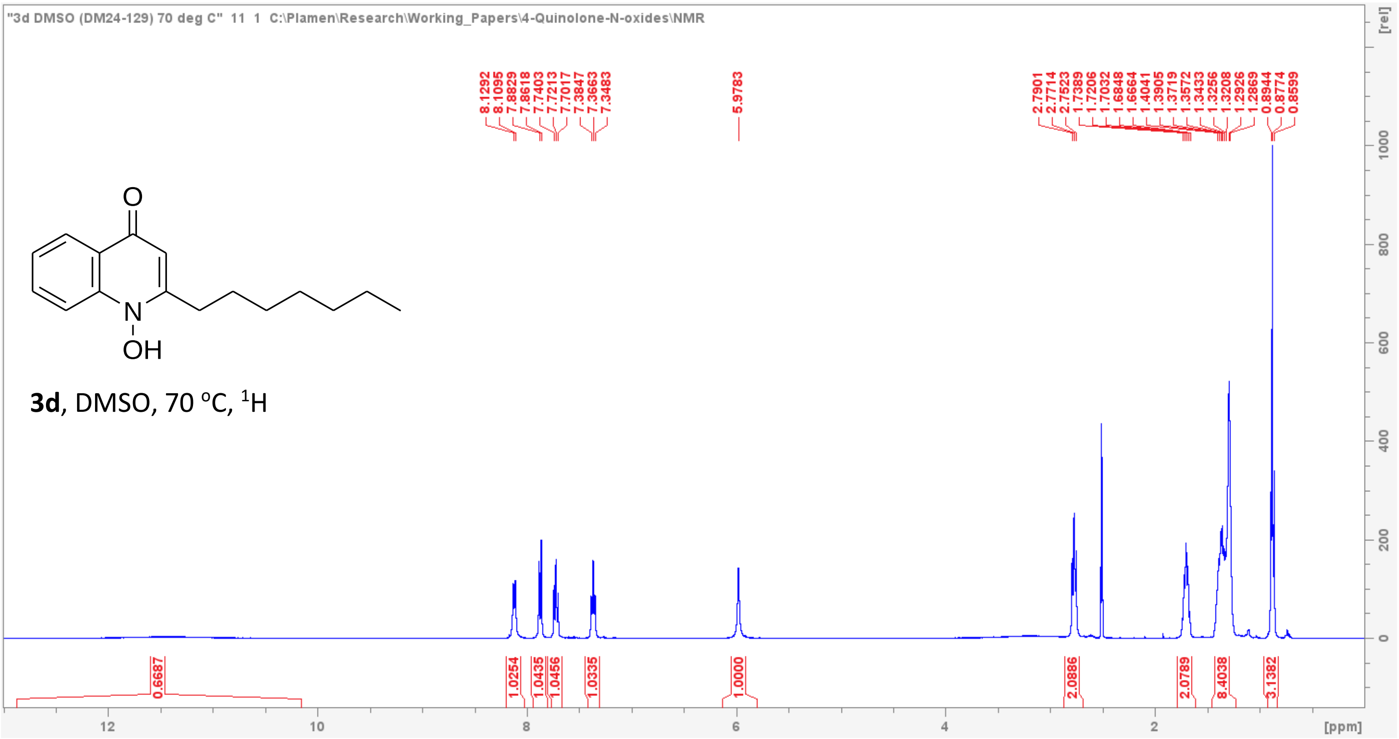
"3d DMSO (DM24-129) 70 deg C" 11 1 C:\Plamen\Research\Working_Papers\4-Quinolone-N-oxides\NMR
8.1292
8.1095
7.8829
7.8618
7.7403
7.7213
7.7017
7.3847
7.3663
7.3483
5.9783
2.7901
2.7714
2.7523
1.7389
1.7206
1.7032
1.6848
1.6664
1.4041
1.3905
1.3719
1.3572
1.3433
1.3256
1.3208
1.2926
1.2869
0.8944
0.8774
0.8599
O
N
OH
3d, DMSO, 70 °C, 1H
0.6687
1.0254
1.0435
1.0456
1.0335
1.0000
2.0886
2.0789
8.4038
3.1382
12
10
8
6
4
2
[ppm]
[rel]

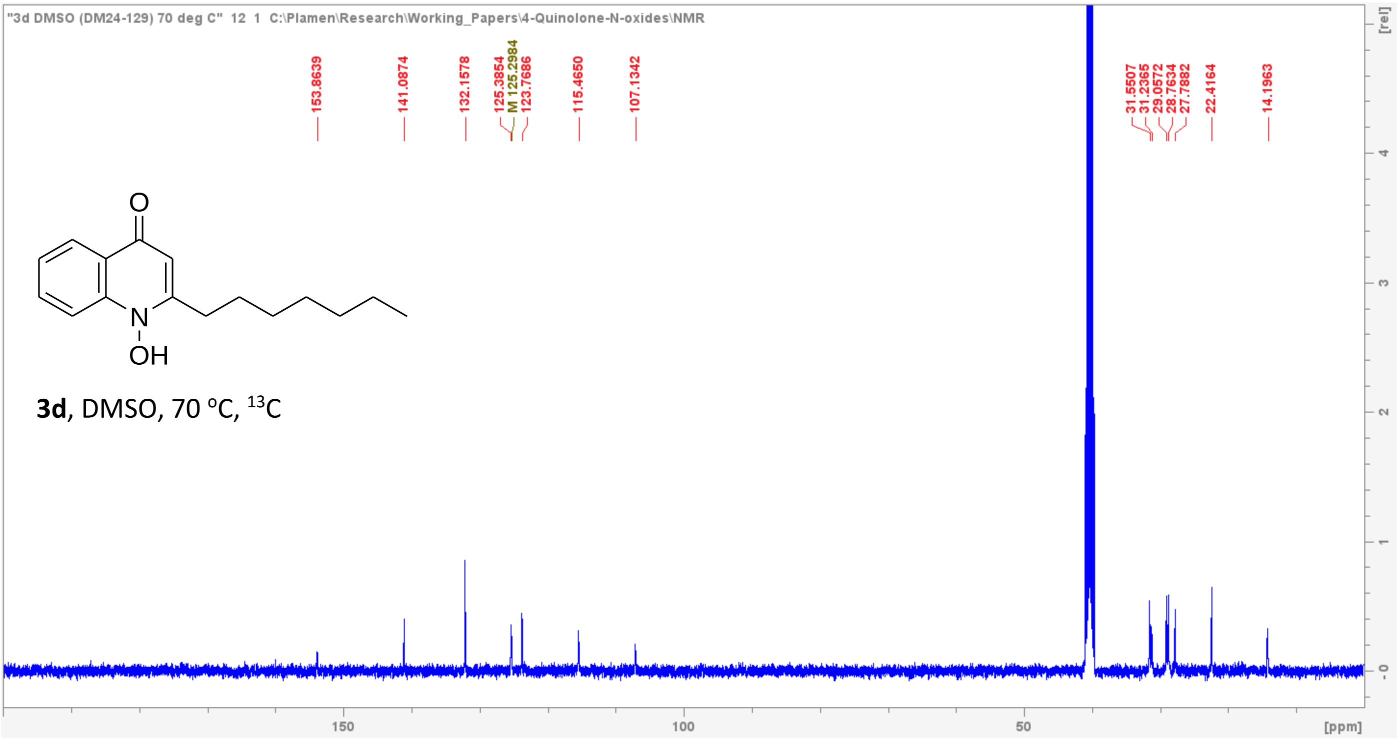
"3d DMSO (DM24-129) 70 deg C" 12 1 C:\Plamen\Research\Working_Papers\4-Quinolone-N-oxides\NMR
153.8639
141.0874
132.1578
125.3854
M 125.2984
123.7686
115.4650
107.1342
31.5507
31.2365
29.0572
28.7634
27.7882
22.4164
14.1963
O
N
OH
3d, DMSO, 70 °C, 13C
150
100
50
[ppm]
[rel]

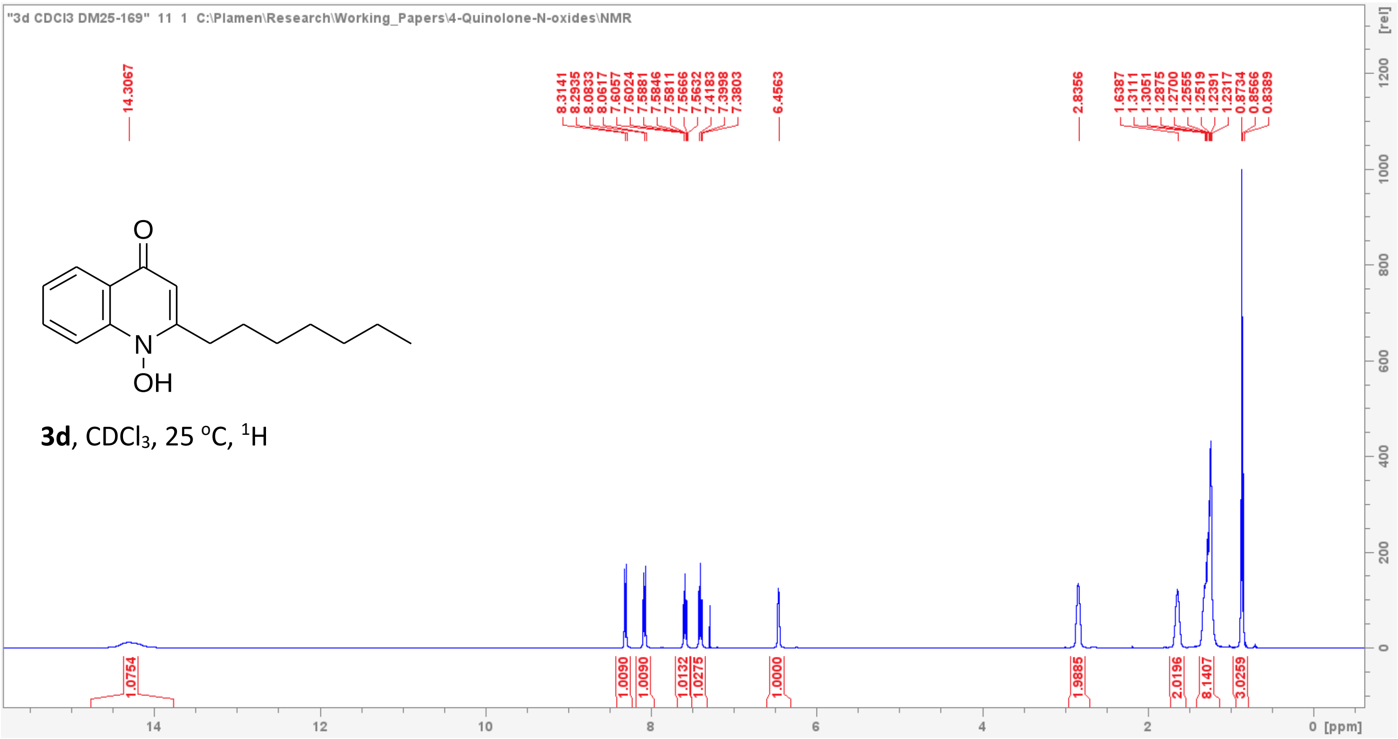
"3d CDCl3 DM25-169" 11 1 C:\Plamen\Research\Working_Papers\4-Quinolone-N-oxides\NMR
14.3067
8.3141
8.2935
8.0833
8.0617
7.6057
7.6024
7.5881
7.5846
7.5811
7.5666
7.5632
7.4183
7.3998
7.3803
6.4563
2.8356
1.6387
1.3111
1.3051
1.2875
1.2700
1.2555
1.2519
1.2391
1.2317
0.8734
0.8566
0.8389
O
N
OH
3d, CDCl3, 25 °C, 1H
1.0754
1.0090
1.0090
1.0132
1.0275
1.0000
1.9885
2.0196
8.1407
3.0259
14
12
10
8
6
4
2
0 [ppm]
[rel]

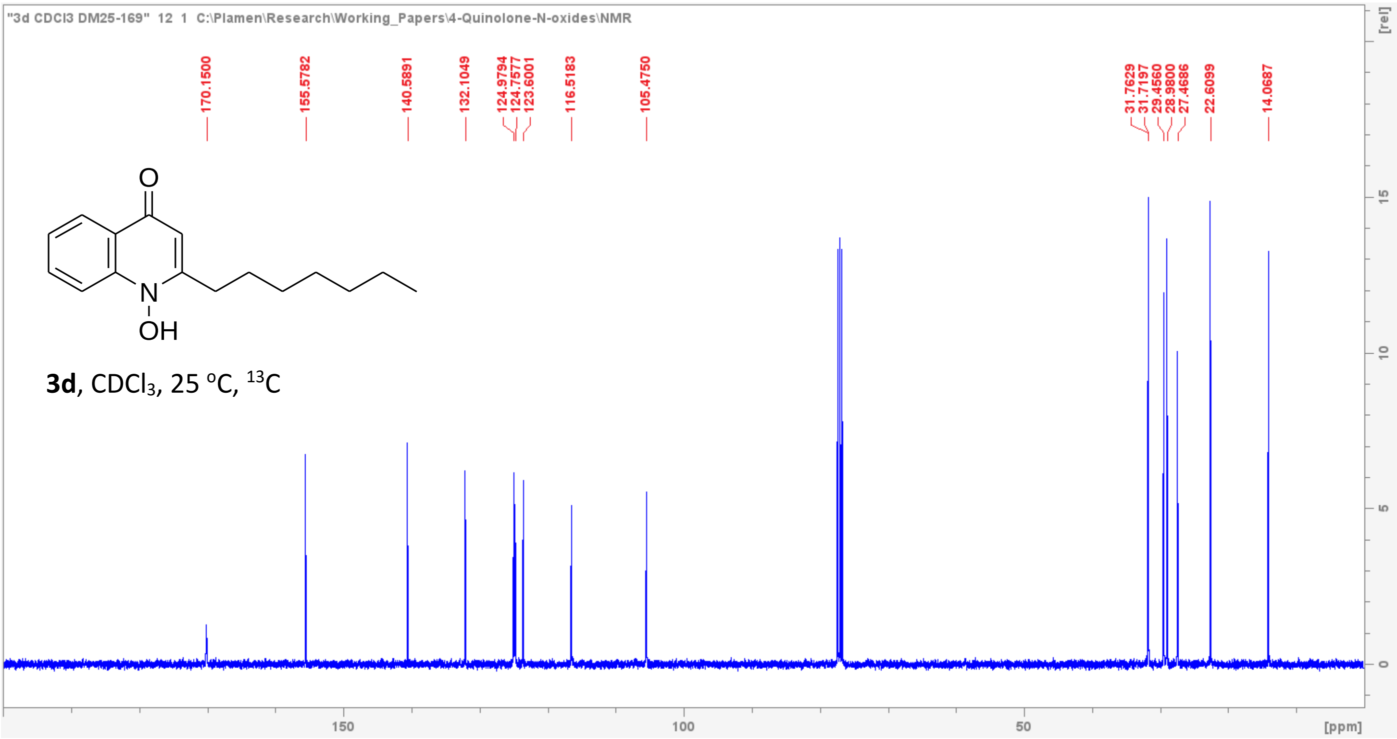
"3d CDCl3 DM25-169" 12 1 C:\Plamen\Research\Working_Papers\4-Quinolone-N-oxides\NMR
170.1500
155.5782
140.5891
132.1049
124.9794
124.7577
123.6001
116.5183
105.4750
31.7629
31.7197
29.4560
28.9800
27.4686
22.6099
14.0687
O
N
OH
3d, CDCl3, 25 °C, 13C
[rel]
[ppm]

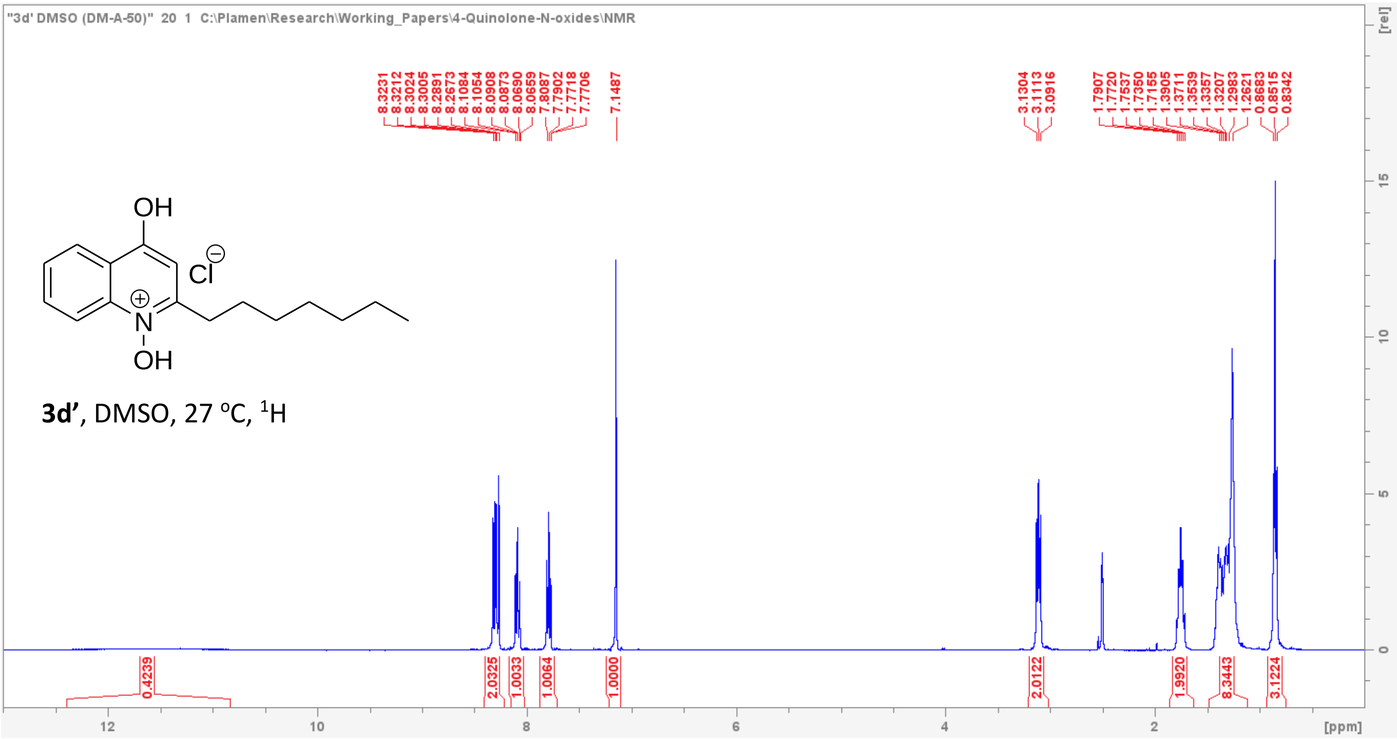
"3d' DMSO (DM-A-50)" 20 1 C:\Plamen\Research\Working_Papers\4-Quinolone-N-oxides\NMR
8.3231
8.3212
8.3024
8.3005
8.2891
8.2673
8.1084
8.1054
8.0908
8.0873
8.0690
8.0659
7.8087
7.7902
7.7718
7.7706
7.1487
3.1304
3.1113
3.0916
1.7907
1.7720
1.7537
1.7350
1.7155
1.3905
1.3711
1.3539
1.3357
1.3207
1.2983
1.2821
0.8683
0.8515
0.8342
OH
Cl
N
OH
3d', DMSO, 27 °C, 1H
0.4239
2.0325
1.0033
1.0064
1.0000
2.0122
1.9920
8.3443
3.1224
[rel]
[ppm]

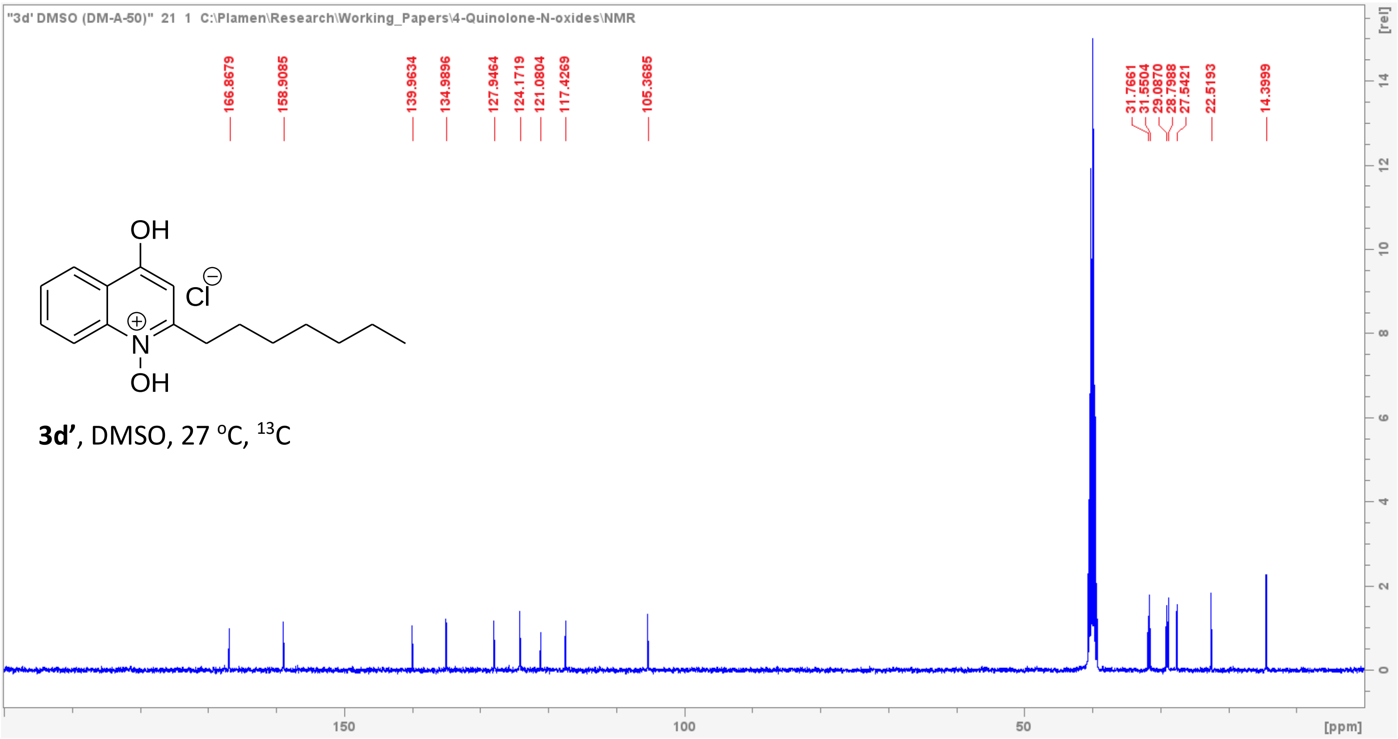
"3d' DMSO (DM-A-50)" 21 1 C:\Plamen\Research\Working_Papers\4-Quinolone-N-oxides\NMR
166.8679
158.9085
139.9634
134.9896
127.9464
124.1719
121.0804
117.4269
105.3685
31.7661
31.5504
29.0870
28.7988
27.5421
22.5193
14.3999
OH
Cl
N
OH
3d', DMSO, 27 °C, 13C
[ppm]
[rel]

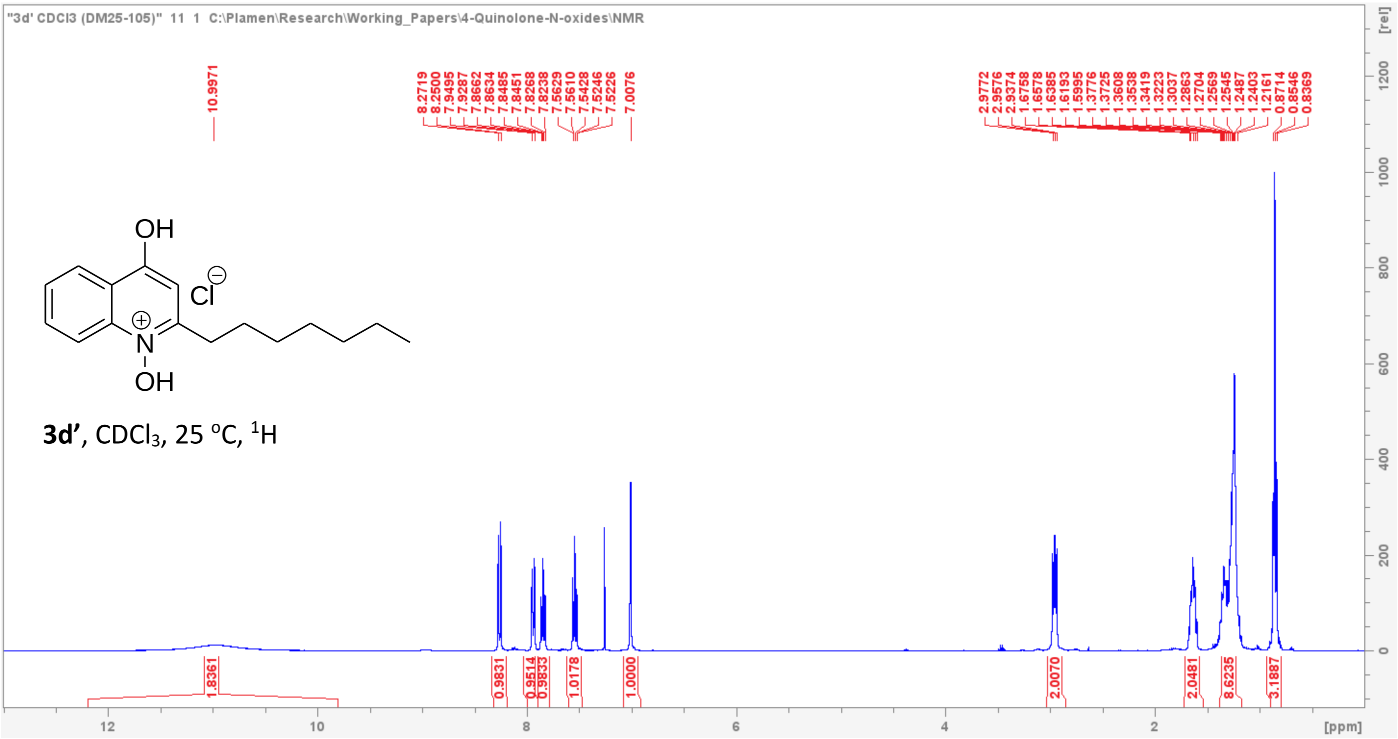
"3d' CDCl3 (DM25-105)" 11 1 C:\Plamen\Research\Working_Papers\4-Quinolone-N-oxides\NMR
OH
Cl
N
OH
3d', CDCl3, 25 °C, 1H
[ppm]
[rel]

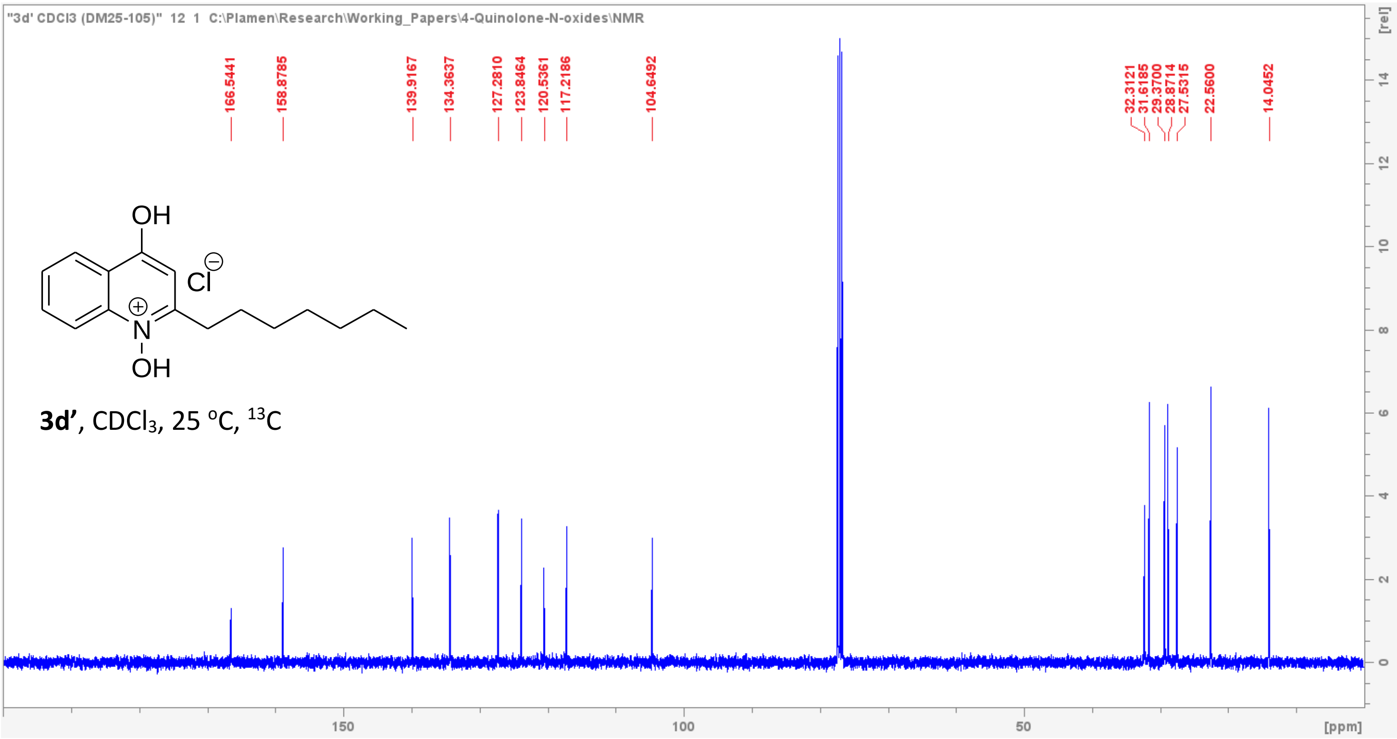
"3d' CDCl3 (DM25-105)" 12 1 C:\Plamen\Research\Working_Papers\4-Quinolone-N-oxides\NMR
166.5441
158.8785
139.9167
134.3637
127.2810
123.8464
120.5361
117.2186
104.6492
32.3121
31.6185
29.3700
28.8714
27.5315
22.5600
14.0452
OH
Cl
N
OH
3d', CDCl3, 25 °C, 13C
[ppm]
[rel]

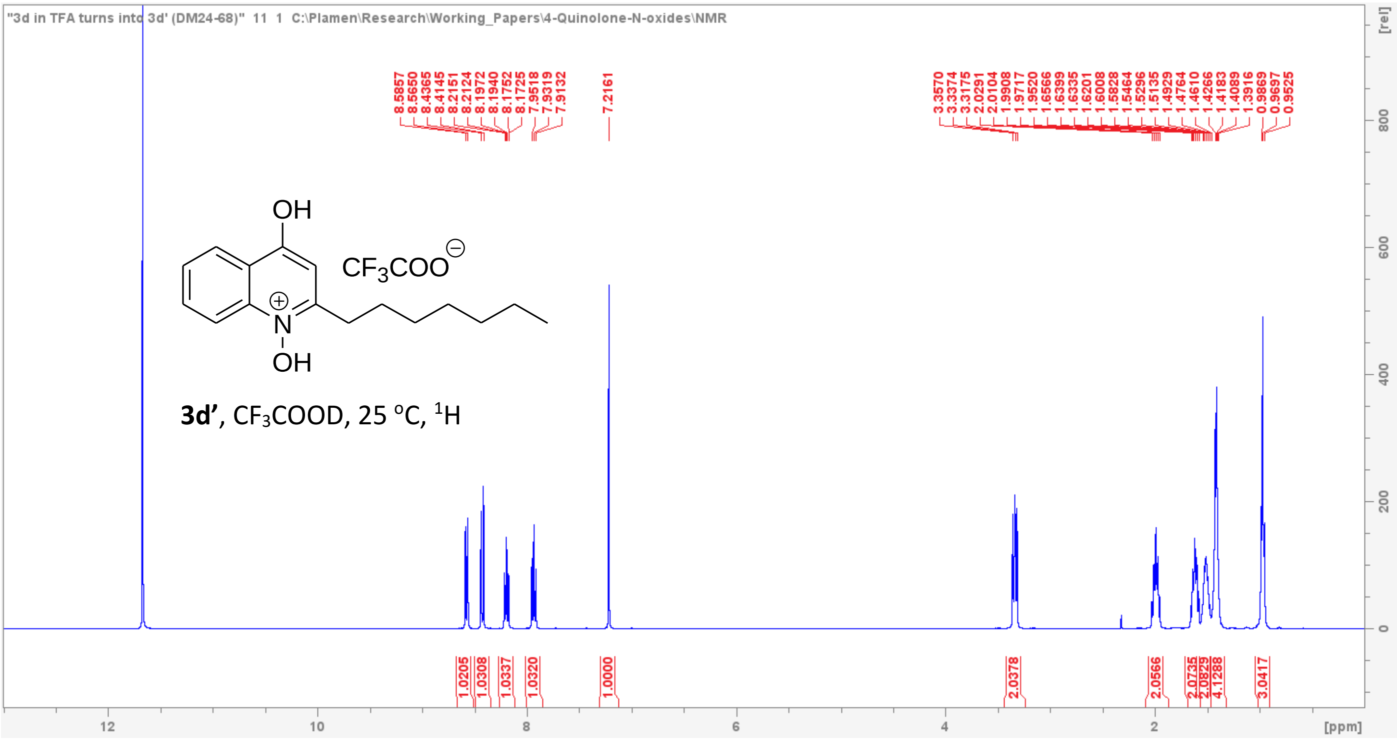
"3d in TFA turns into 3d' (DM24-68)" 11 1 C:\Plamen\Research\Working_Papers\4-Quinolone-N-oxides\NMR
8.5857
8.5650
8.4365
8.4145
8.2151
8.2124
8.1972
8.1940
8.1752
8.1725
7.9518
7.9319
7.9132
7.2161
3.3570
3.3374
3.3175
2.0291
2.0104
1.9908
1.9717
1.9520
1.6566
1.6399
1.6335
1.6201
1.6008
1.5828
1.5464
1.5296
1.5135
1.4929
1.4764
1.4610
1.4266
1.4183
1.4089
1.3916
0.9869
0.9697
0.9525
OH
CF3COO
N
OH
3d', CF3COOD, 25 °C, 1H
1.0205
1.0308
1.0337
1.0320
1.0000
2.0378
2.0566
2.0735
2.0829
4.1288
3.0417
[ppm]
[rel]

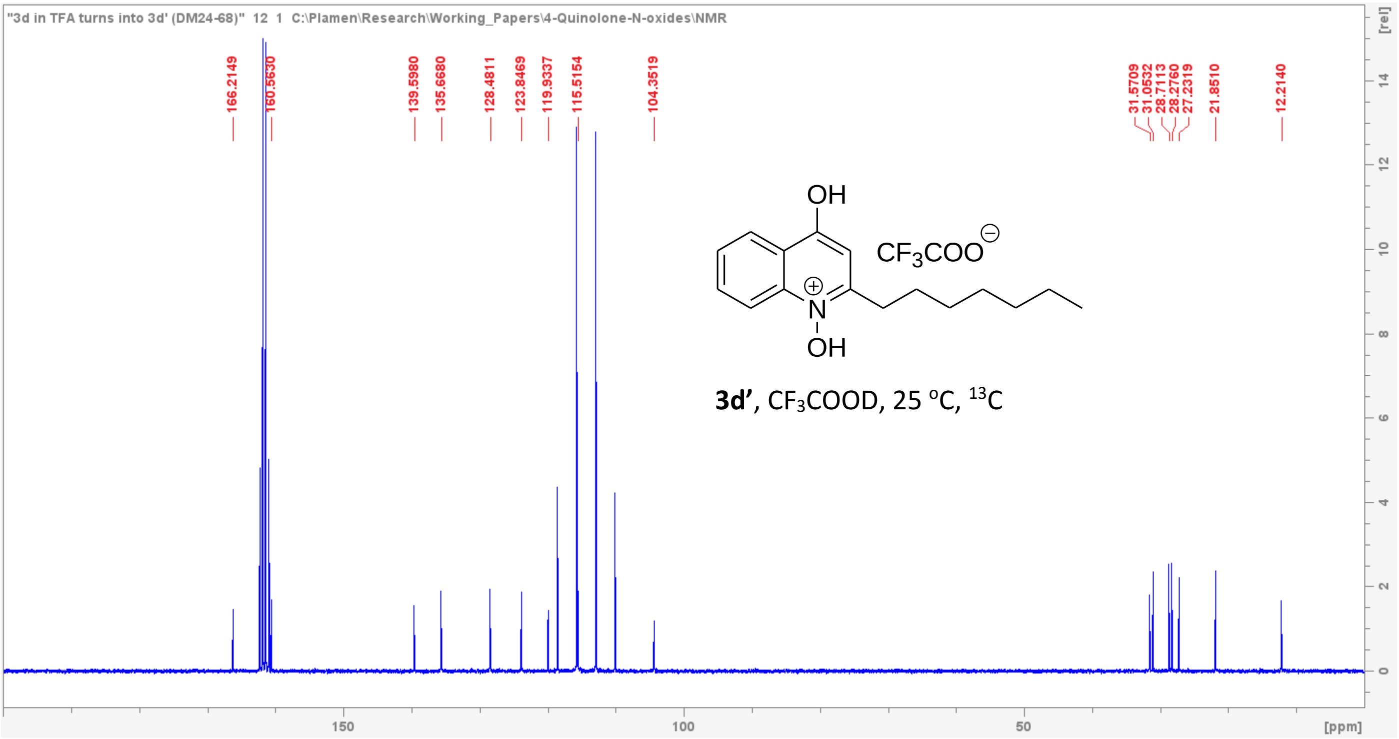
"3d in TFA turns into 3d' (DM24-68)" 12 1 C:\Plamen\Research\Working_Papers\4-Quinolone-N-oxides\NMR
166.2149
160.5630
139.5980
135.6680
128.4811
123.8469
119.9337
115.5154
104.3519
31.5709
31.0532
28.7113
28.2760
27.2319
21.8510
12.2140
OH
CF3COO
N
OH
3d', CF3COOD, 25 °C, 13C
[rel]
[ppm]

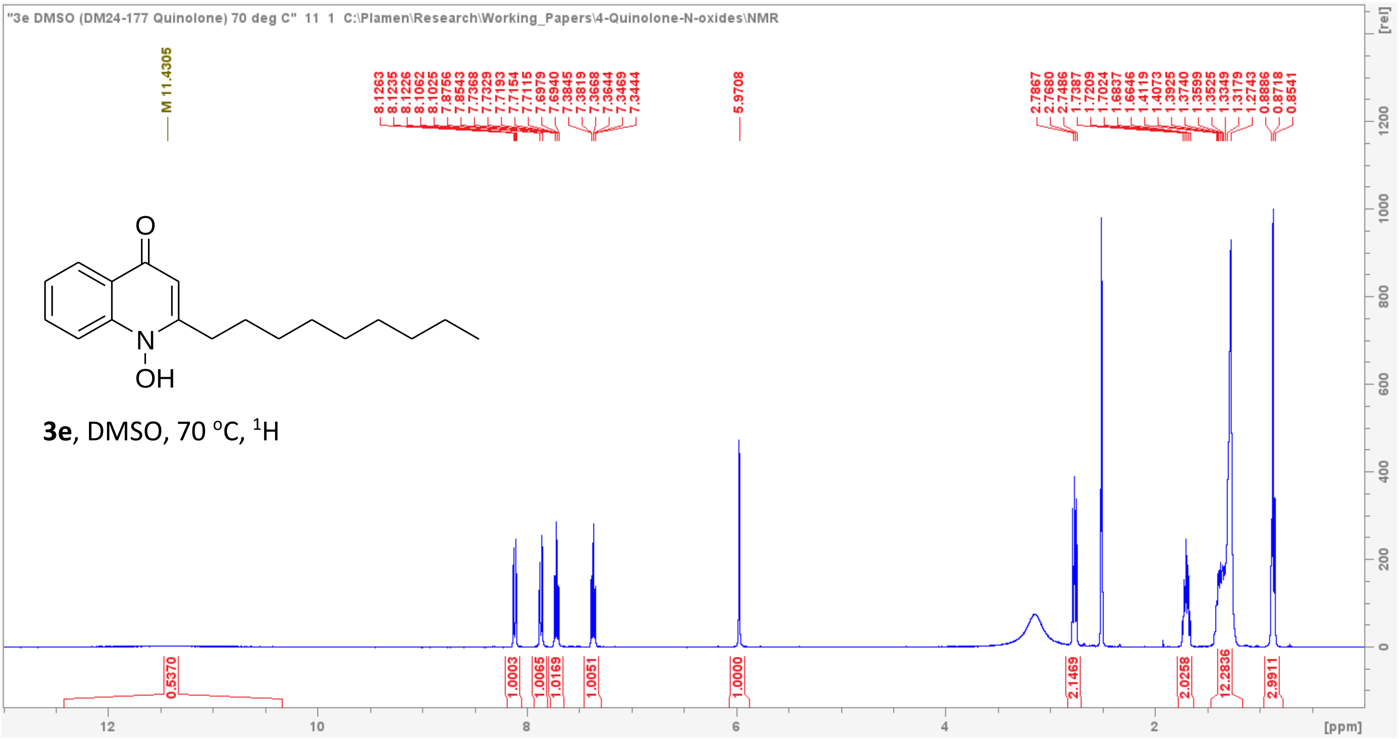
"3e DMSO (DM24-177 Quinolone) 70 deg C" 11 1 C:\Plamen\Research\Working_Papers\4-Quinolone-N-oxides\NMR
M 11.4305
5.9708
O
N
OH
3e, DMSO, 70 °C, 1H
0.5370
1.0003
1.0065
1.0169
1.0051
1.0000
2.1469
2.0258
12.2836
2.9911
[rel]
[ppm]

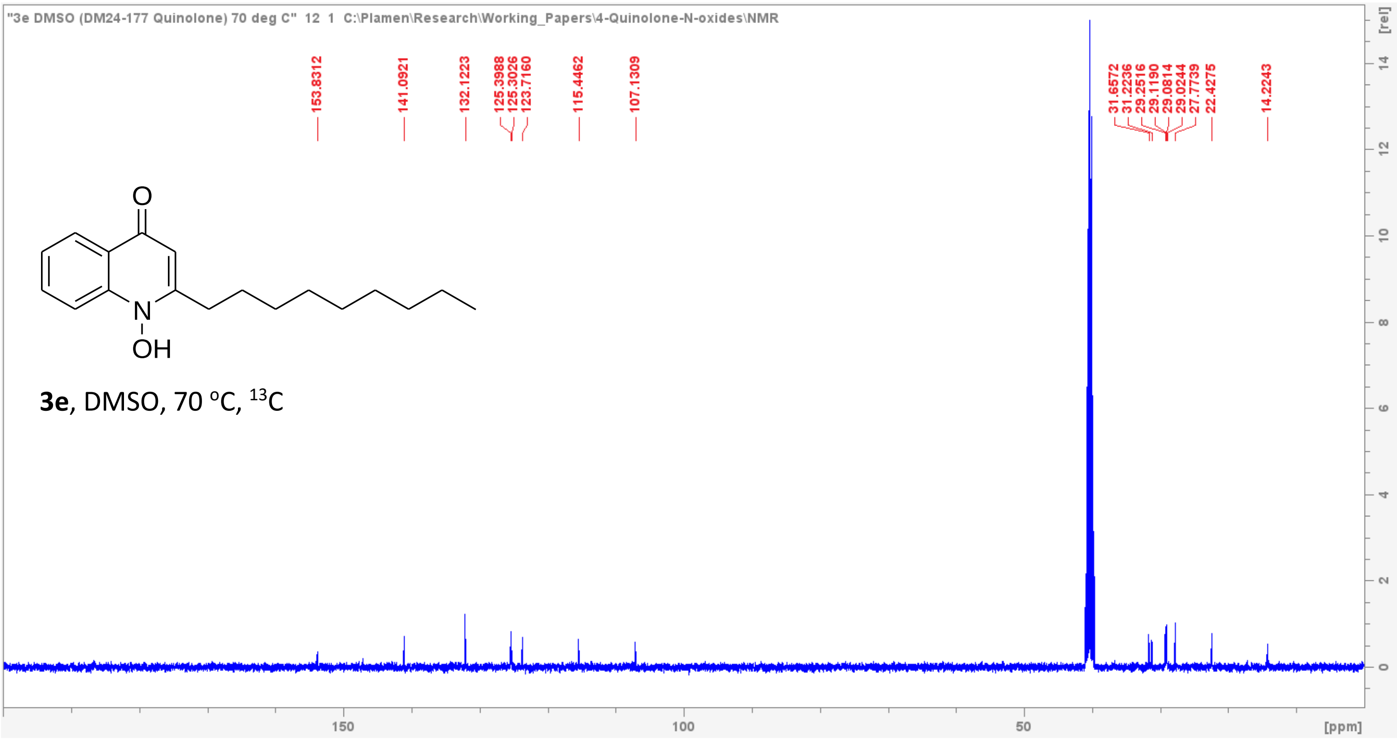

"3e DMSO (DM24-177 Quinolone) 70 deg C" 12 1 C:\Plamen\Research\Working_Papers\4-Quinolone-N-oxides\NMR
153.8312
141.0921
132.1223
125.3988
125.3026
123.7160
115.4462
107.1309
31.6572
31.2236
29.2516
29.1190
29.0814
29.0244
27.7739
22.4275
14.2243
O
N
OH
3e, DMSO, 70 °C, 13C
[rel]
[ppm]

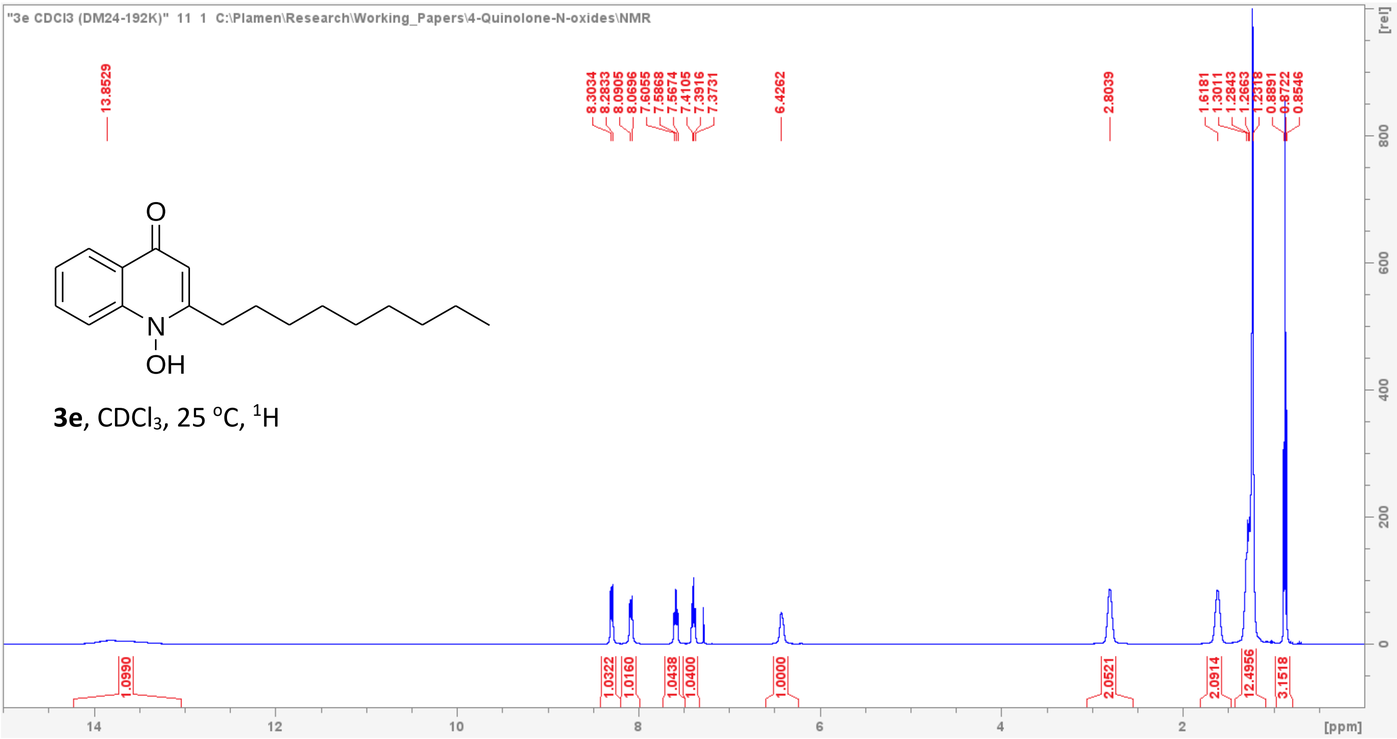

"3e CDCl3 (DM24-192K)" 11 1 C:\Plamen\Research\Working_Papers\4-Quinolone-N-oxides\NMR
13.8529
8.3034
8.2833
8.0905
8.0696
7.6055
7.5868
7.5674
7.4105
7.3916
7.3731
6.4262
2.8039
1.6181
1.3011
1.2843
1.2663
1.2318
0.8891
0.8722
0.8546
O
N
OH
3e, CDCl3, 25 °C, 1H
1.0990
1.0322
1.0160
1.0438
1.0400
1.0000
2.0521
2.0914
12.4956
3.1518
[rel]
[ppm]

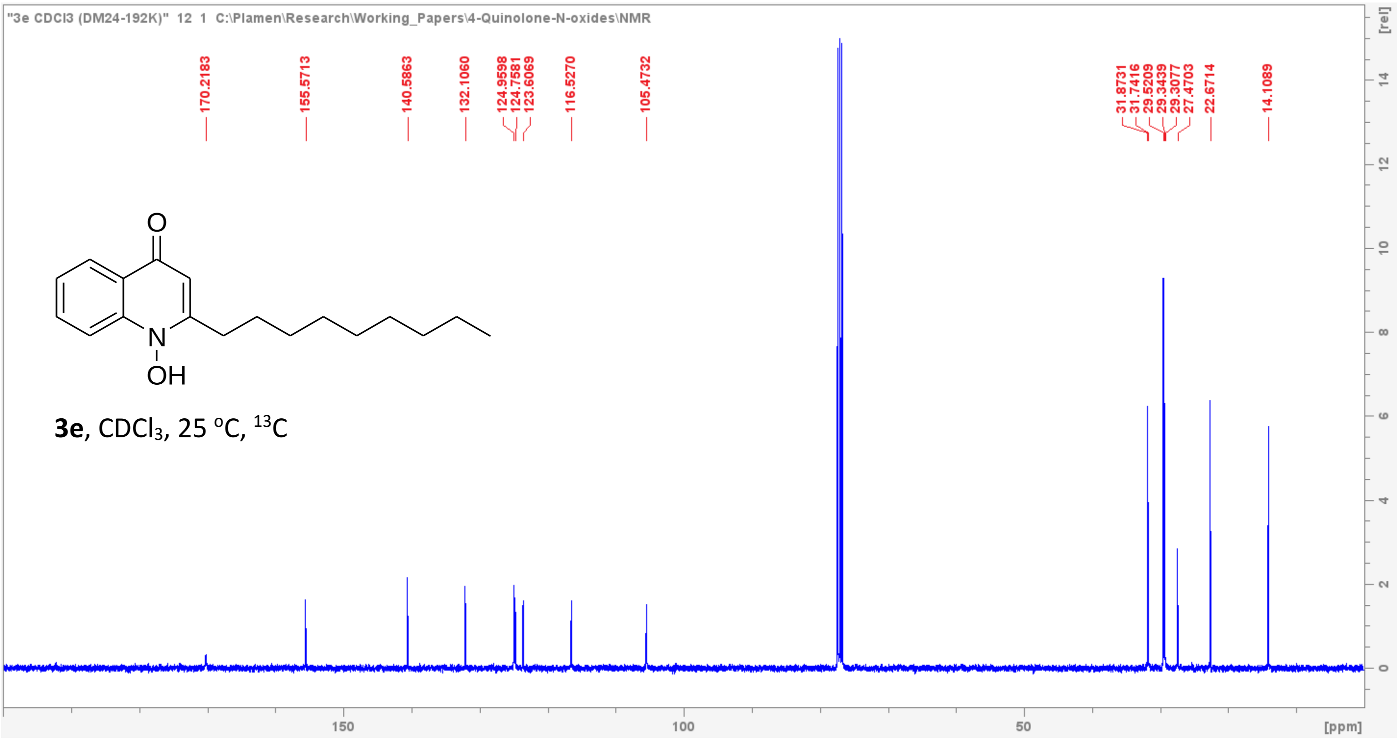
"3e CDCl3 (DM24-192K)" 12 1 C:\Plamen\Research\Working_Papers\4-Quinolone-N-oxides\NMR
170.2183
155.5713
140.5863
132.1060
124.9598
124.7581
123.6069
116.5270
105.4732
31.8731
31.7416
29.5209
29.3439
29.3077
27.4703
22.6714
14.1089
O
N
OH
3e, CDCl3, 25 °C, 13C
[rel]
[ppm]

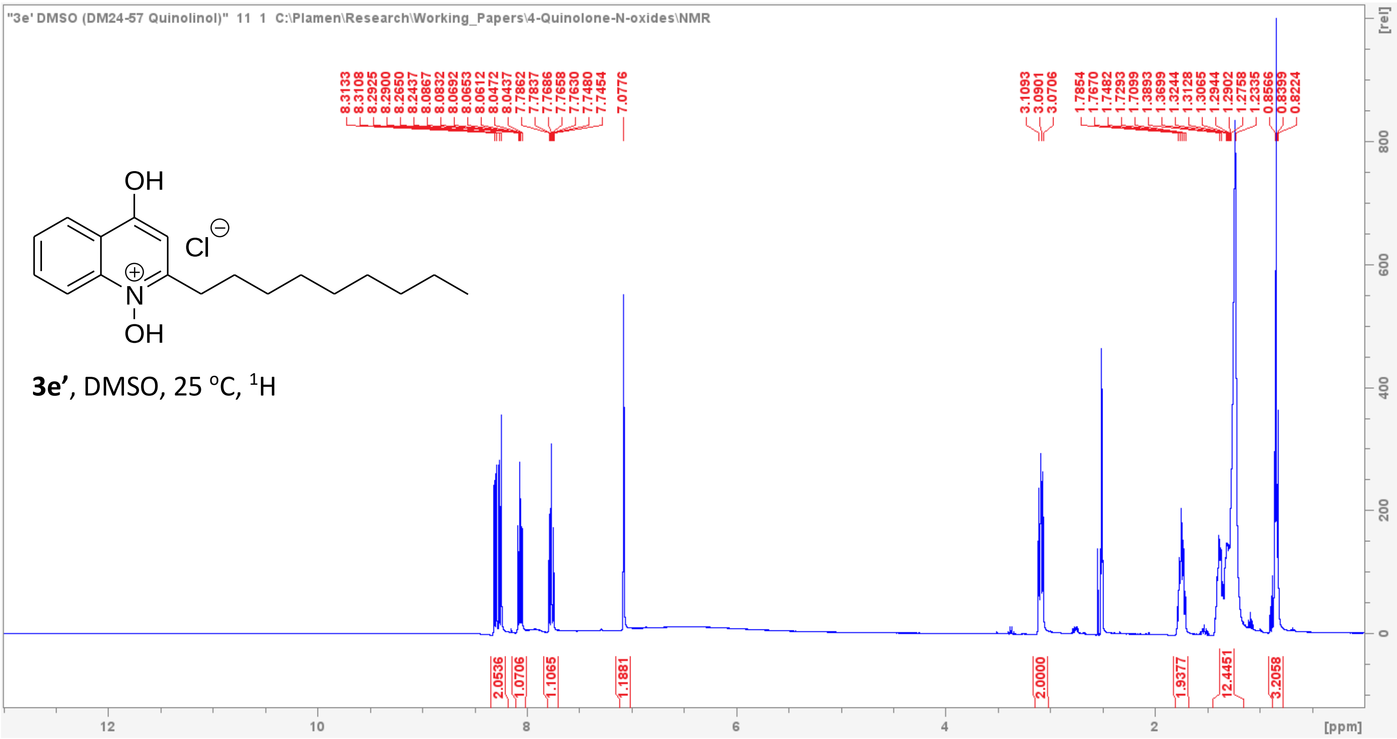
"3e' DMSO (DM24-57 Quinolinol)" 11 1 C:\Plamen\Research\Working_Papers\4-Quinolone-N-oxides\NMR
OH
Cl
N
OH
3e', DMSO, 25 °C, 1H
2.0536
1.0706
1.1065
1.1881
2.0000
1.9377
12.4451
3.2058
[rel]
[ppm]

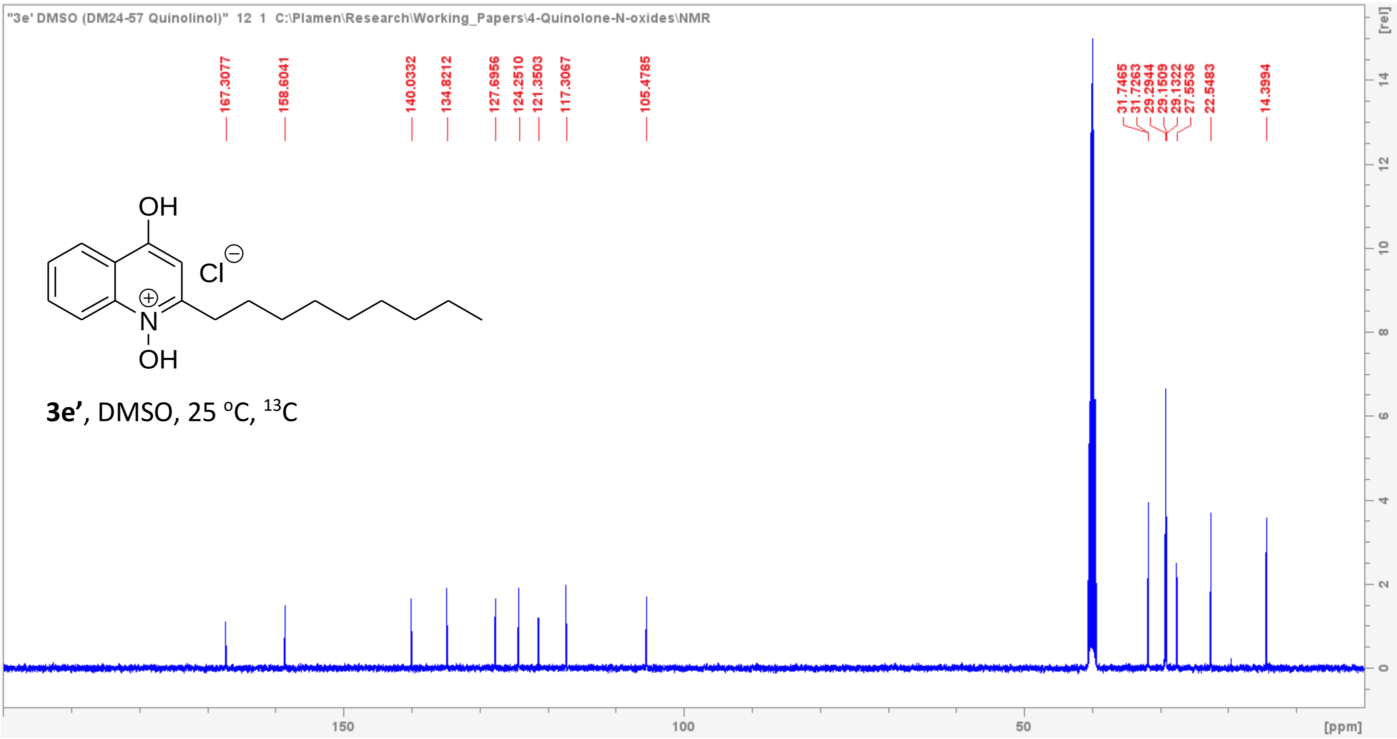
"3e' DMSO (DM24-57 Quinolinol)" 12 1 C:\Plamen\Research\Working_Papers\4-Quinolone-N-oxides\NMR
167.3077
158.6041
140.0332
134.8212
127.6956
124.2510
121.3503
117.3067
105.4785
31.7465
31.7263
29.2944
29.1509
29.1322
27.5536
22.5483
14.3994
OH
Cl
N
OH
3e', DMSO, 25 °C, 13C
[ppm]
[rel]

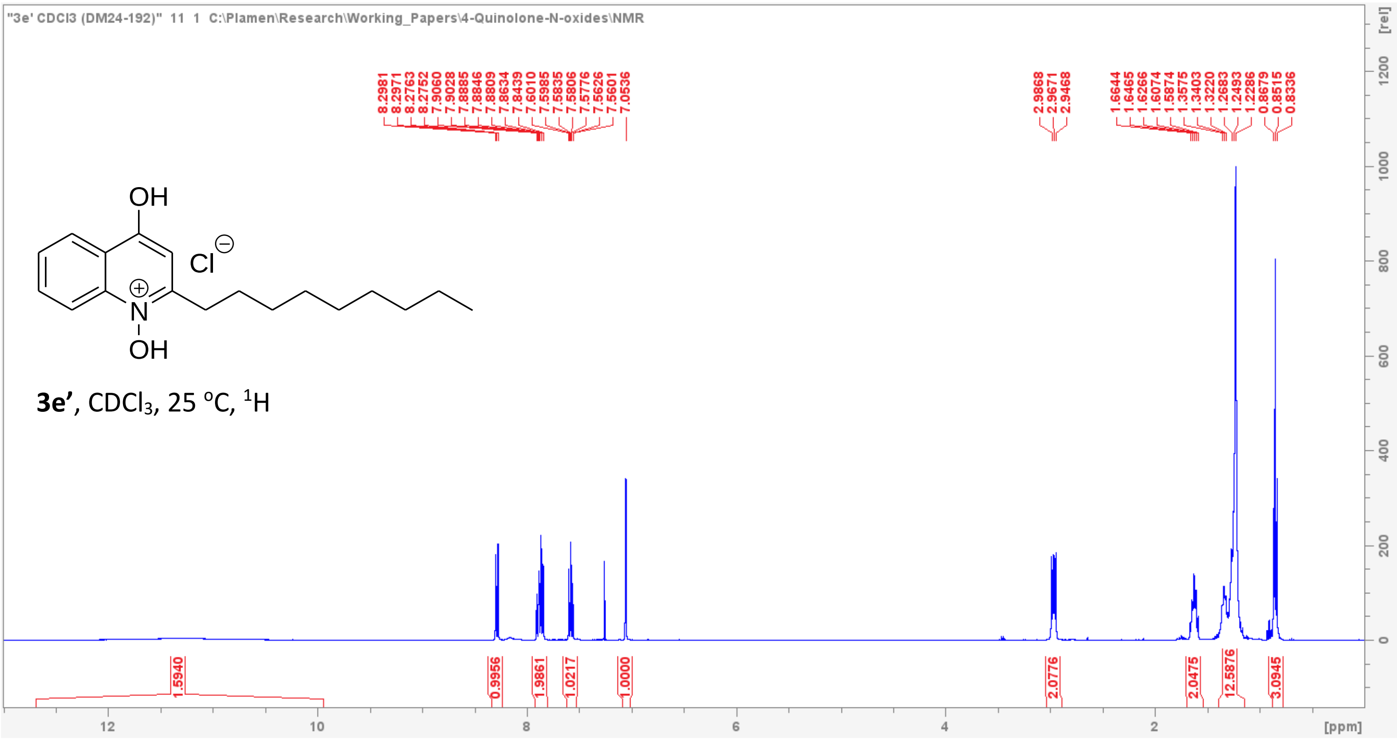
"3e' CDCl3 (DM24-192)" 11 1 C:\Plamen\Research\Working_Papers\4-Quinolone-N-oxides\NMR
8.2981
8.2971
8.2763
8.2752
7.9060
7.9028
7.8885
7.8846
7.8809
7.8634
7.8439
7.6010
7.5985
7.5835
7.5806
7.5776
7.5626
7.5601
7.0536
2.9868
2.9671
2.9468
1.6644
1.6465
1.6266
1.6074
1.5874
1.3575
1.3403
1.3220
1.2683
1.2493
1.2286
0.8679
0.8515
0.8336
OH
Cl
N
OH
3e', CDCl3, 25 °C, 1H
1.5940
0.9956
1.9861
1.0217
1.0000
2.0776
2.0475
12.5876
3.0945
[ppm]
[rel]

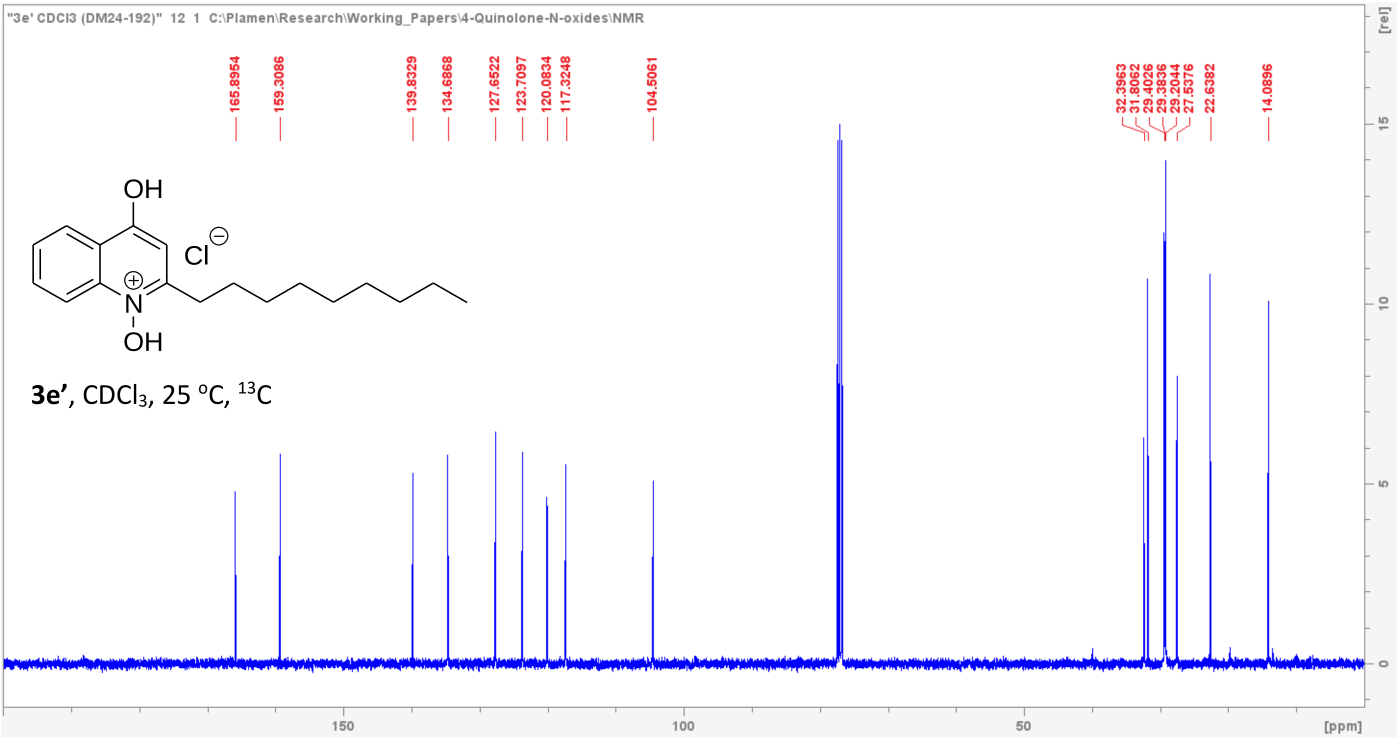
"3e' CDCl3 (DM24-192)" 12 1 C:\Plamen\Research\Working_Papers\4-Quinolone-N-oxides\NMR
165.8954
159.3086
139.8329
134.6868
127.6522
123.7097
120.0834
117.3248
104.5061
32.3963
31.8062
29.4026
29.3836
29.2044
27.5376
22.6382
14.0896
[rel]
[ppm]
3e', CDCl3, 25 °C, 13C

NMR spectra of 3/3'' in CD_3OD (80/20 MHz Magritek Spinsolve 80)

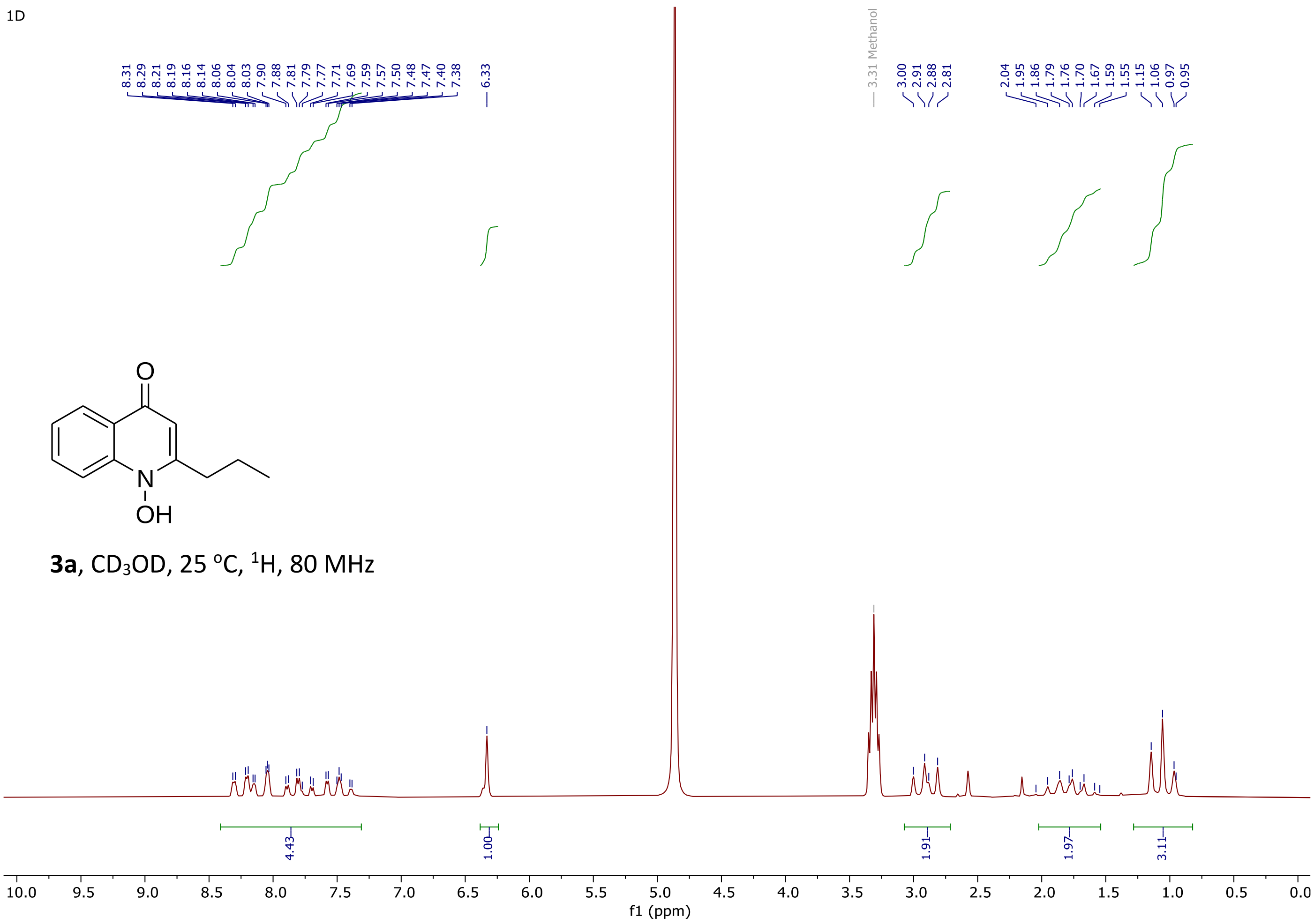

3a, CD_3OD, 25 °C, ^1H, 80 MHz

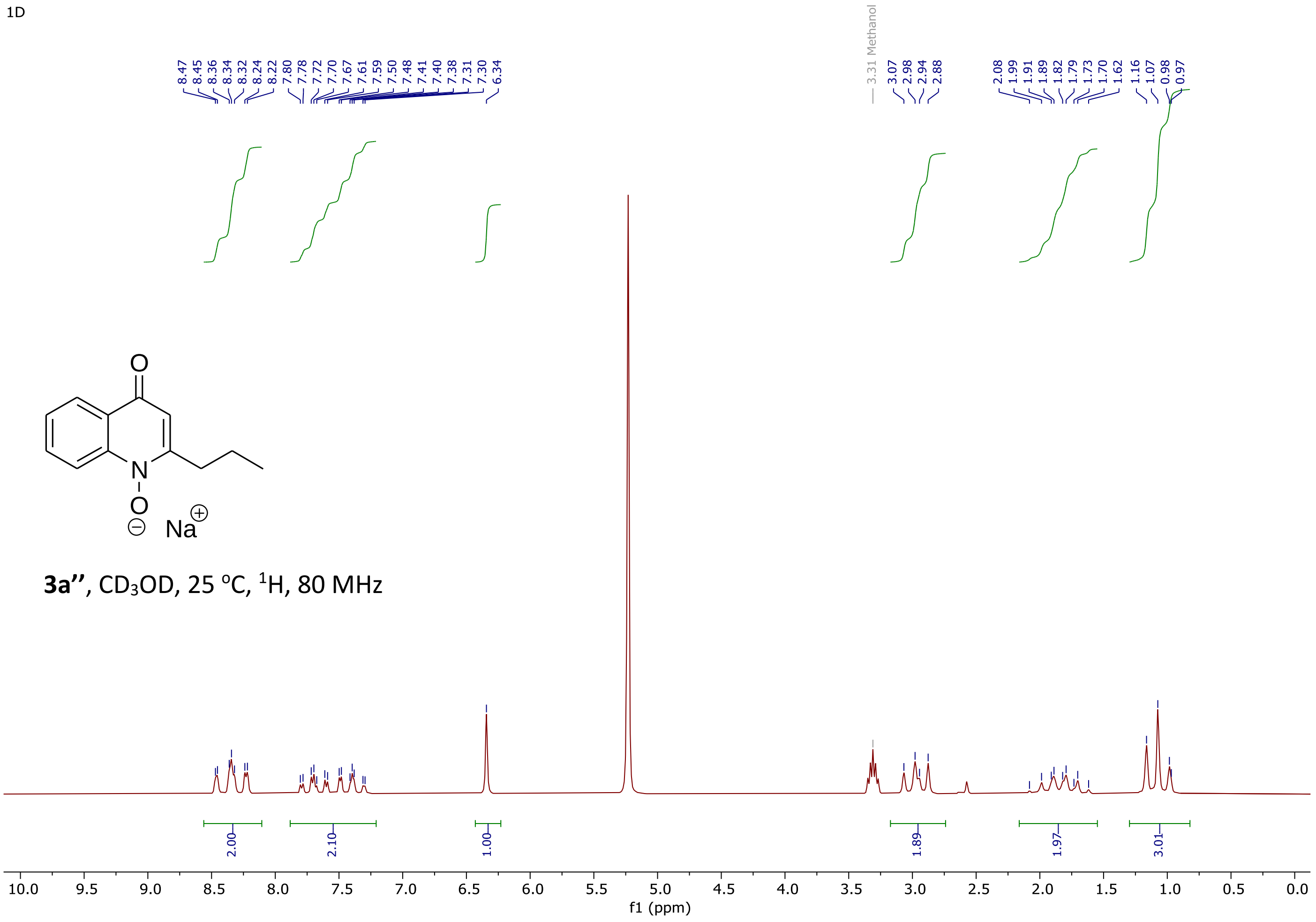

3a'', CD_3OD, 25 °C, ^1H, 80 MHz

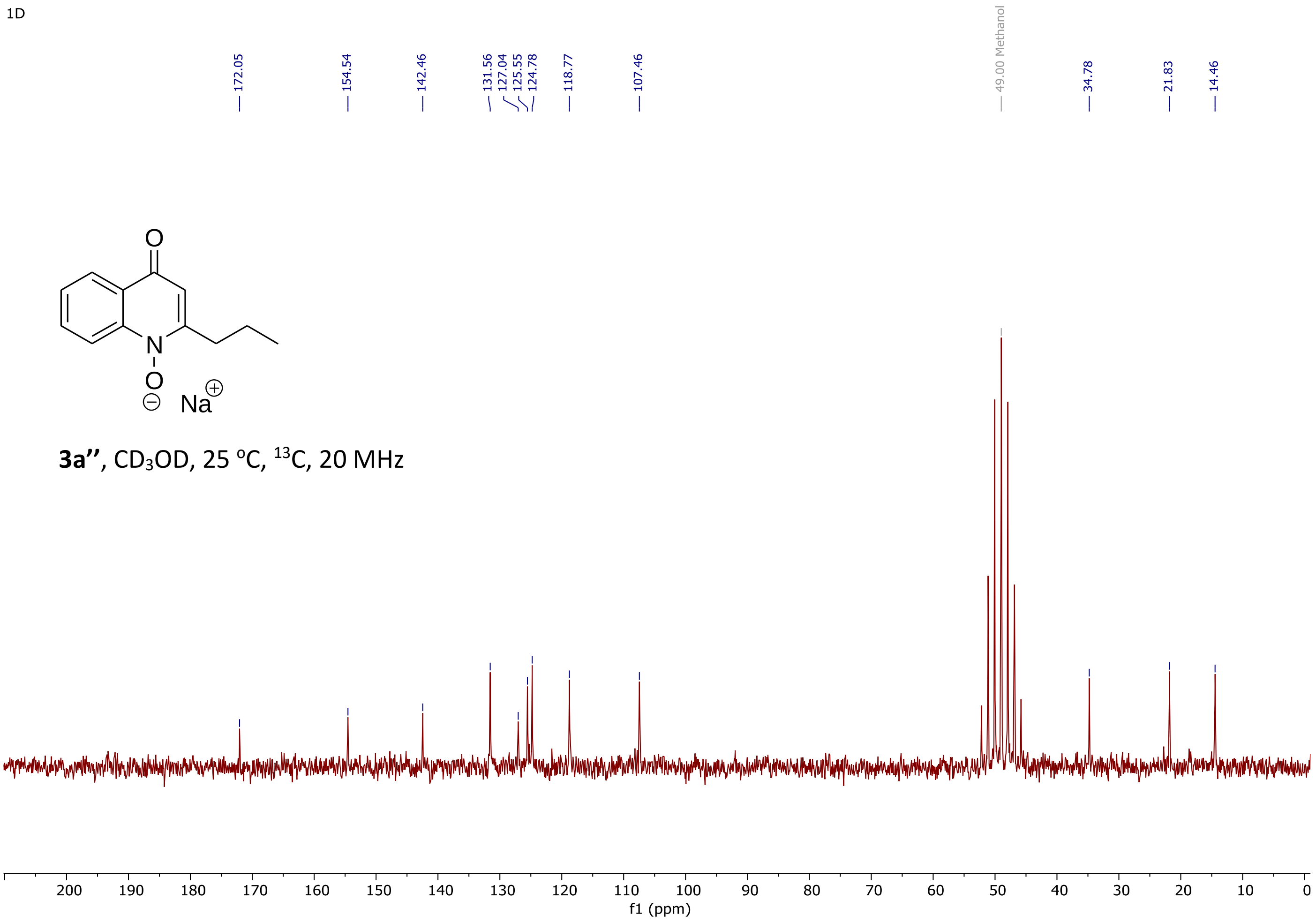

3a'', CD_3OD, 25 °C, ^{13}C, 20 MHz

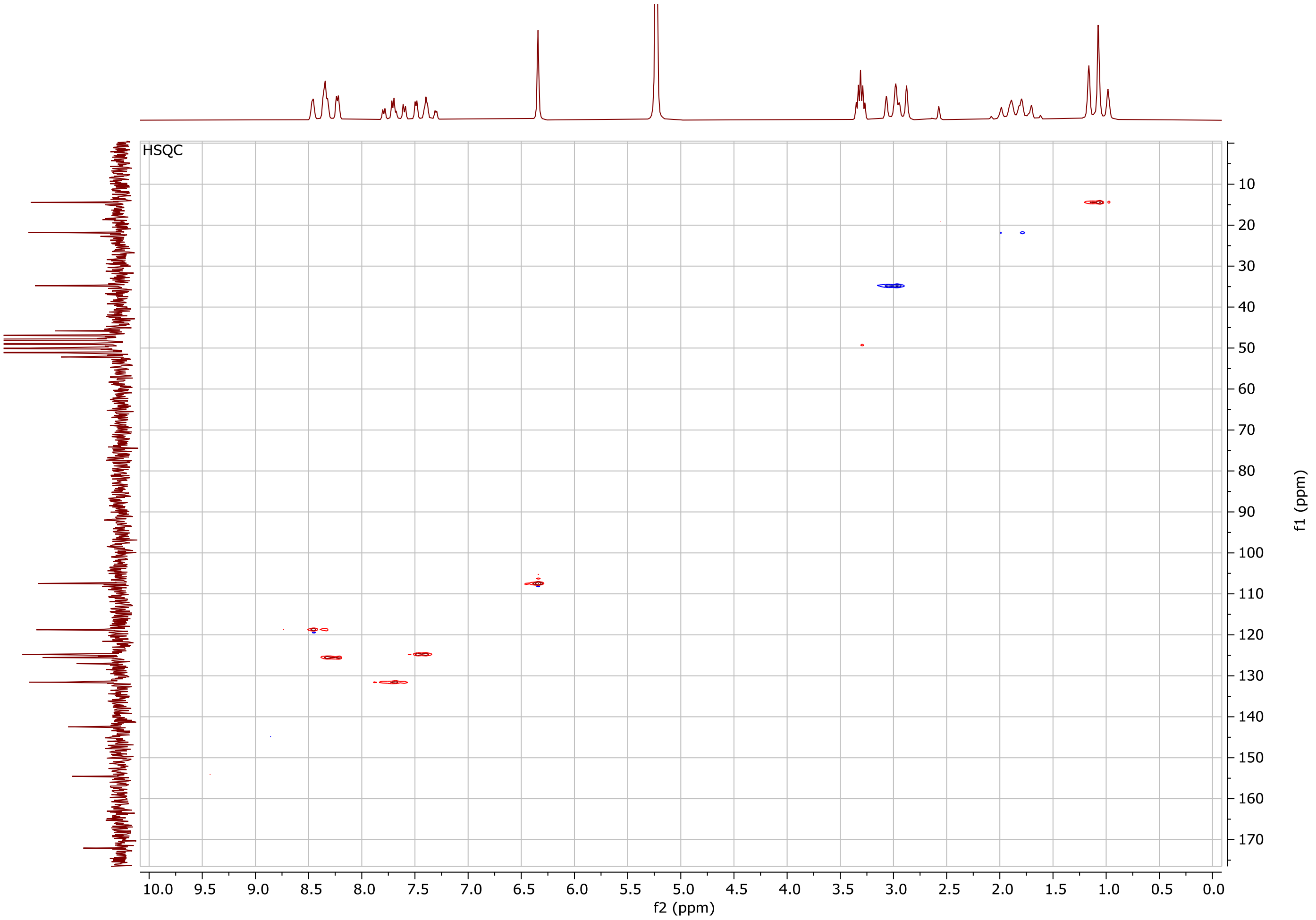
HSQC
f1 (ppm)
f2 (ppm)

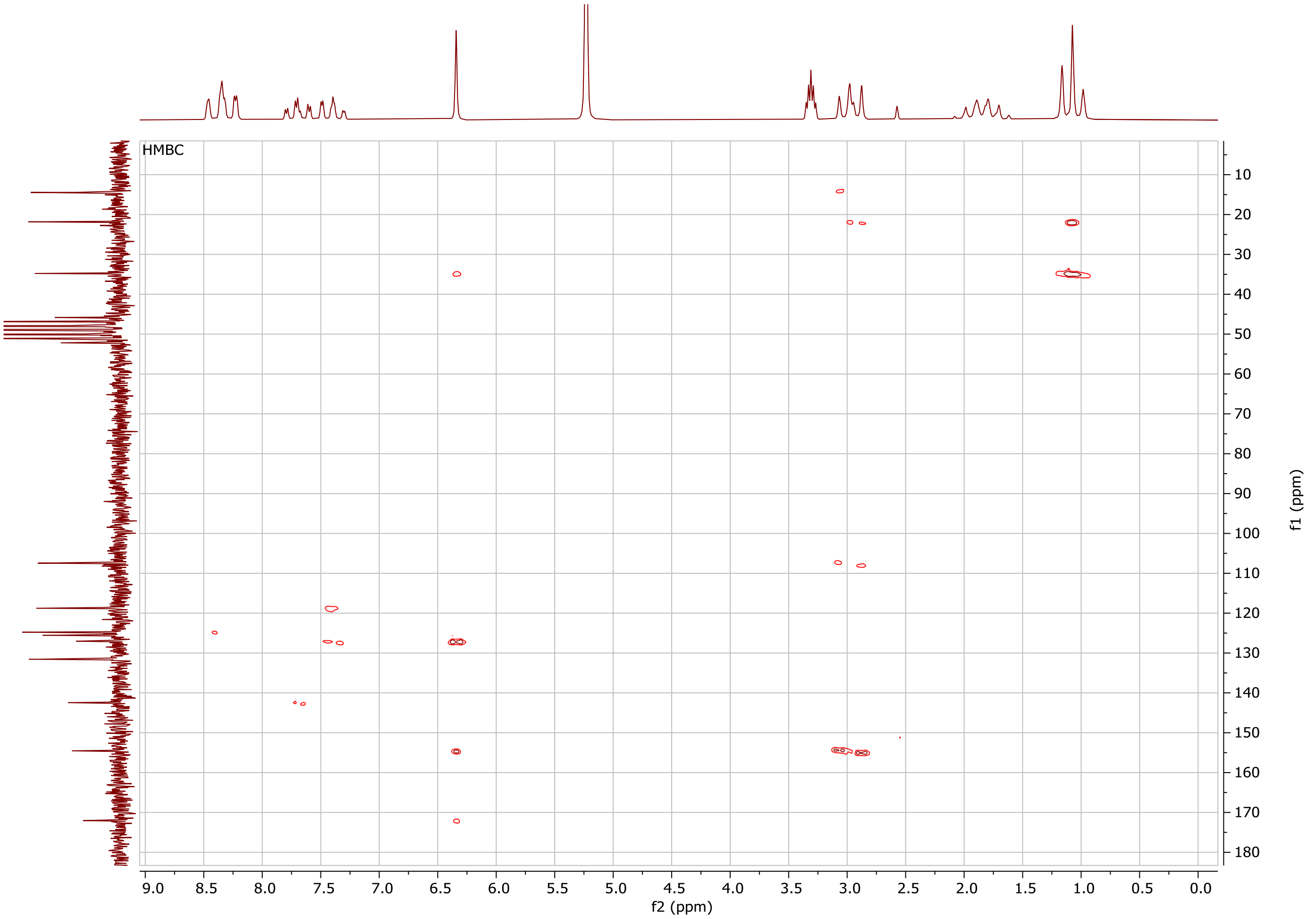
HMBC
f1 (ppm)
f2 (ppm)

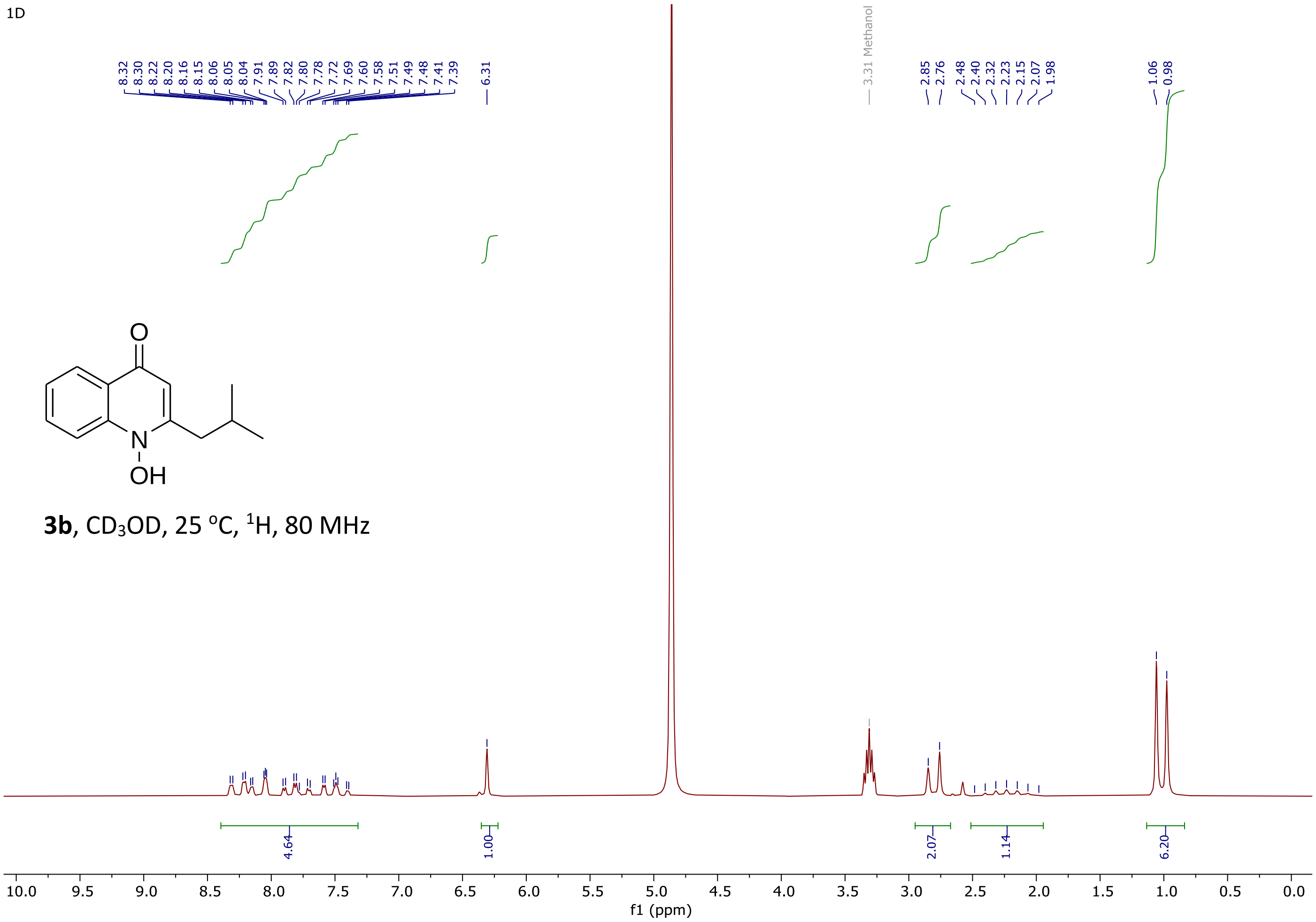

3b, CD_3OD, 25 °C, ^1H, 80 MHz

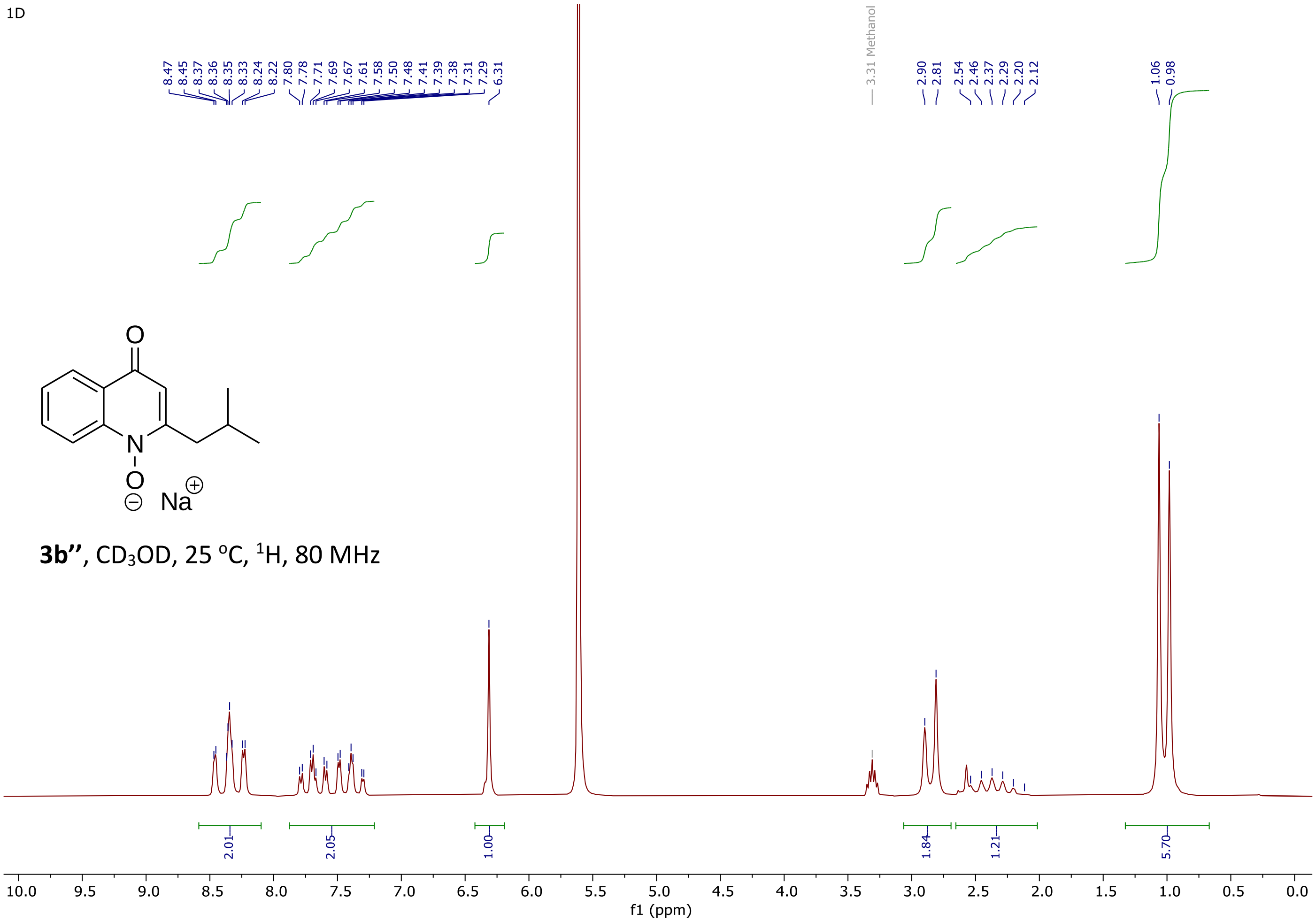

3b'', CD_3OD, 25 °C, ^1H, 80 MHz

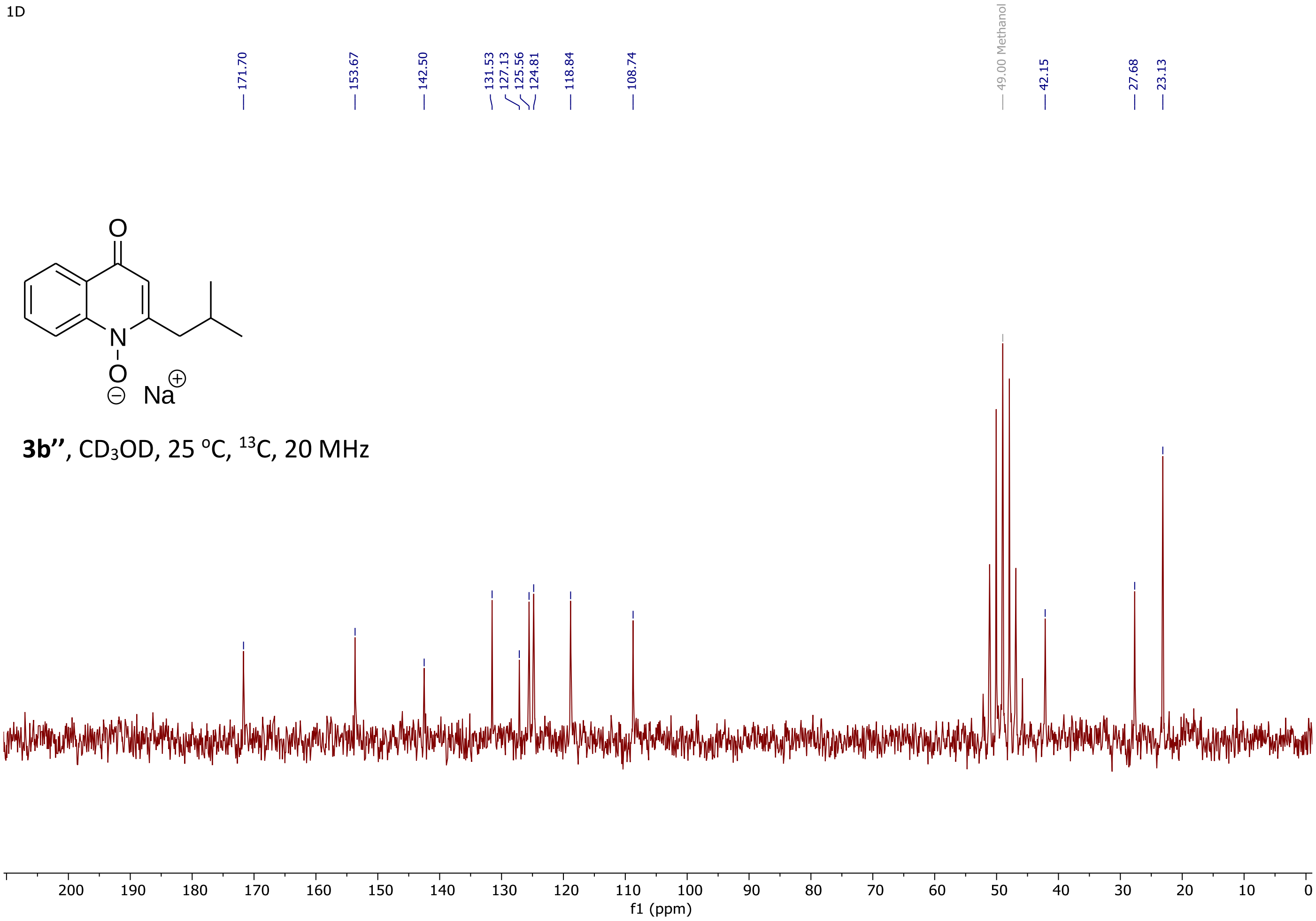

3b'', CD_3OD, 25 °C, ^{13}C, 20 MHz

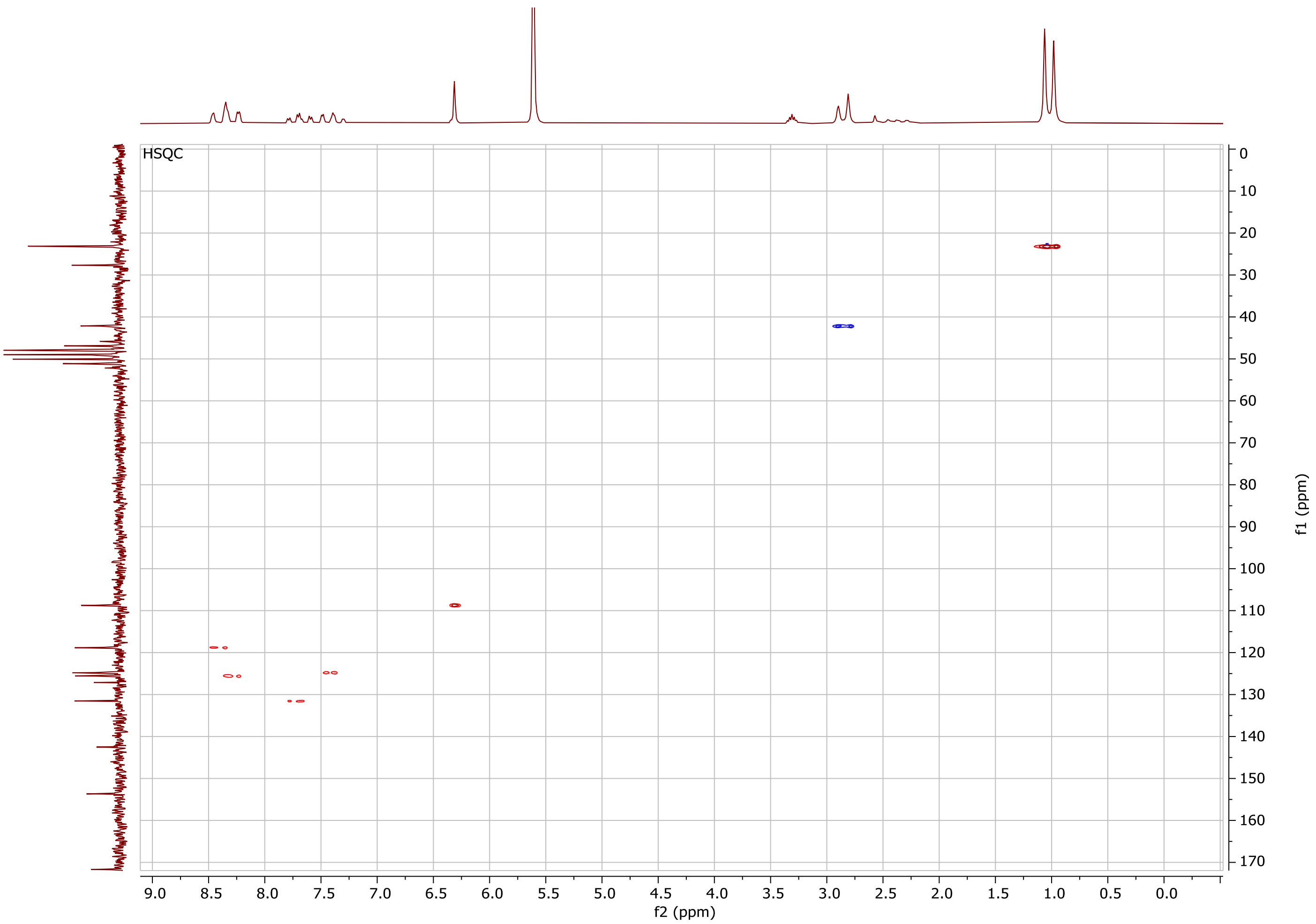
HSQC
f1 (ppm)
f2 (ppm)

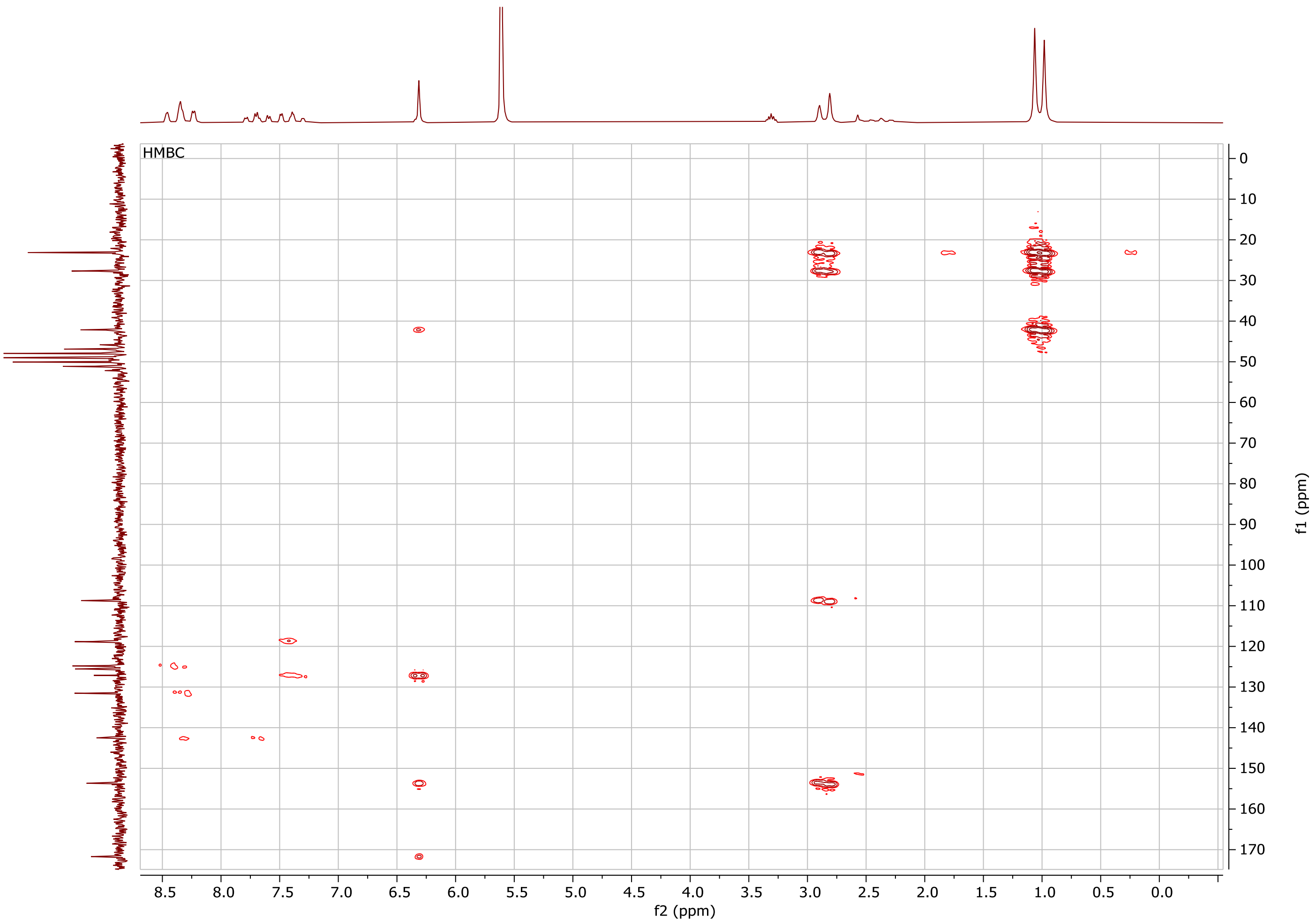
HMBC
f1 (ppm)
f2 (ppm)

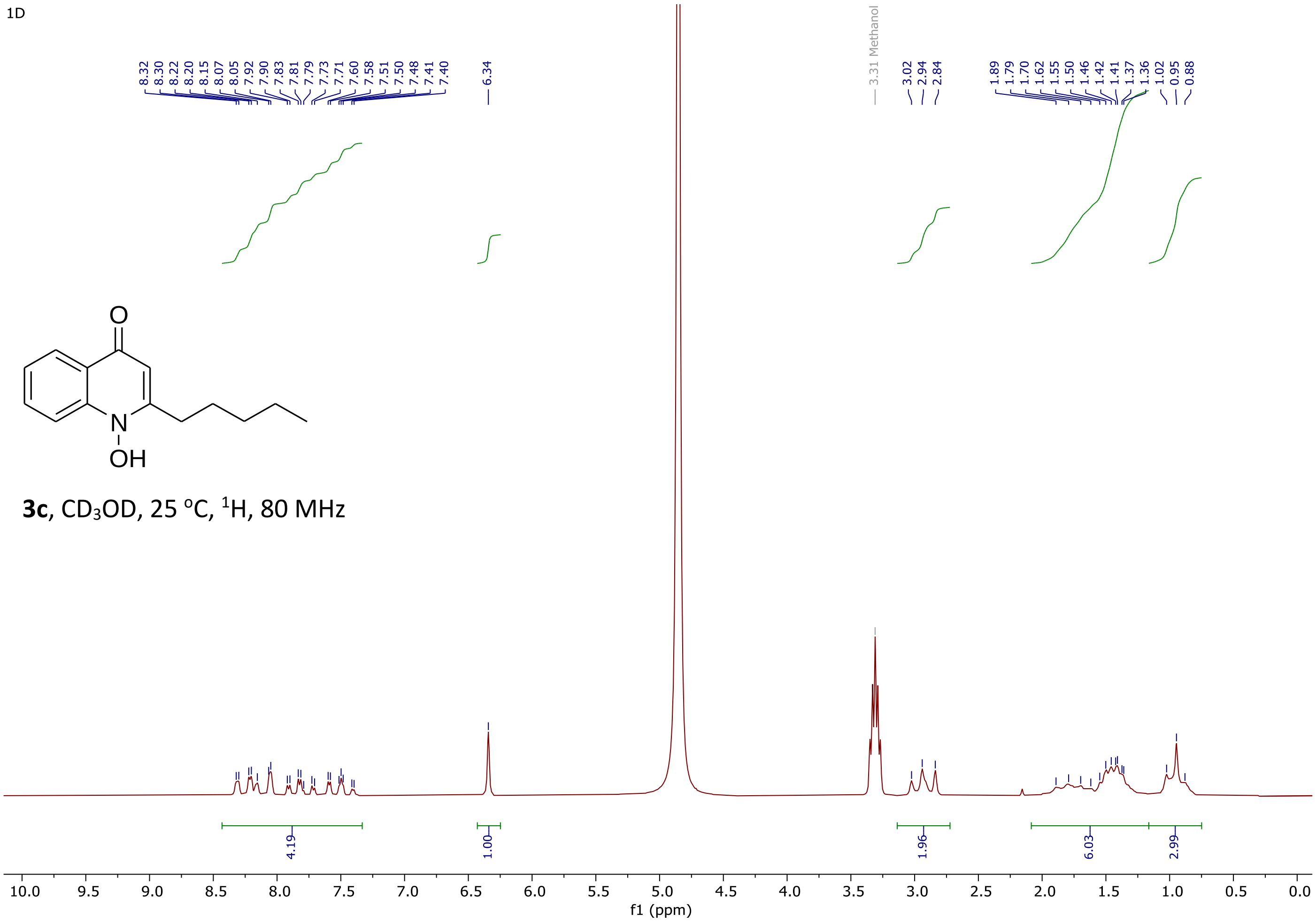

3c, CD_3OD, 25 °C, ^1H, 80 MHz

1D

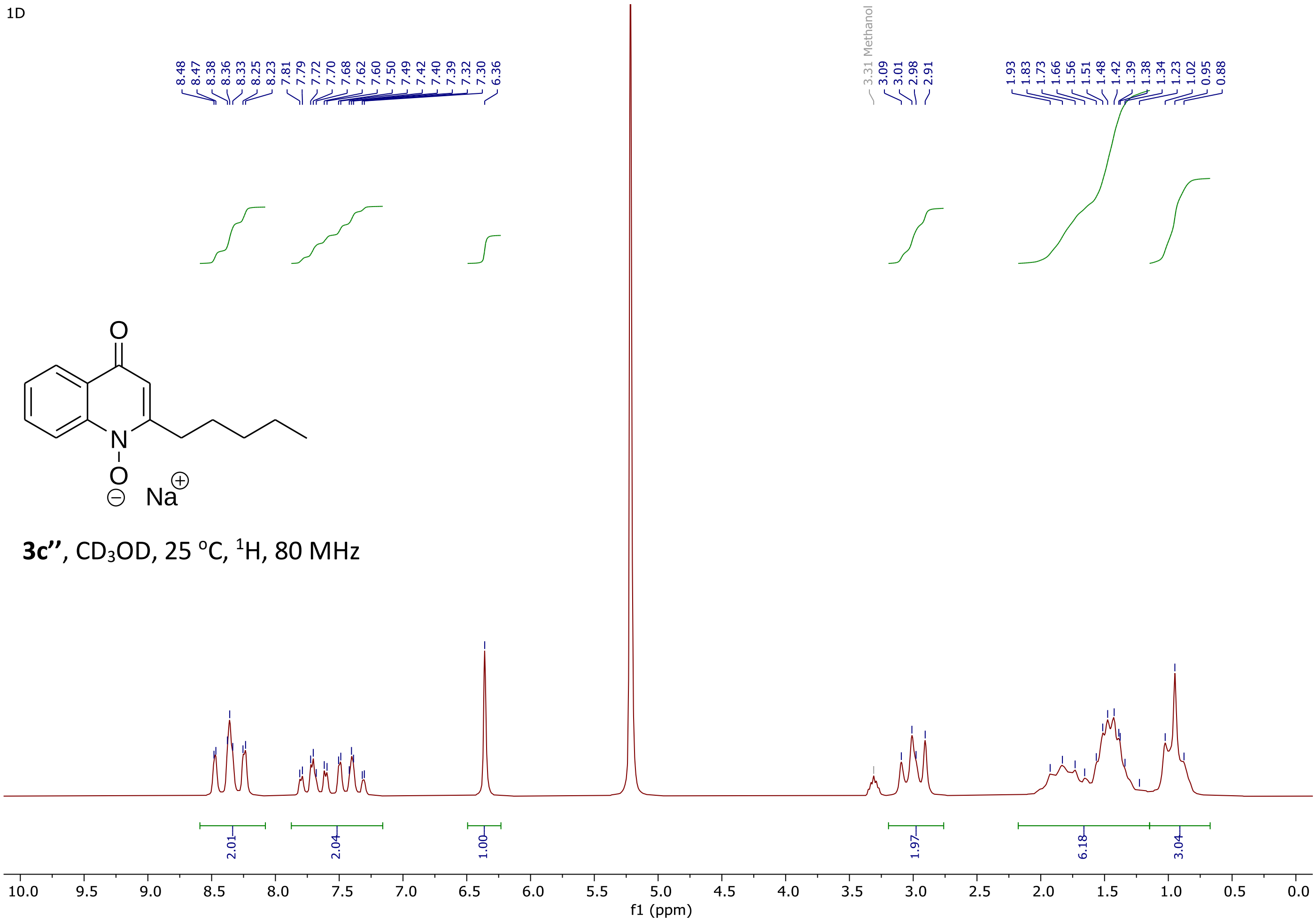

3c'', CD_3OD, 25 °C, ^{1}H, 80 MHz

1D

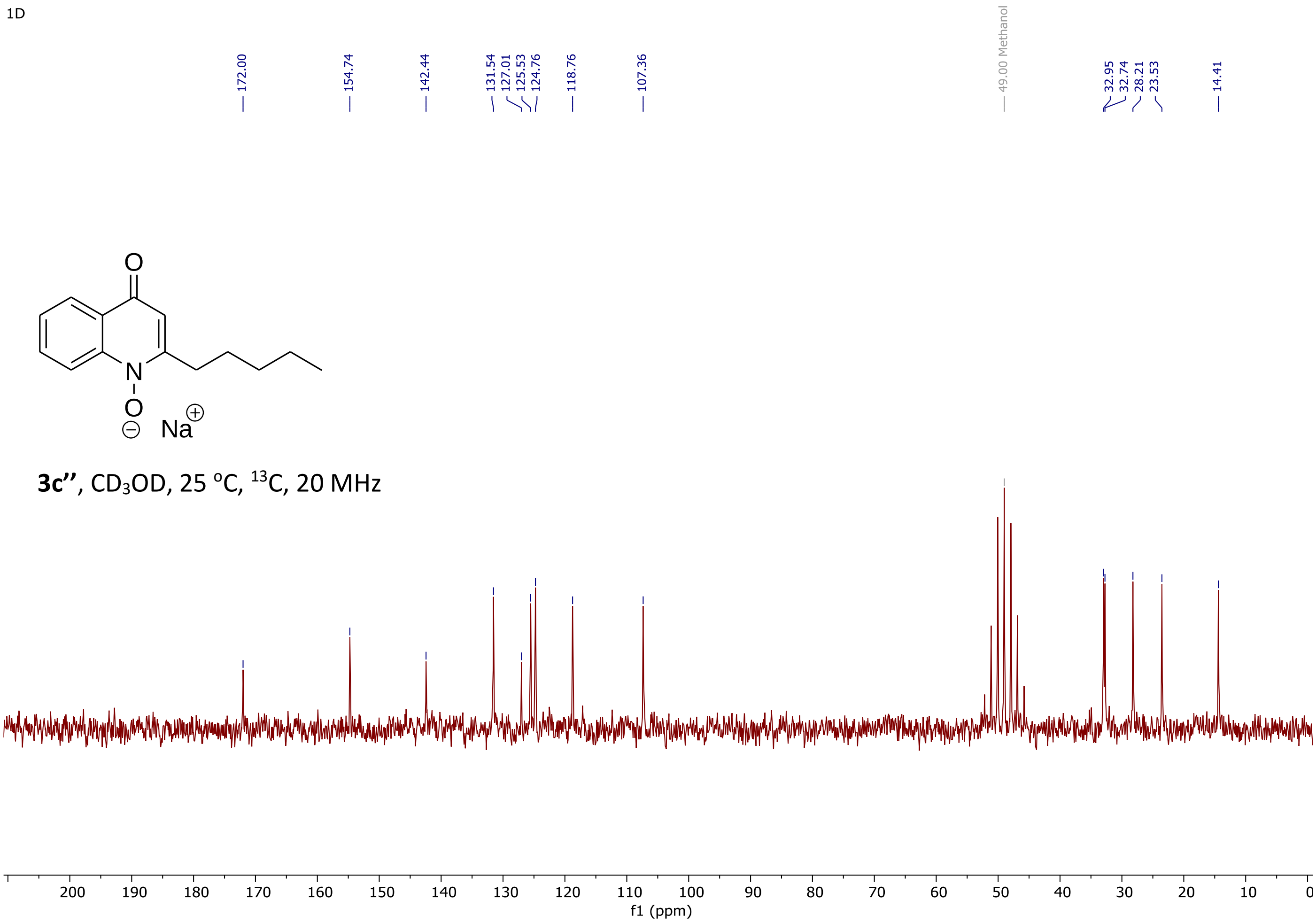

3c'', CD_3OD, 25 °C, ^{13}C, 20 MHz

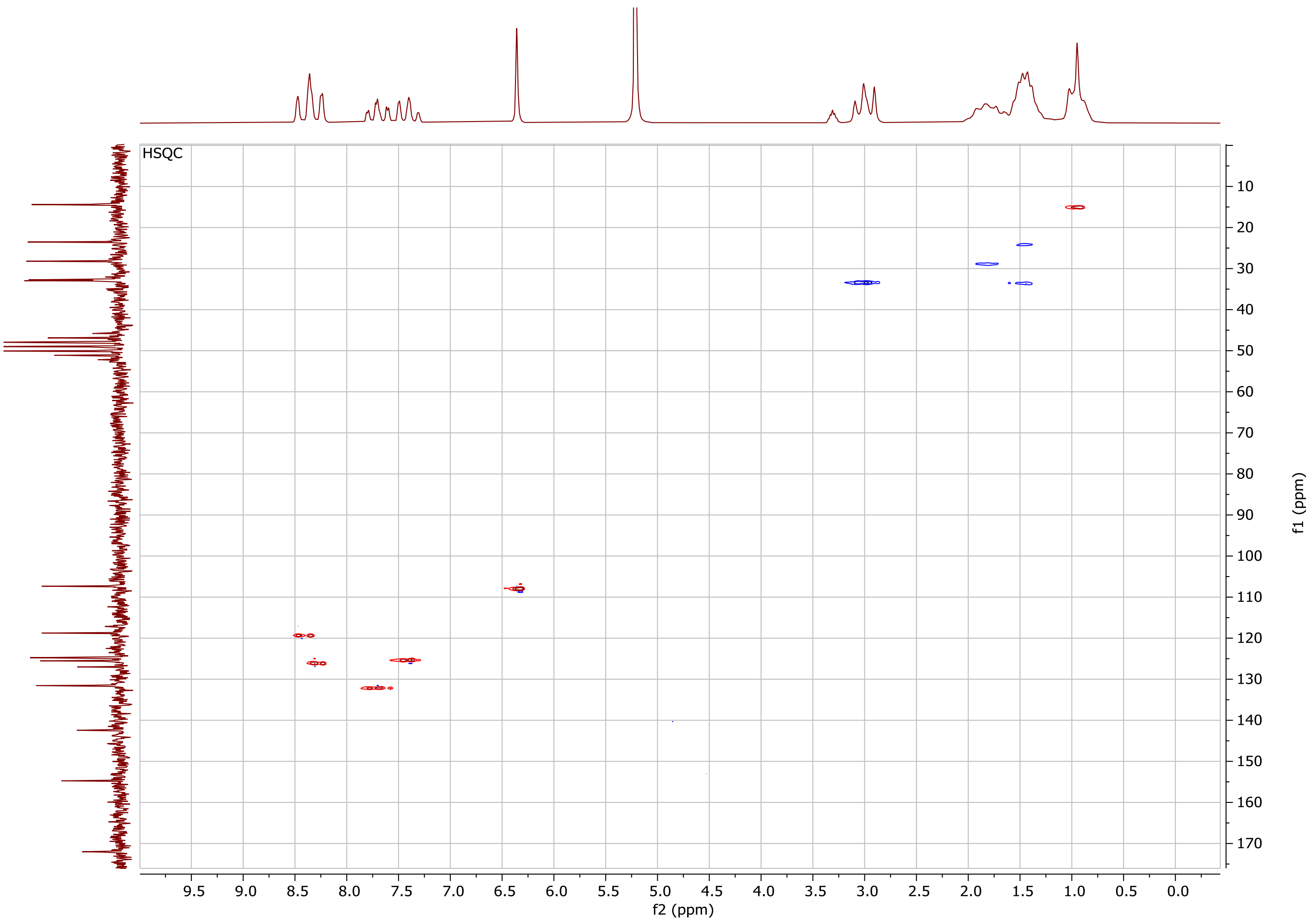
HSQC
f2 (ppm)
f1 (ppm)

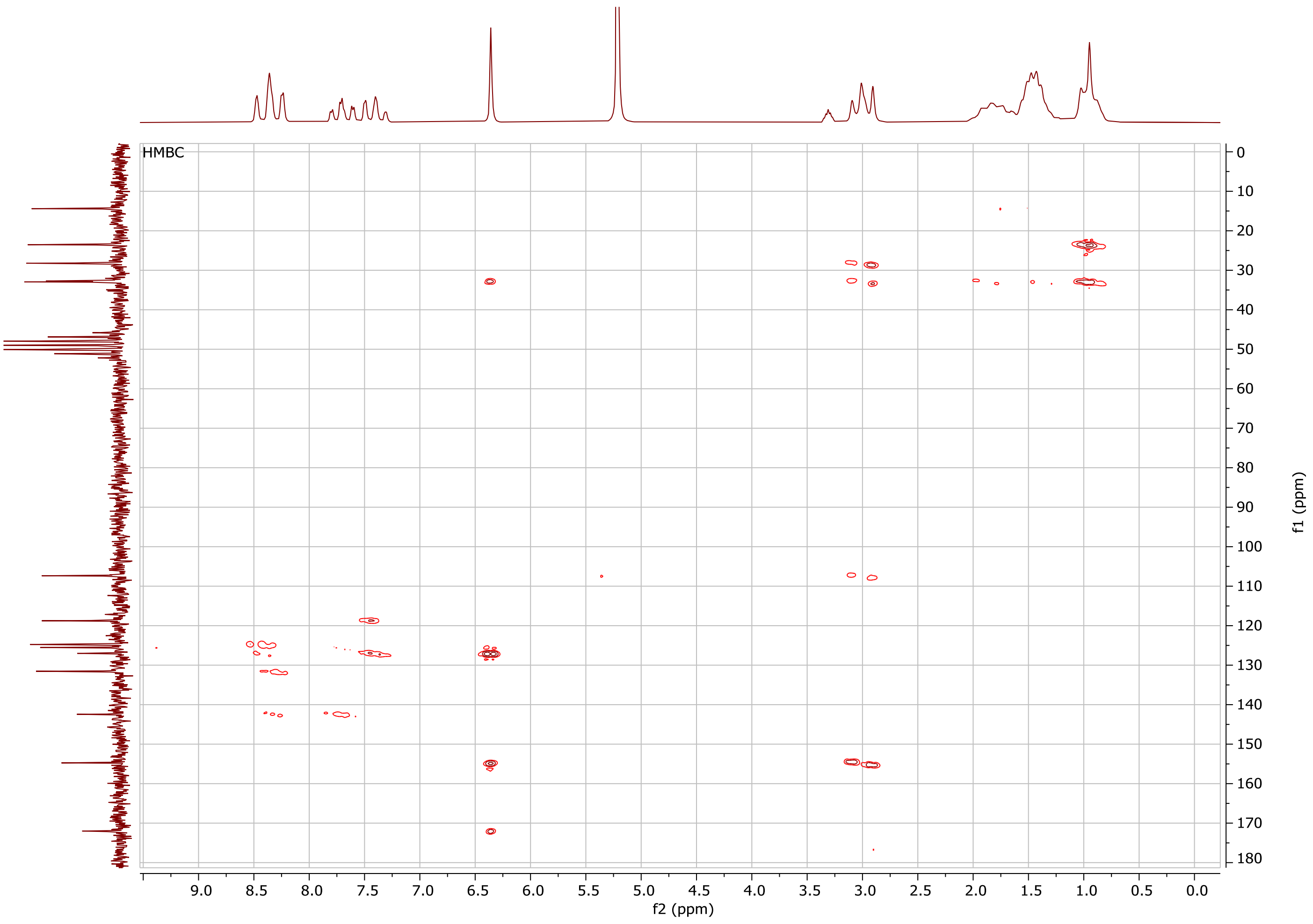
HMBC
f2 (ppm)
f1 (ppm)

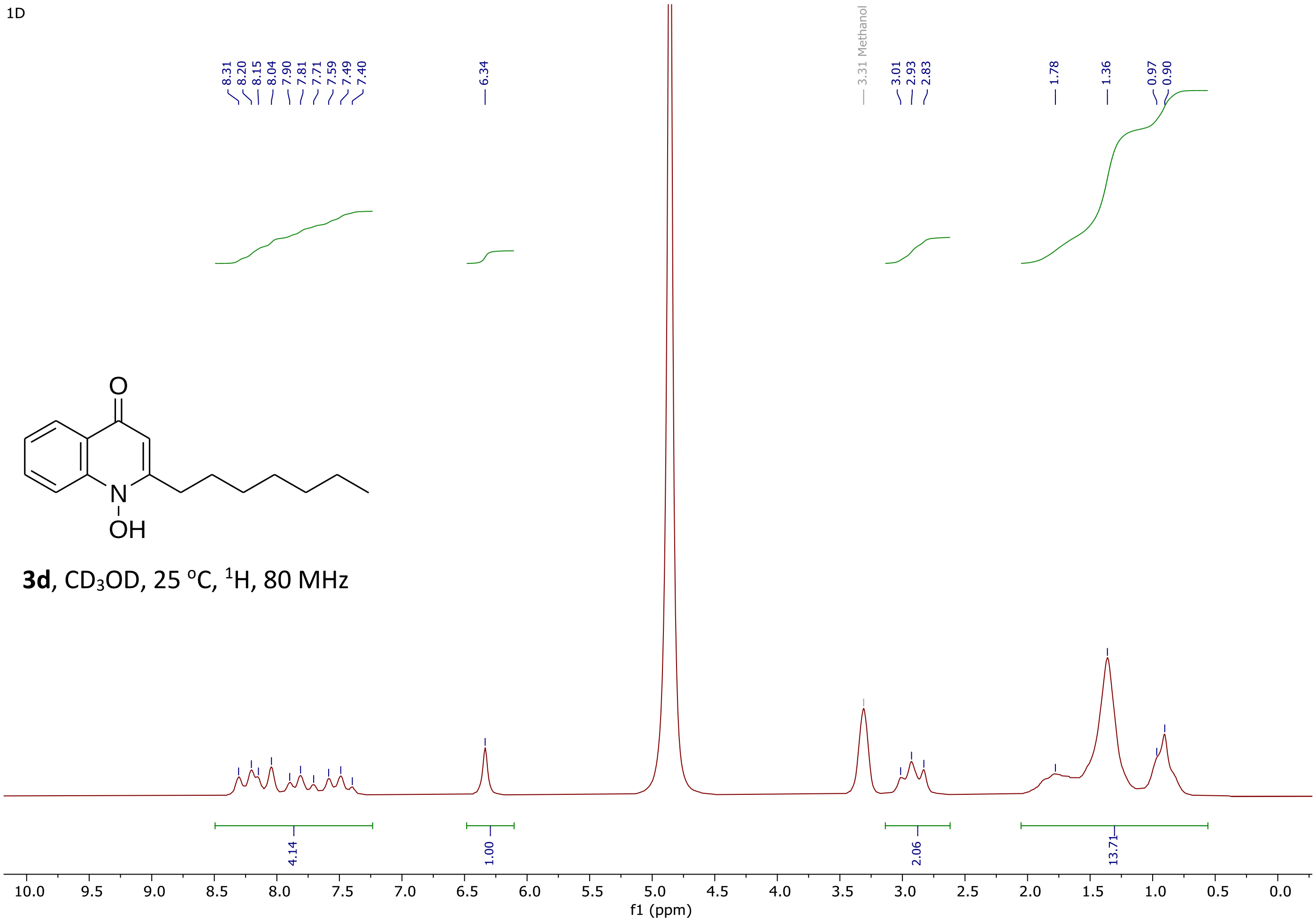

3d, CD_3OD, 25 °C, ^1H, 80 MHz

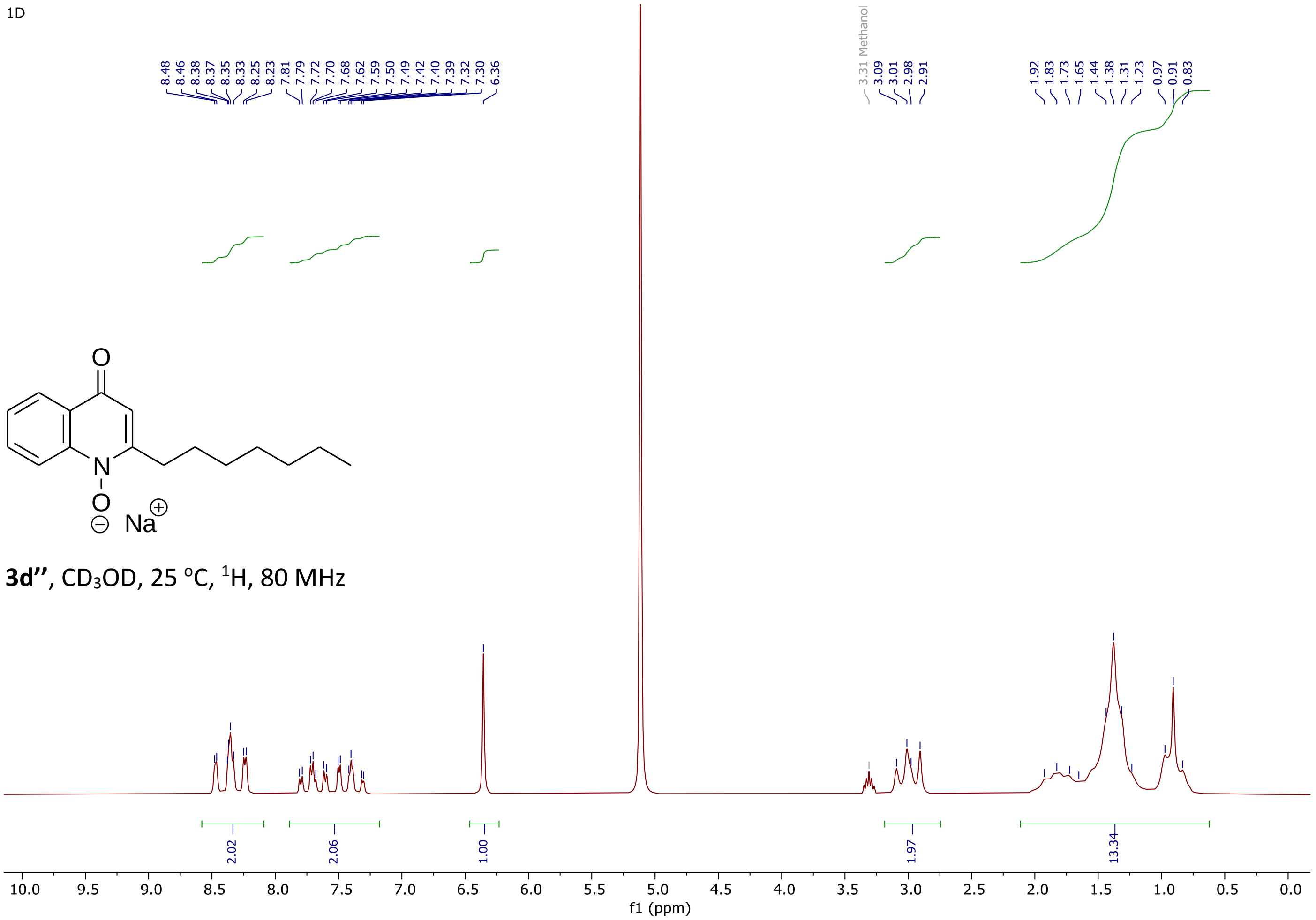

3d'', CD_3OD, 25 °C, ^1H, 80 MHz

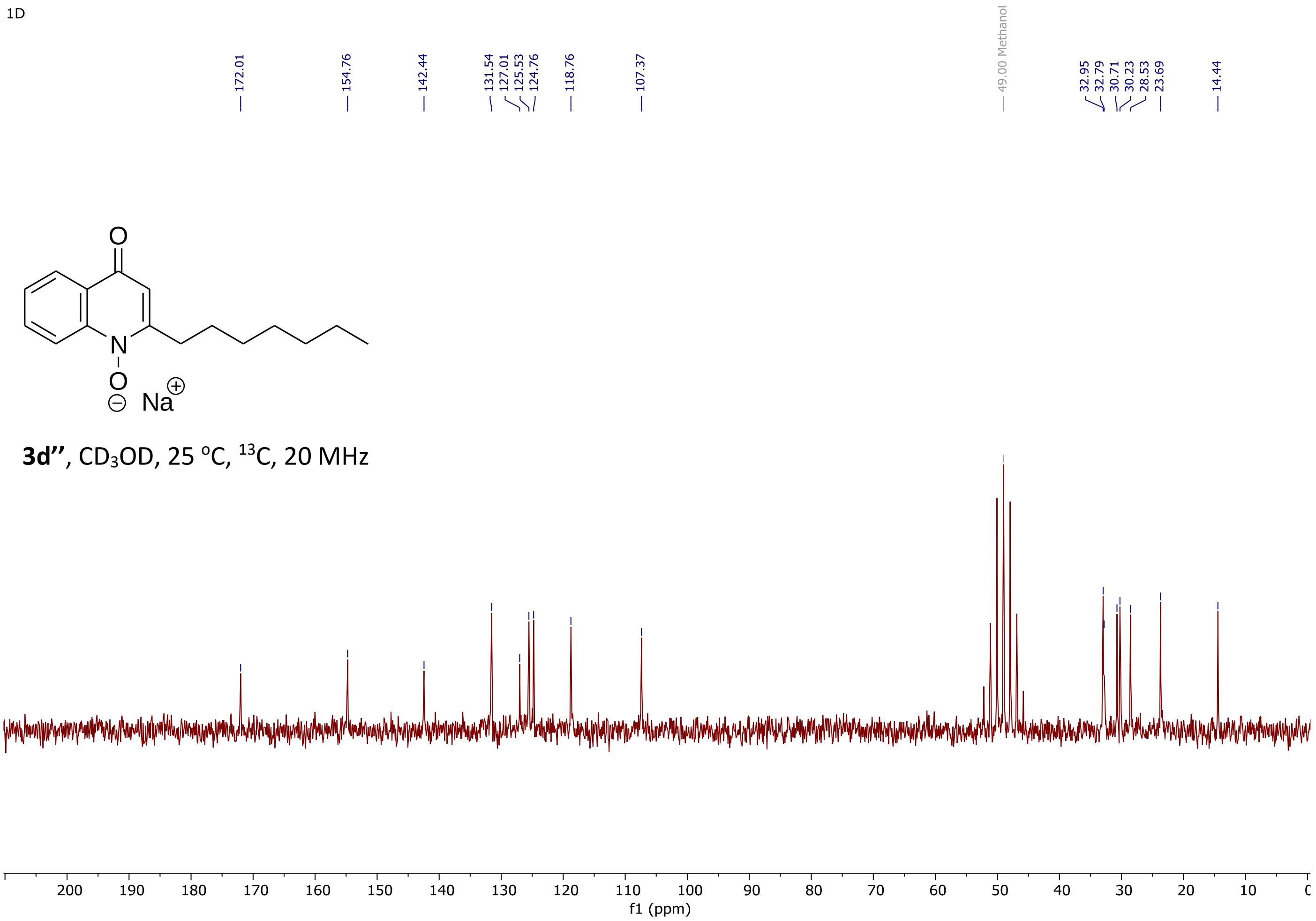

3d'', CD_3OD, 25 °C, ^{13}C, 20 MHz

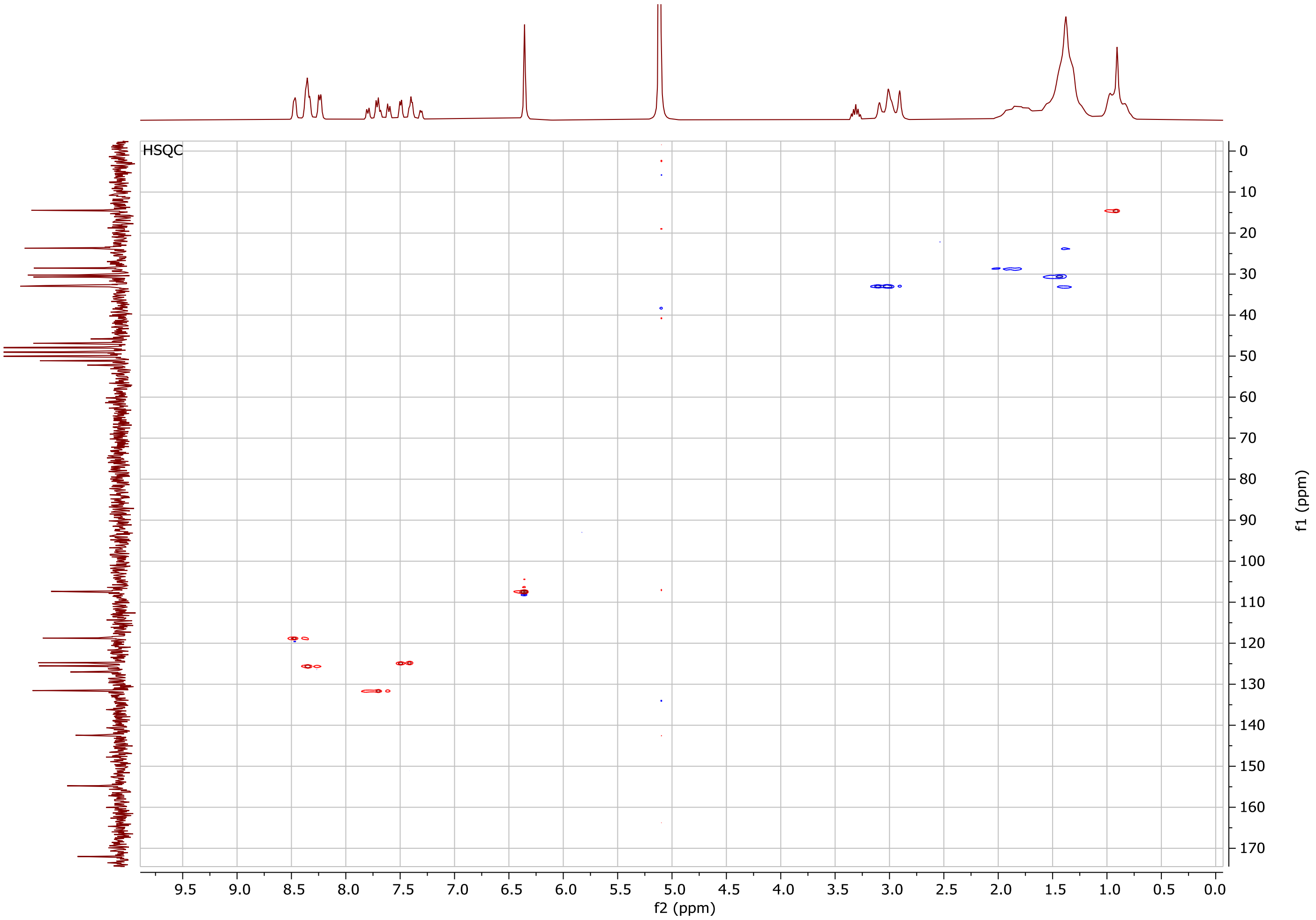
HSQC
f2 (ppm)
f1 (ppm)

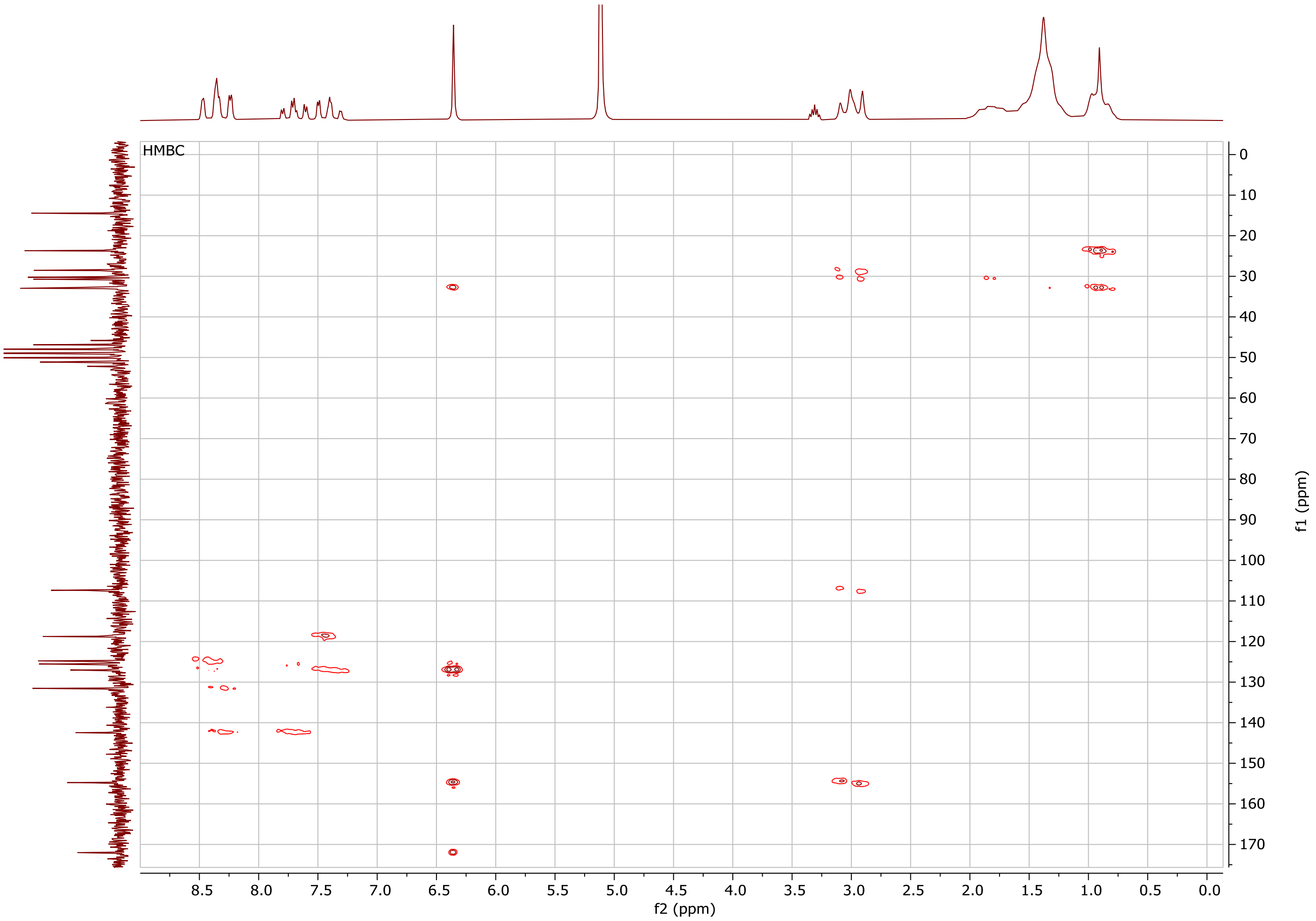
HMBC
f2 (ppm)
f1 (ppm)

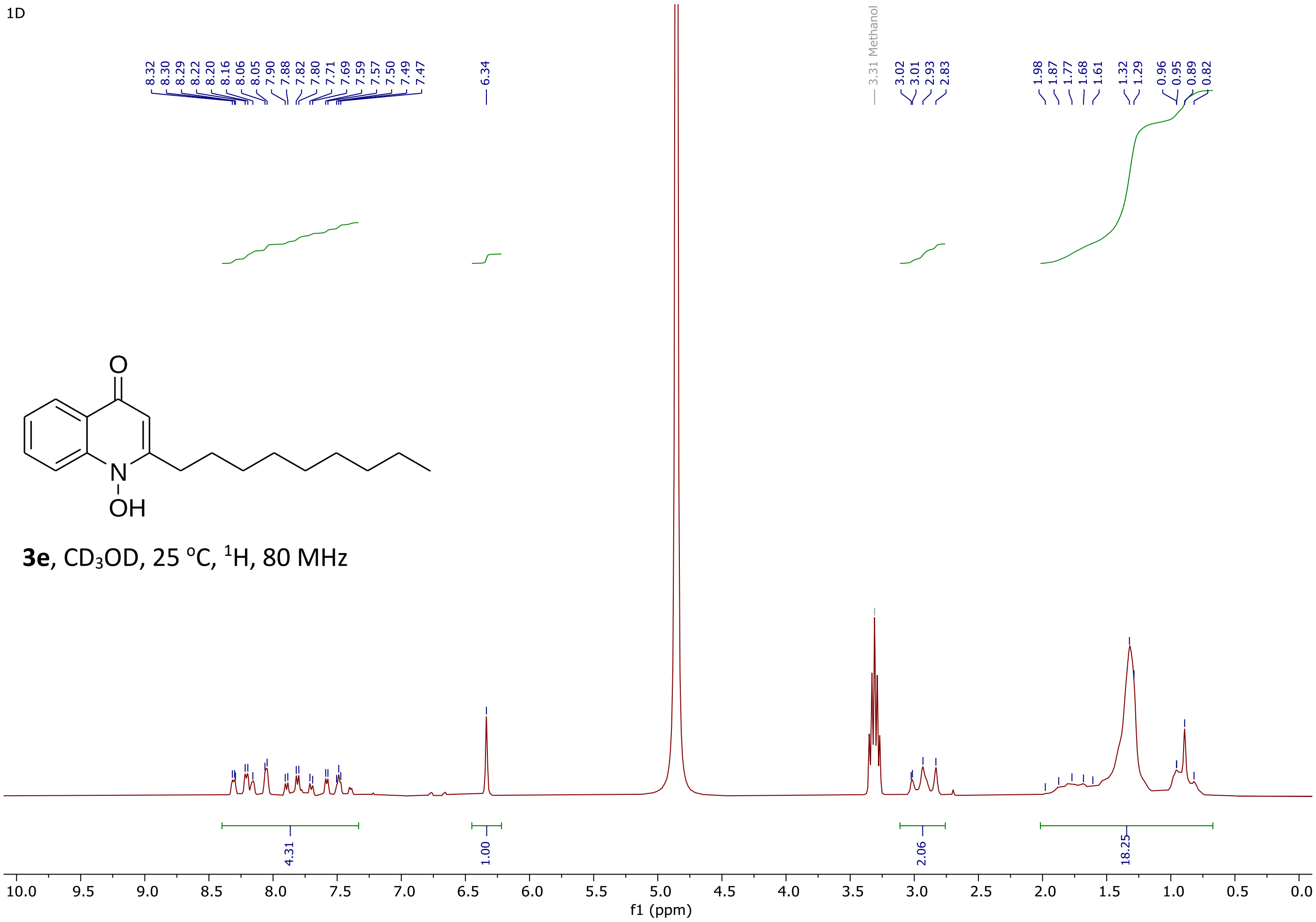

3e, CD_3OD, 25 °C, ^1H, 80 MHz

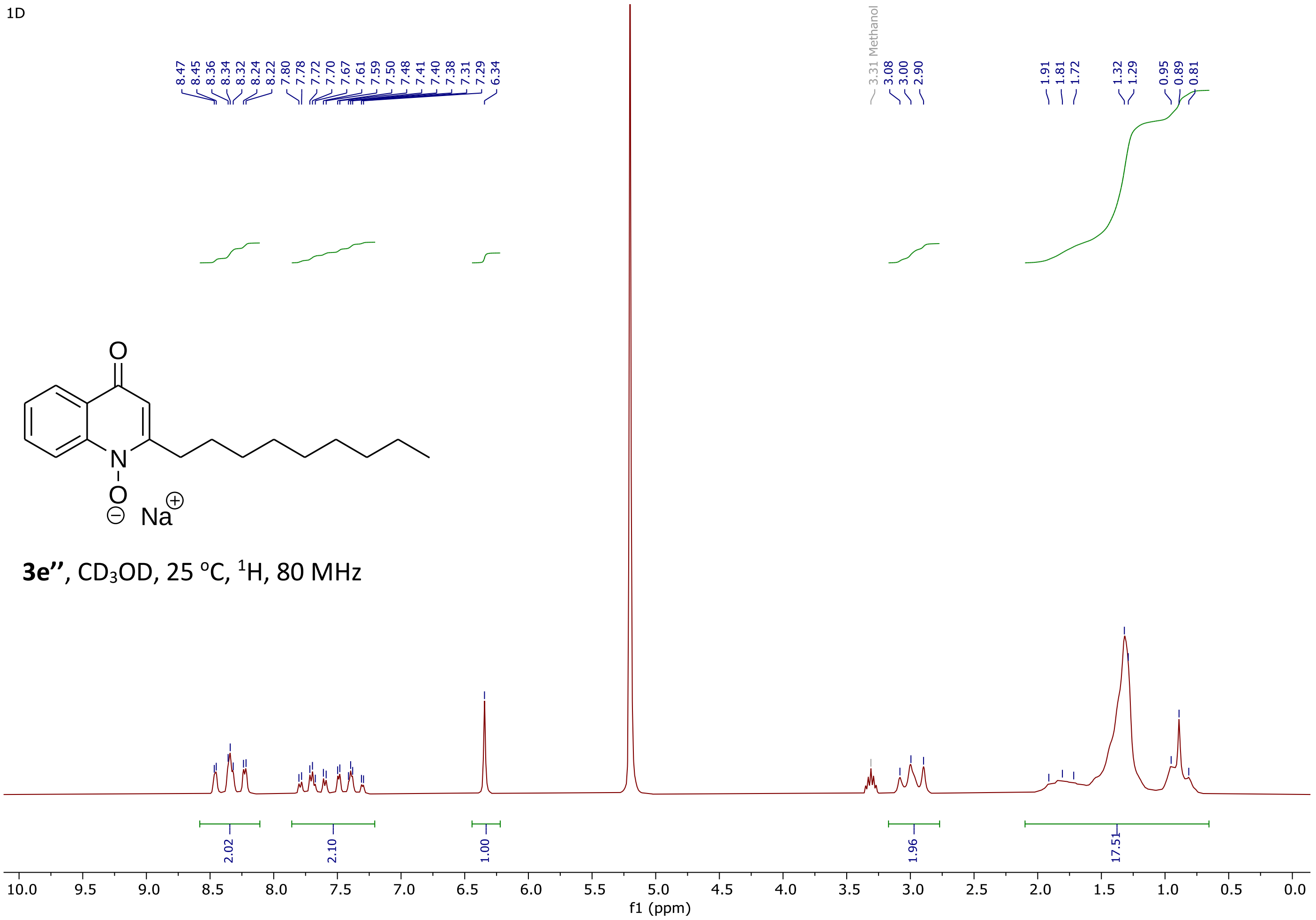
1D
8.47 8.45 8.36 8.34 8.32 8.24 8.22 7.80 7.78 7.72 7.70 7.67 7.61 7.59 7.50 7.48 7.41 7.40 7.38 7.31 7.29 6.34
3.31 Methanol
3.08 3.00 2.90
1.91 1.81 1.72
1.32 1.29
0.95 0.89 0.81
O
N
O
Na
3e'', CD3OD, 25 °C, 1H, 80 MHz
2.02
2.10
1.00
1.96
17.51
10.0 9.5 9.0 8.5 8.0 7.5 7.0 6.5 6.0 5.5 5.0 4.5 4.0 3.5 3.0 2.5 2.0 1.5 1.0 0.5 0.0
f1 (ppm)

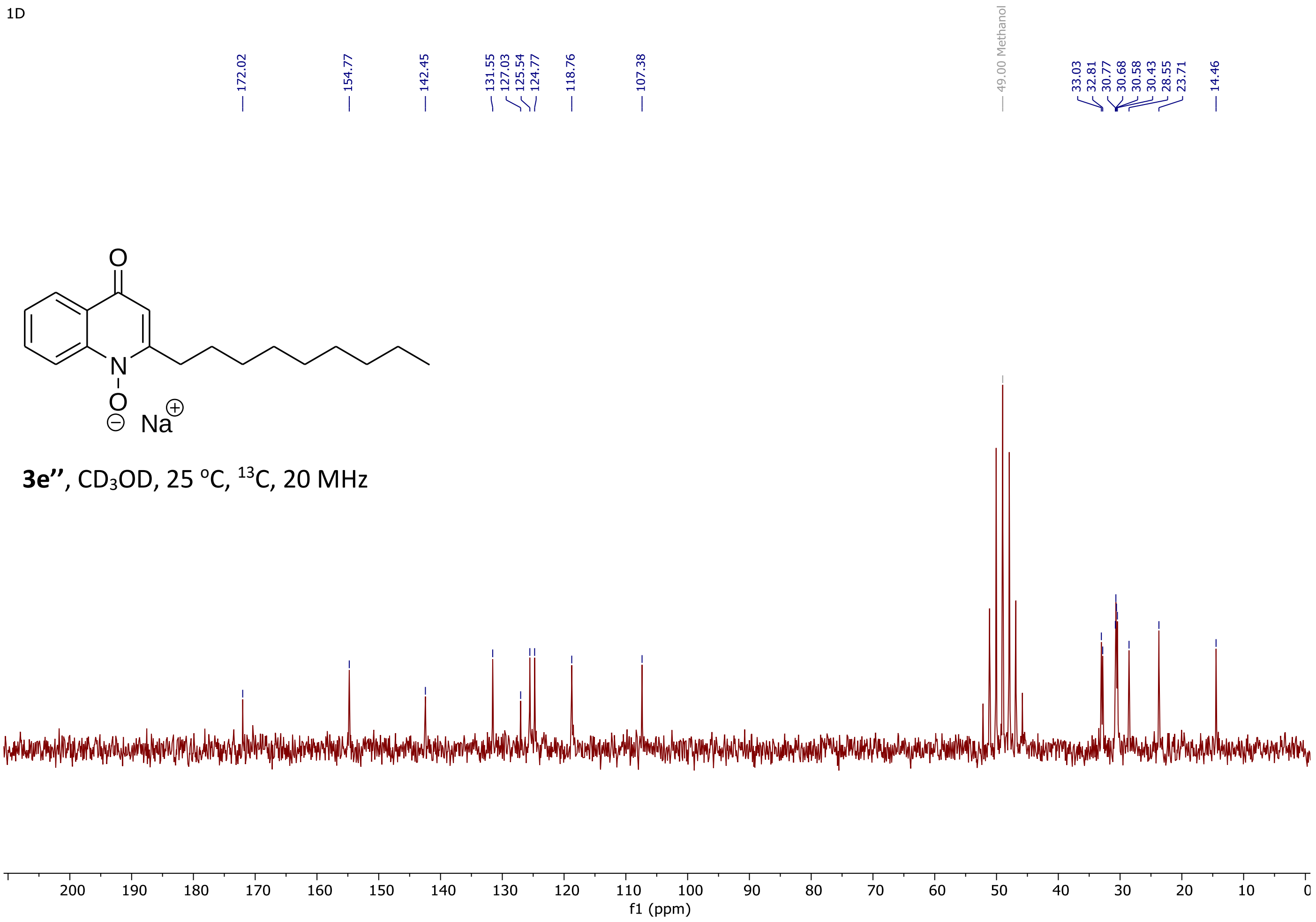
1D
172.02
154.77
142.45
131.55 127.03 125.54 124.77
118.76
107.38
49.00 Methanol
33.03 32.81 30.77 30.68 30.58 30.43 28.55 23.71
14.46
O
N
O
Na
3e'', CD3OD, 25 °C, 13C, 20 MHz
200 190 180 170 160 150 140 130 120 110 100 90 80 70 60 50 40 30 20 10 0
f1 (ppm)

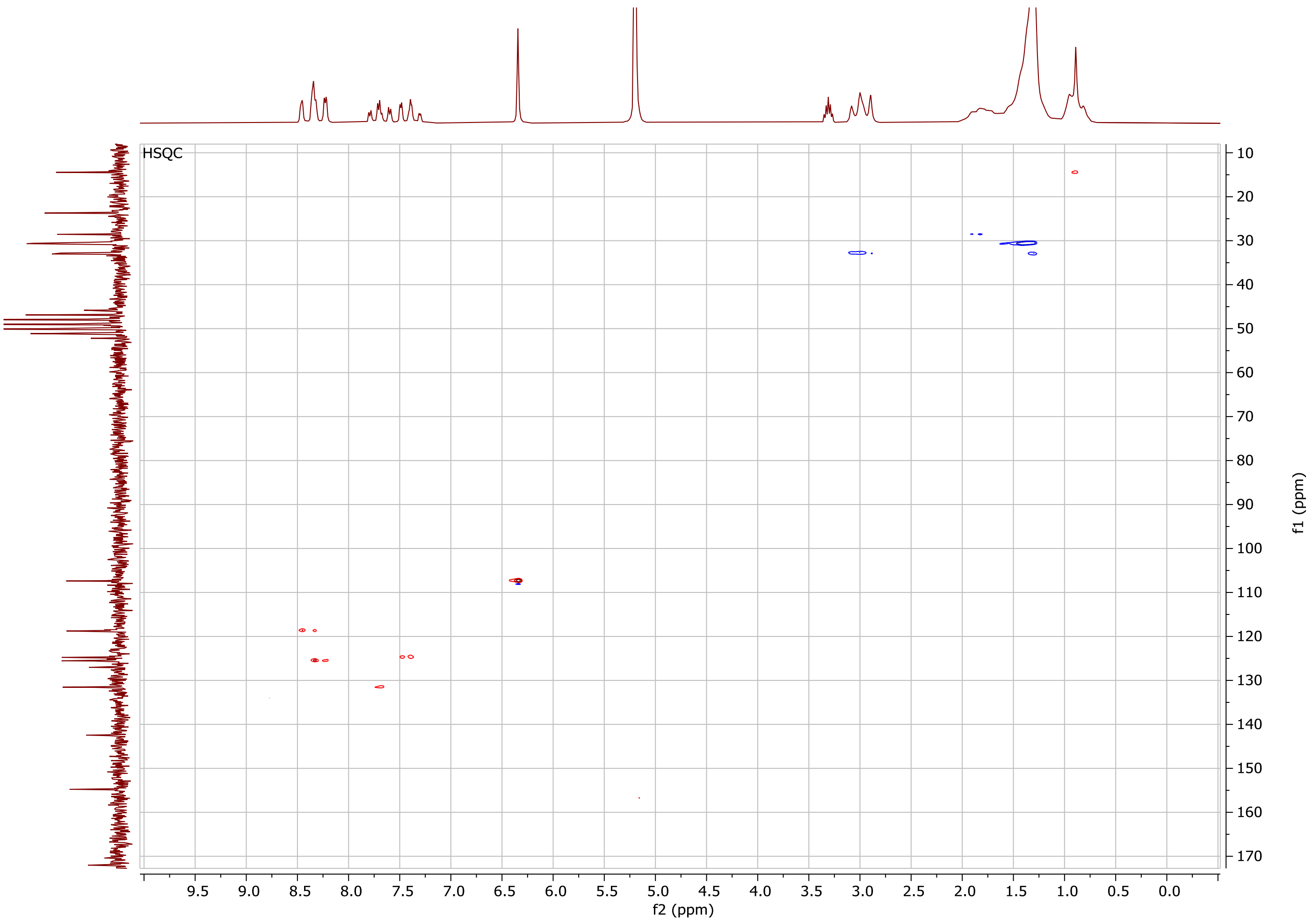
HSQC
f2 (ppm)
f1 (ppm)

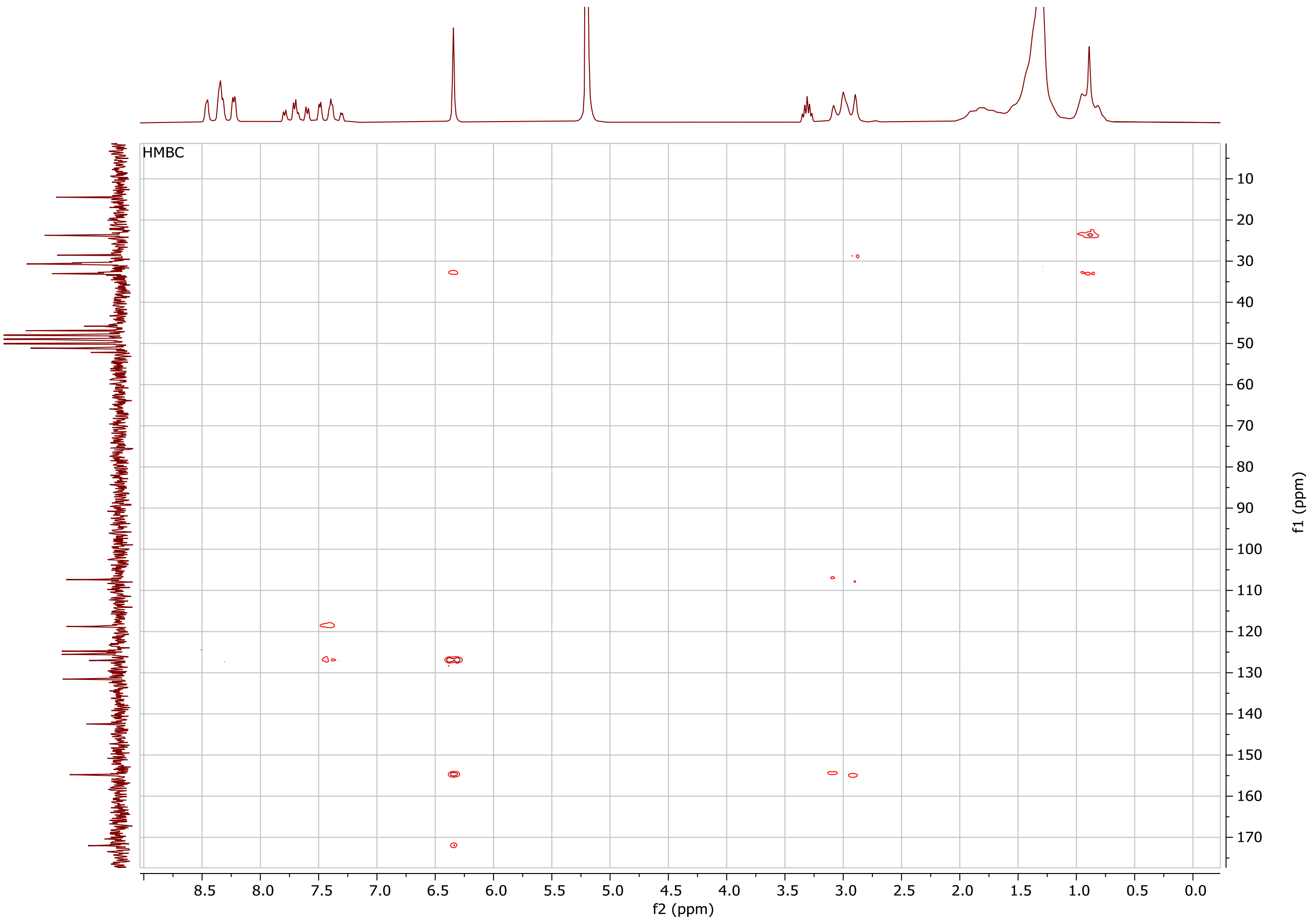
HMBC
f2 (ppm)
f1 (ppm)

HPLC traces and HRMS (ESI+) data

*1-Hydroxy-2-propylquinolin-4(1H)-one (**3a**):* calcd. for $C_{12}H_{14}NO_2^+$ $[M+H]^+$ 204.1019, found 204.1019

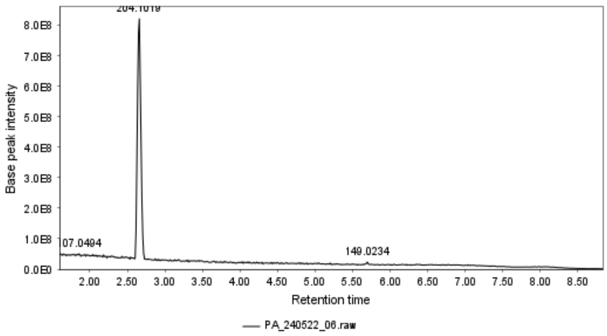

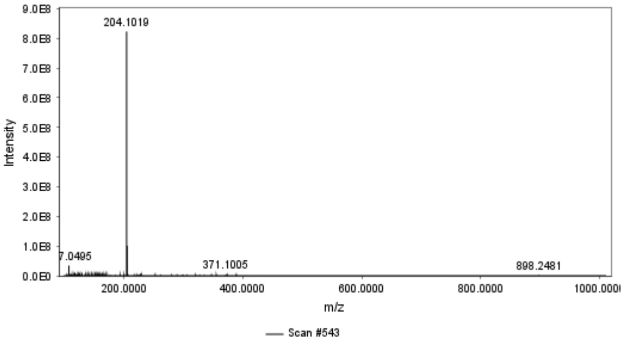

*1-Hydroxy-2-isobutylquinolin-4(1H)-one (**3b**):* calcd. for $C_{13}H_{16}NO_2^+$ $[M+H]^+$ 218.1176, found 218.1171

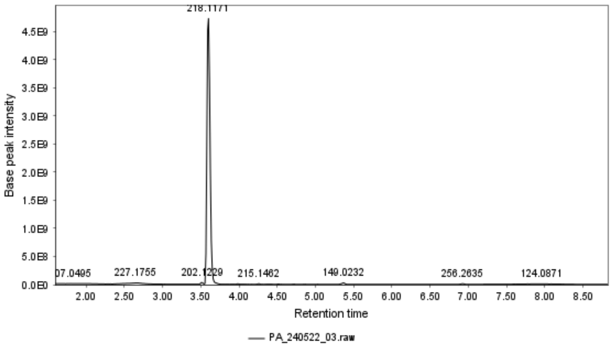

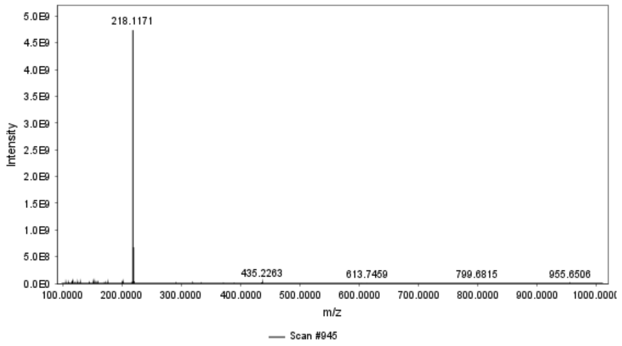

*1-Hydroxy-2-pentylquinolin-4(1H)-one (**3c**):* calcd. for $C_{14}H_{18}NO_2^+$ $[M+H]^+$ 232.1332, found 232.1331

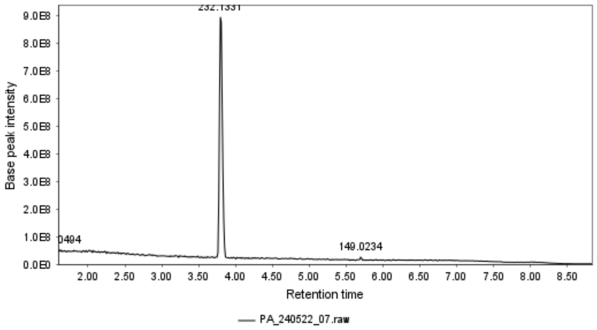

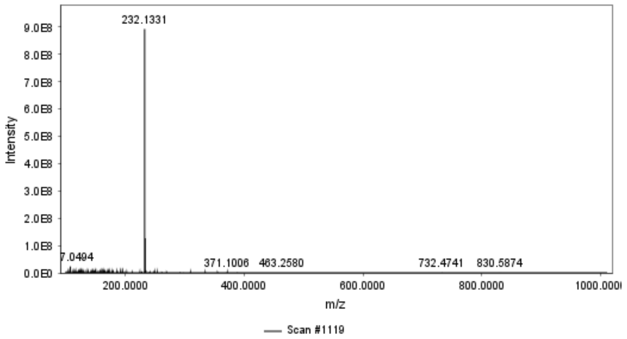

*1-Hydroxy-2-heptylquinolin-4(1H)-one (**3d**):* calcd. for $C_{16}H_{22}NO_2^+$ $[M+H]^+$ 260.1645, found 260.1636

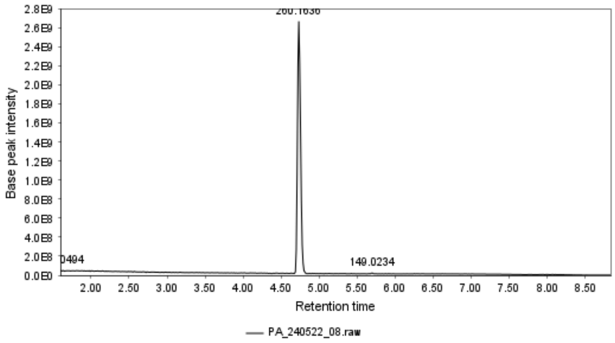

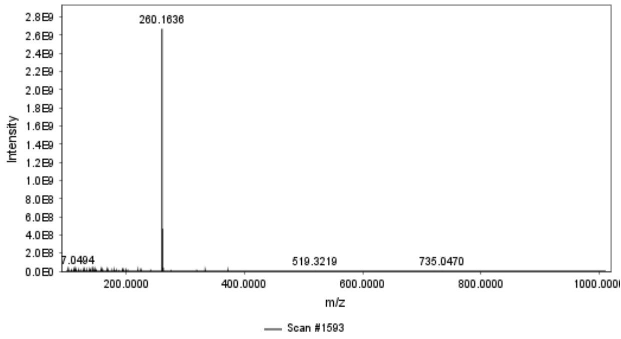

*1-Hydroxy-2-nonylquinolin-4(1H)-one (**3e**):* calcd. for $C_{18}H_{26}NO_2^+$ $[M+H]^+$ 288.1958, found 288.1954

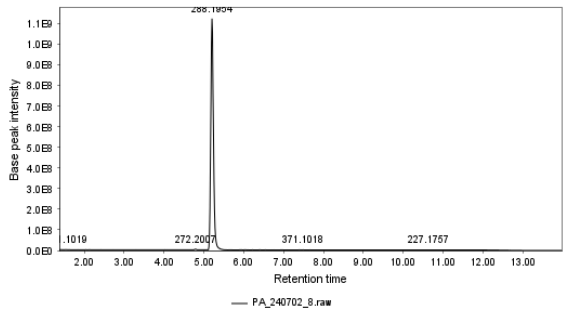

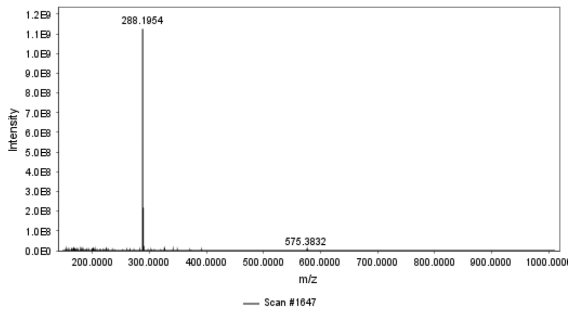

IR Spectra of compounds 3/3’ (Alpha II Bruker, KBr pellet)

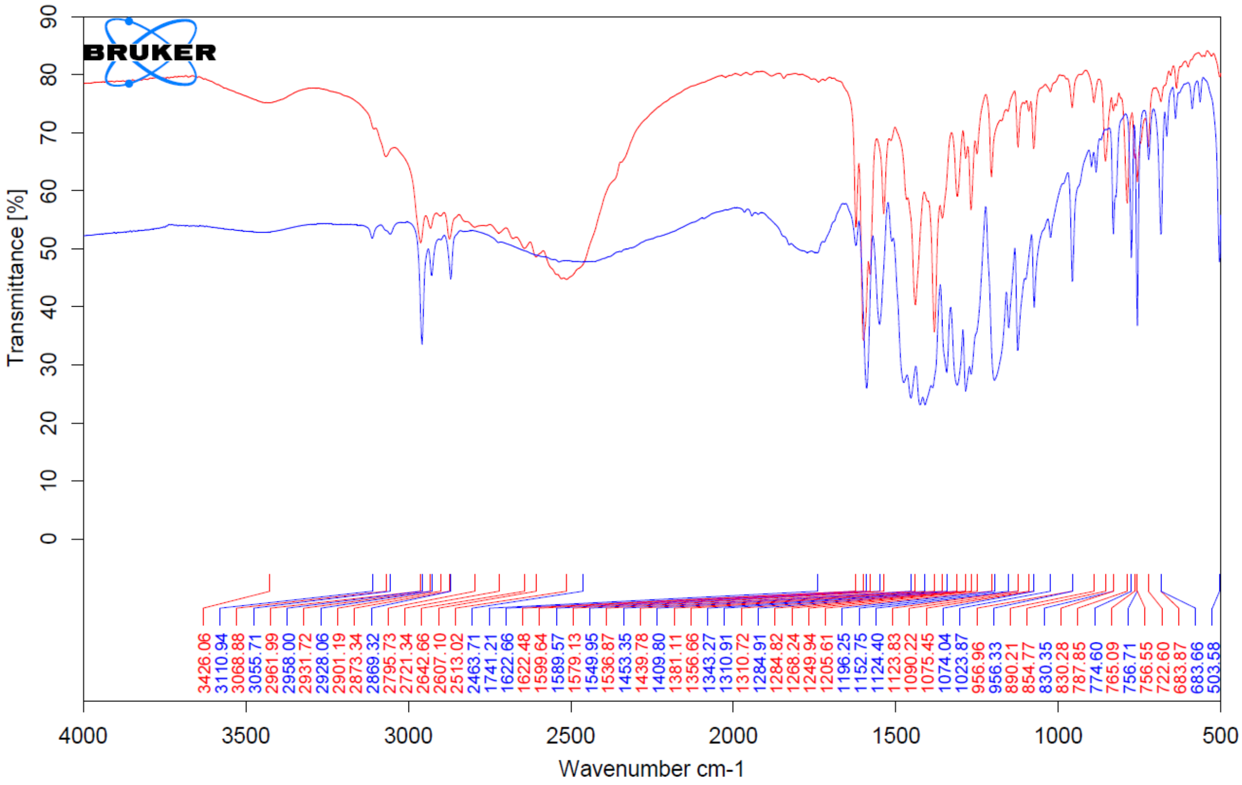

3a / 3a’

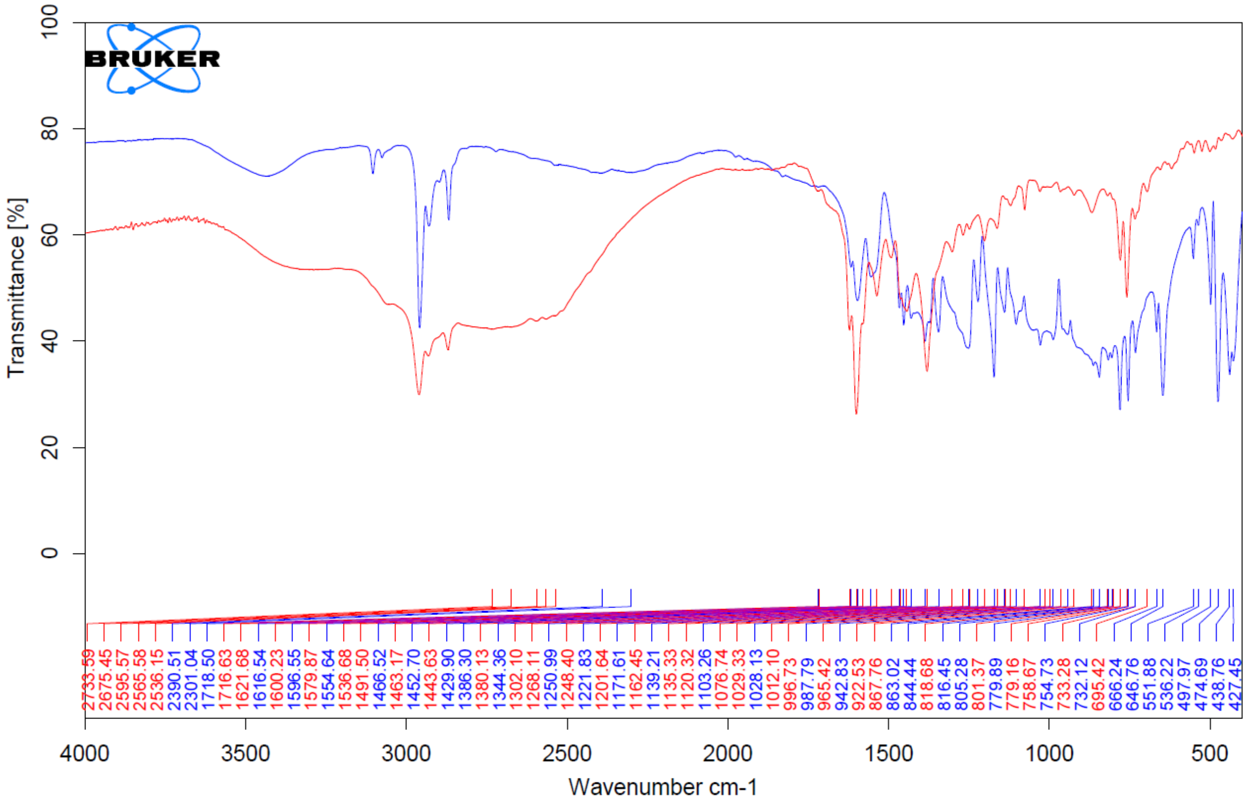

3b / 3b’

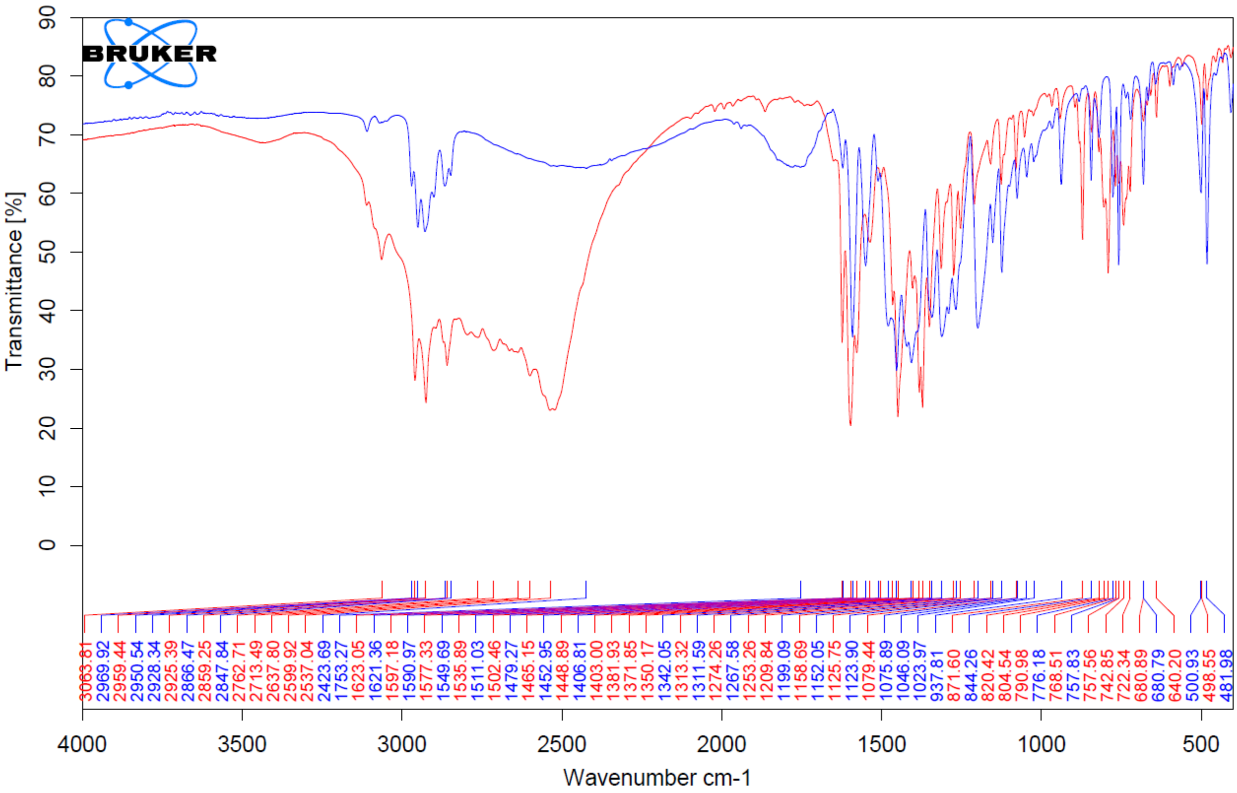
Transmittance [%]
Wavenumber cm-1
3063.81
2969.92
2959.44
2950.54
2928.34
2925.39
2866.47
2859.25
2847.84
2762.71
2713.49
2637.80
2599.92
2537.04
2423.69
1753.27
1623.05
1621.36
1597.18
1590.97
1577.33
1549.69
1535.89
1511.03
1502.46
1479.27
1465.15
1452.95
1448.89
1406.81
1403.00
1381.93
1371.85
1350.17
1342.05
1313.32
1311.59
1274.26
1267.58
1253.26
1209.84
1199.09
1158.69
1152.05
1125.75
1123.90
1079.44
1075.89
1046.09
1023.97
937.81
871.60
844.26
820.42
804.54
790.98
776.18
768.51
757.83
757.56
742.85
722.34
680.89
680.79
640.20
500.93
498.55
481.98

3c / 3c’

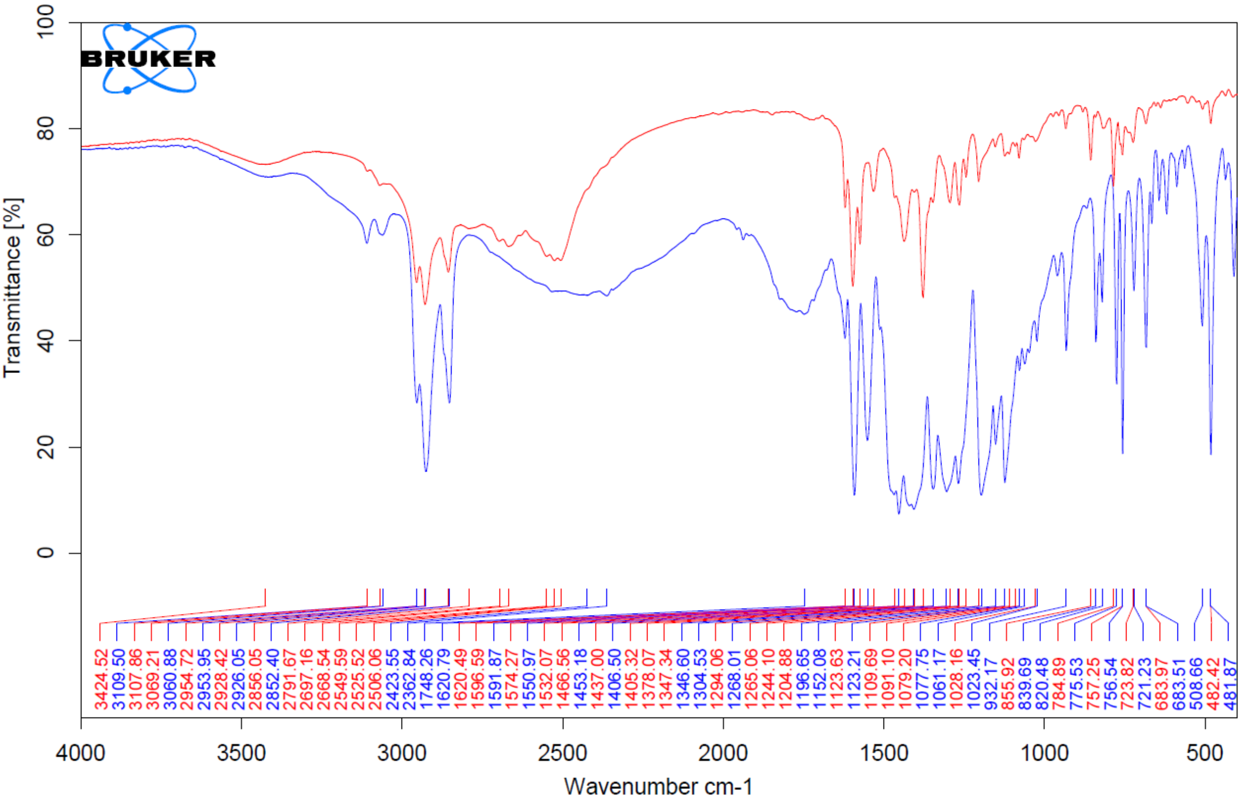
Transmittance [%]
Wavenumber cm-1
3424.52
3109.50
3107.86
3069.21
3060.88
2954.72
2953.95
2928.42
2926.05
2856.05
2852.40
2791.67
2697.16
2668.54
2549.59
2525.52
2506.06
2423.55
2362.84
1748.26
1620.79
1620.49
1596.59
1591.87
1574.27
1550.97
1532.07
1466.56
1453.18
1437.00
1406.50
1405.32
1378.07
1347.34
1346.60
1304.53
1294.06
1268.01
1265.06
1244.10
1204.88
1196.65
1152.08
1123.63
1123.21
1109.69
1091.10
1079.20
1077.75
1061.17
1028.16
1023.45
932.17
855.92
839.69
820.48
784.89
775.53
757.25
756.54
723.82
721.23
683.97
683.51
508.66
482.42
481.87

3d / 3d’

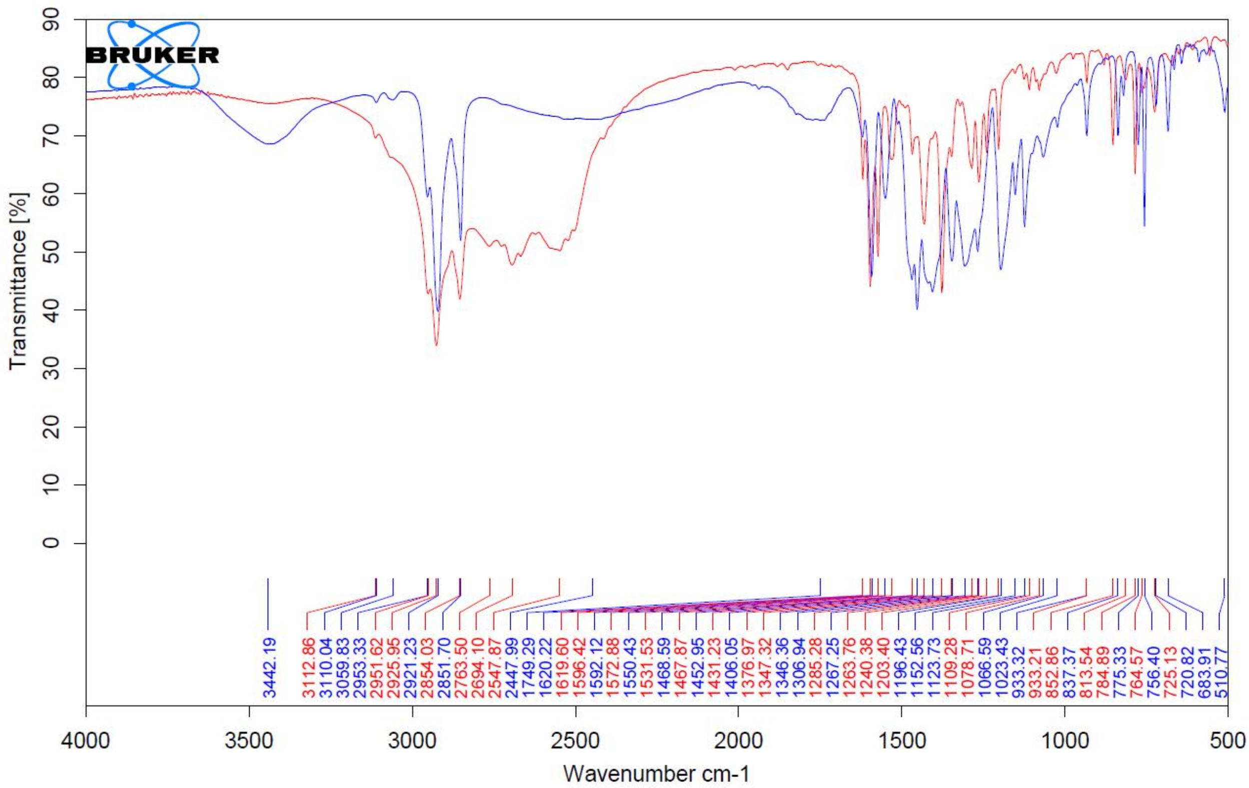

3e / 3e'

IR Spectra of sodium salts 3'' (Alpha II Bruker, ATR)

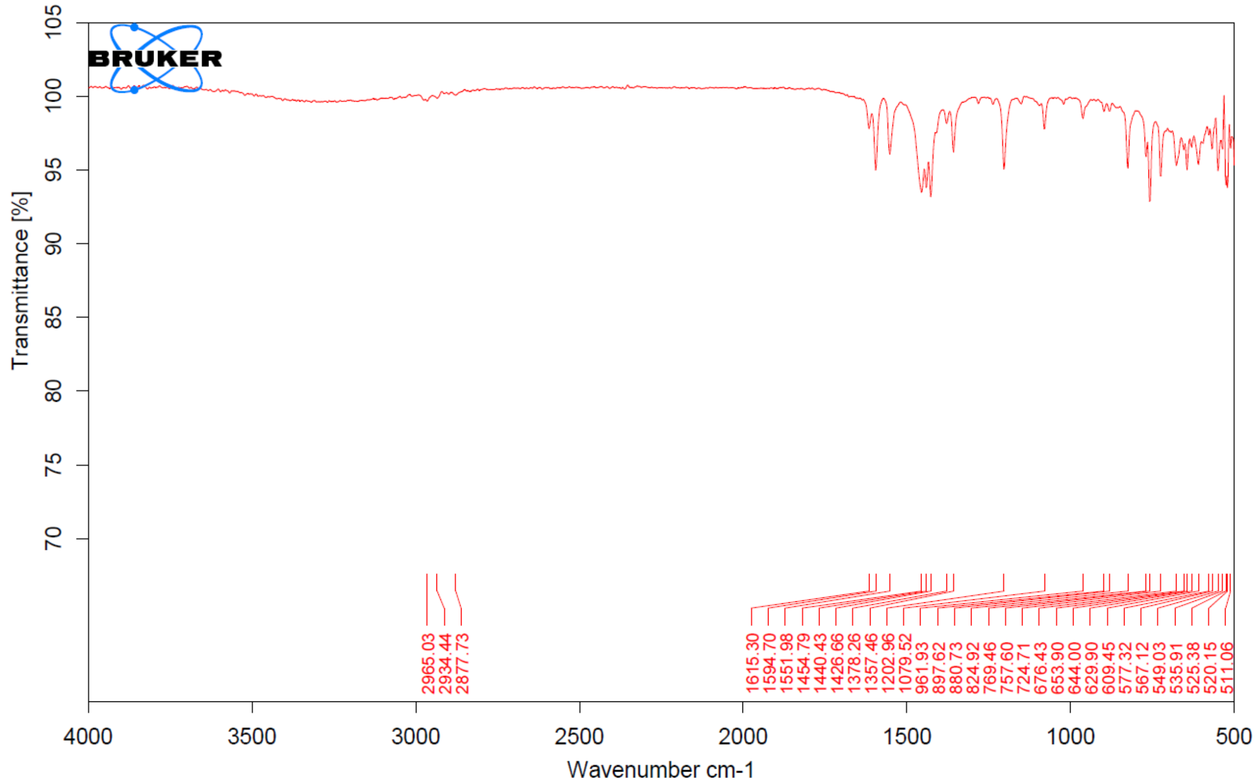

3a'' Na salt

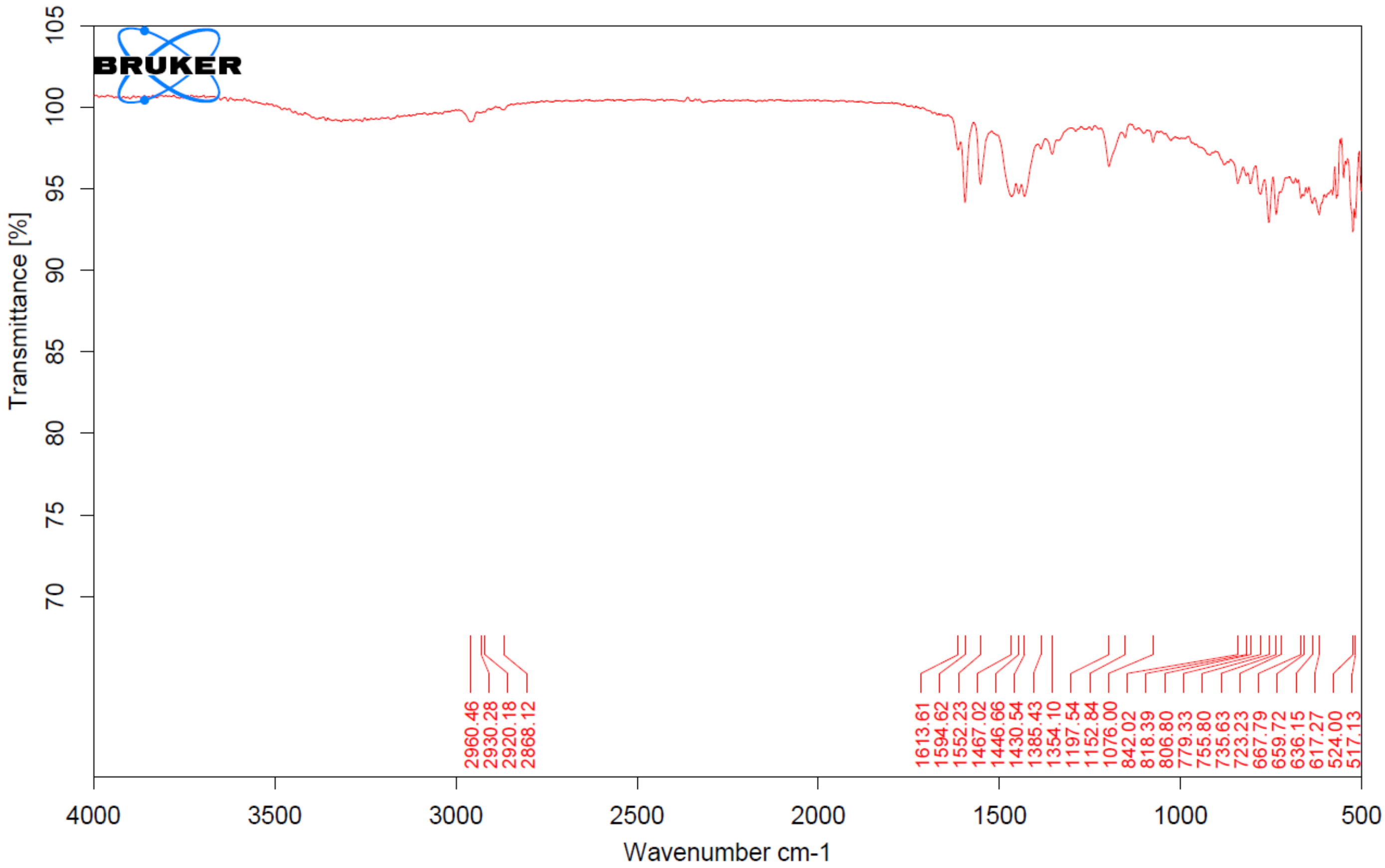

3b'' Na salt

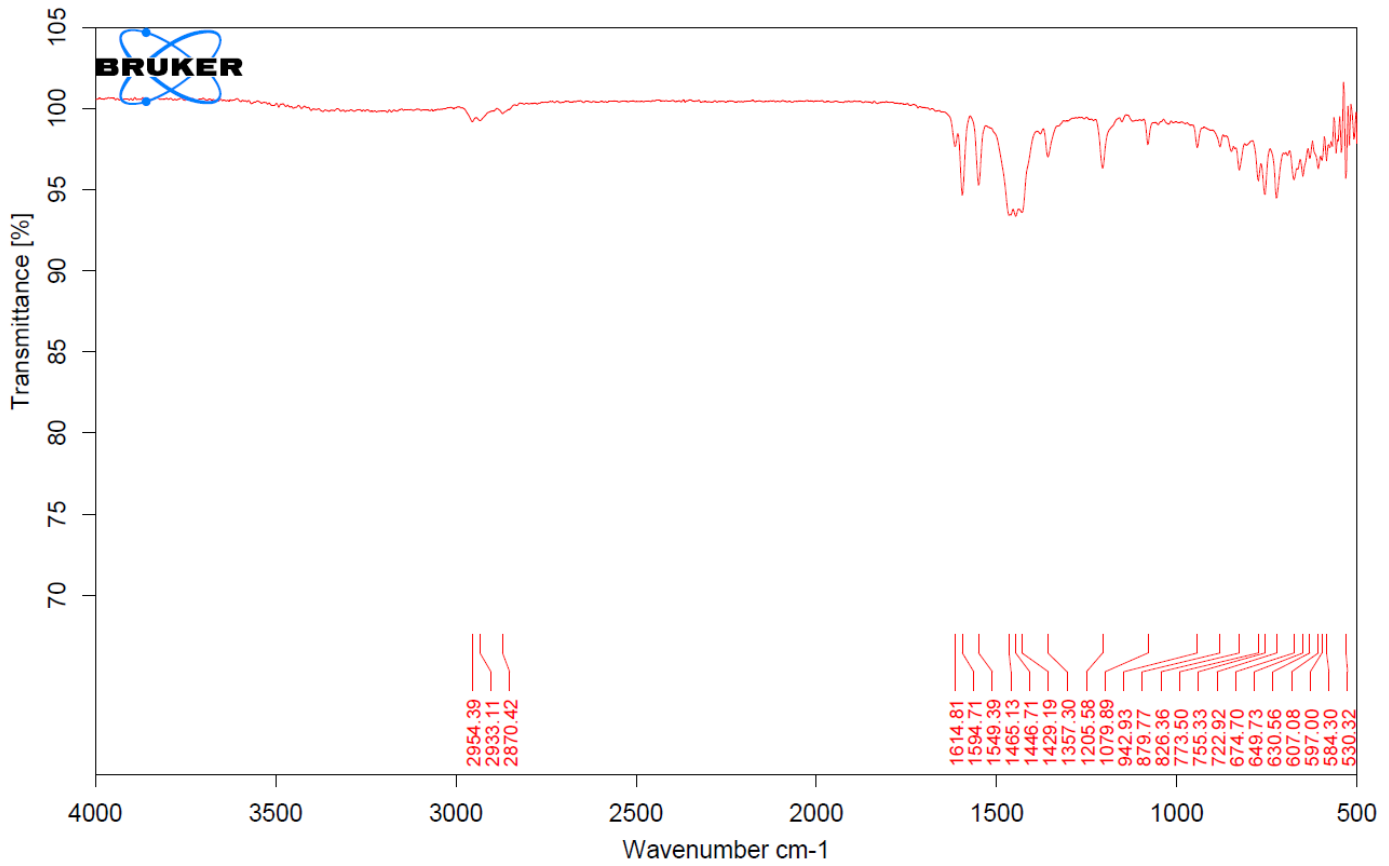

3c'' Na salt

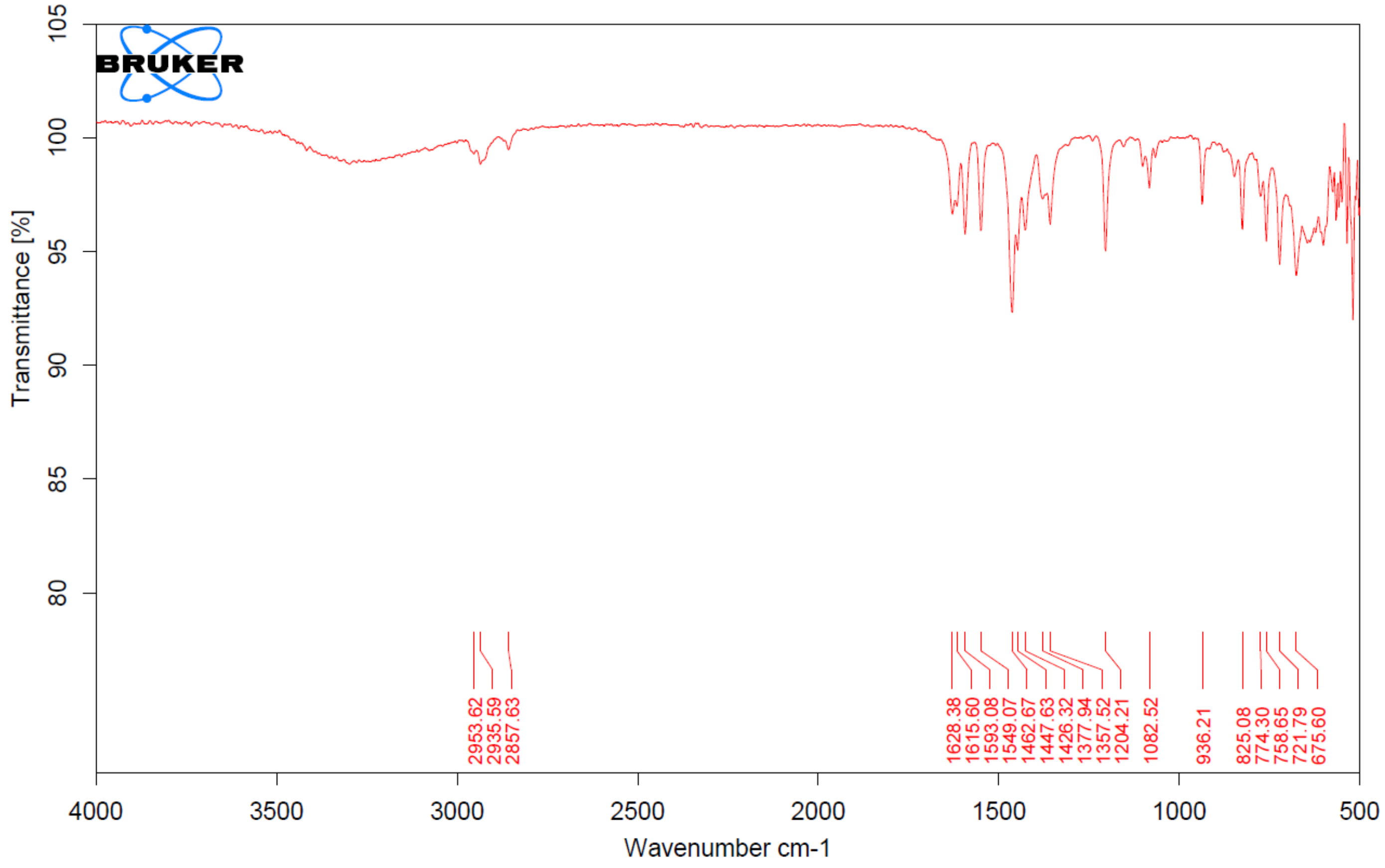

3d'' Na salt

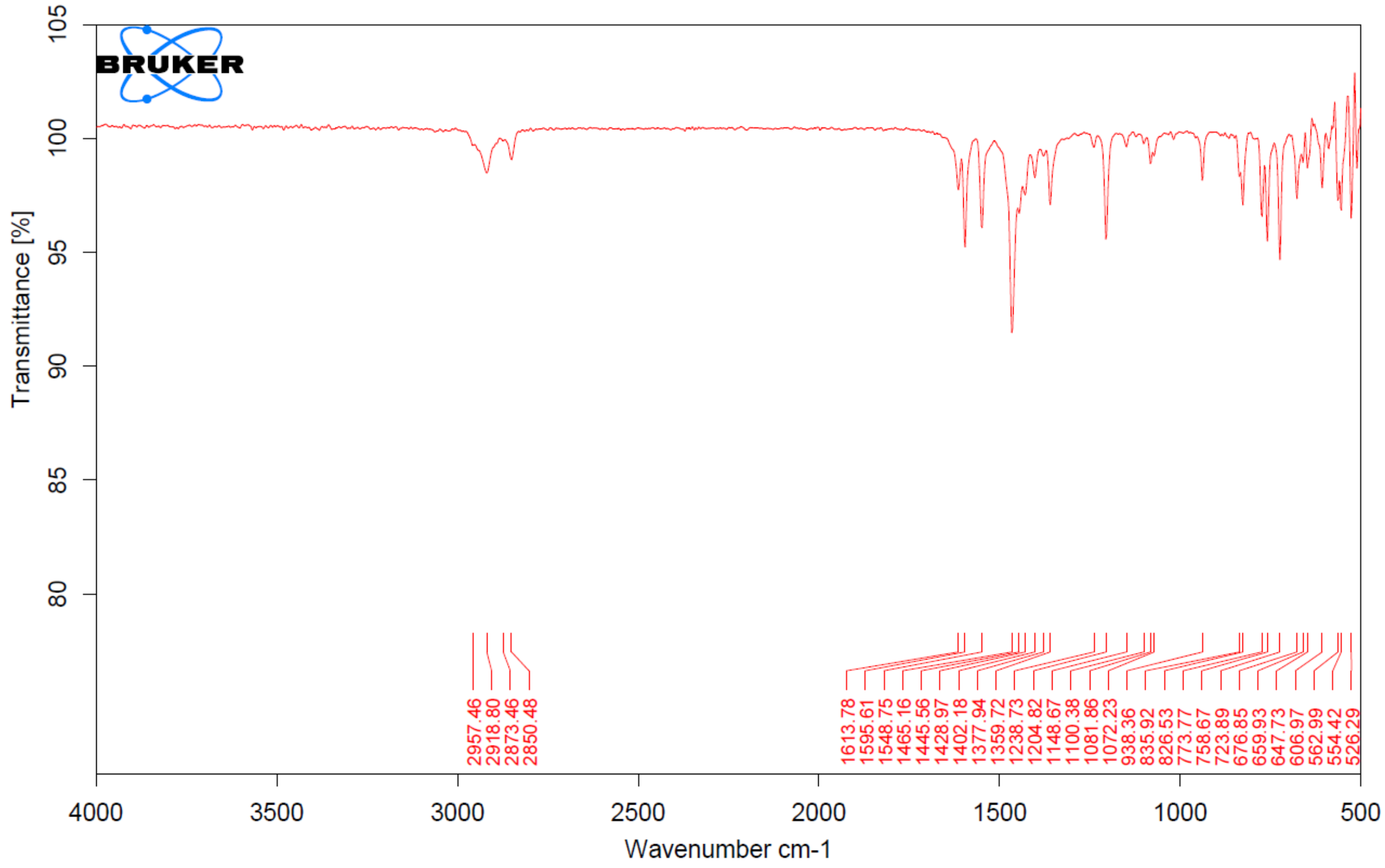

3e'' Na salt
